# Nasal Microbiota and Infectious Complications After Elective Surgical Procedures

**DOI:** 10.1001/jamanetworkopen.2021.8386

**Published:** 2021-04-29

**Authors:** Chiaowen Joyce Hsiao, Joseph N. Paulson, Sarabdeep Singh, Emmanuel F. Mongodin, Karen C. Carroll, Claire M. Fraser, Peter Rock, Nauder Faraday

**Affiliations:** 1 Department of Human Genetics, University of Chicago, Chicago, Illinois; 2 Product Development Biostatistics, Genentech, South San Francisco, California; 3 Center for Drug Evaluation and Research, Food and Drug Administration, White Oak, Maryland; 4 Institute for Genome Sciences, University of Maryland School of Medicine, Baltimore; 5 Lung Biology and Disease Program, Division of Lung Diseases, National Heart, Lung, and Blood Institute, National Institutes of Health, Bethesda, Maryland; 6 Division of Medical Microbiology, Department of Pathology, Johns Hopkins University School of Medicine, Baltimore, Maryland; 7 Department of Anesthesiology, University of Maryland School of Medicine, Baltimore; 8 Department of Anesthesiology and Critical Care Medicine, Johns Hopkins University School of Medicine, Baltimore, Maryland

## Abstract

**Importance:**

The association of the nasal microbiome with outcomes in surgical patients is poorly understood.

**Objective:**

To characterize the composition of nasal microbiota in patients undergoing clean elective surgical procedures and to examine the association between characteristics of preoperative nasal microbiota and occurrence of postoperative infection.

**Design, Setting, and Participants:**

Using a nested matched case-control design, 53 individuals who developed postoperative infection were matched (approximately 3:1 by age, sex, and surgical procedure) with 144 individuals who were not infected (ie, the control group). The 2 groups were selected from a prospective cohort of patients undergoing surgical procedures at 2 tertiary care university hospitals in Baltimore, Maryland, who were at high risk for postoperative infectious complications. Included individuals were aged 40 years or older; had no history of autoimmune disease, immunocompromised state, immune-modulating medication, or active infection; and were scheduled to undergo elective cardiac, vascular, spinal, or intracranial surgical procedure. Data were analyzed from October 2015 through September 2020.

**Exposures:**

Nasal microbiome cluster class served as the main exposure. An unsupervised clustering method (ie, grades of membership modeling) was used to classify nasal microbial samples into 2 groups based on features derived from 16S ribosomal RNA gene sequencing. The microbiome cluster groups were derived independently and agnostic of baseline clinical characteristics and infection status.

**Main Outcomes and Measures:**

Composite of surgical site infection, bacteremia, and pneumonia occurring within 6 months after surgical procedure.

**Results:**

Among 197 participants (mean [SD] age, 64.1 [10.6] years; 63 [37.7%] women), 553 bacterial taxa were identified from preoperative nasal swab samples. A 2-cluster model (with 167 patients in cluster 1 and 30 patients in cluster 2) accounted for the largest proportion of variance in microbial profiles using grades of membership modeling and was most parsimonious. After adjusting for potential confounders, the probability of assignment to cluster 2 was associated with 6-fold higher odds of infection after surgical procedure (odds ratio [OR], 6.18; 95% CI, 3.33-11.7; *P* < .001) independent of baseline clinical characteristics, including nasal carriage of *Staphylococcus aureus*. Intrasample (ie, α) diversity was inversely associated with infectious outcome in both clusters (OR, 0.57; 95% CI, 0.42-0.75; *P* < .001); however, probability of assignment to cluster 2 was associated with higher odds of infection independent of α diversity (OR, 4.61; 95% CI, 2.78-7.86; P < .001).

**Conclusions and Relevance:**

These findings suggest that the nasal microbiome was an independent risk factor associated with infectious outcomes among individuals who underwent elective surgical procedures and may serve as a biomarker associated with infection susceptibility in this population.

## Key Points

**Question:**

Can characterization of microbiota present on preoperative nasal swab estimate risk for postoperative infection in patients undergoing elective surgical procedures?

**Findings:**

In this case-control study, 167 patients were classified to nasal microbial profile cluster 1 and 30 patients to cluster 2 based on 16S ribosomal RNA gene sequencing. Classification to cluster 2 was associated with statistically significantly higher odds of infection (ie, deep surgical site infection, bacteremia, or pneumonia) after surgical procedure, independent of covariates, including nasal carriage of *Staphylococcus aureus* on preoperative culture and intrasample microbial diversity (ie, α diversity).

**Meaning:**

These findings suggest that the nasal microbiome is an independent risk factor associated with infectious outcomes after elective surgical procedures.

## Teaser

This case-control study investigates the association between nasal microbial profiles and odds of infection after elective surgical procedures.

## Introduction

The human nares in healthy individuals contains a rich diversity of microorganisms, including commensal, opportunistic, and pathogenic taxa.^1^ Environmental and genetic factors are reported to be associated with interindividual variability in the composition of nasal microbiota; however, the association of this variability with health and disease is poorly understood.^1,2^ Decreased diversity levels within the microbial niche (ie, α diversity) of the gut are associated with clinical disease outside the gut, including obesity and diabetes, and with death^3,4^; however, the association between features of nasal microbiota and clinical outcomes not involving the nose or sinuses has not been reported.

The presence of *Staphylococcus aureus* among the microbiota of the anterior nares has garnered substantial attention because of this microorganism’s pathogenic potential and known association with clinical infection at non-nasal sites.^5^ For example, patients who test positive for *S aureus* on preoperative nasal culture are at 2-fold to 9-fold increased risk of postoperative surgical site infection (SSI),^6^ and nasal colonization is associated with increased risk of blood stream infection^7^ and pneumonia^8^ in patients admitted to the hospital. *S aureus* decolonization before surgical procedure is associated with decreased risk of postoperative SSI; however, protection is incomplete.^9,10^ Numerous bacteria compete for the ecologic niche of the anterior nares, and species other than *S aureus* may contribute, either directly or indirectly, to the association between *S aureus* and infectious risk.

This study had 3 main aims: to thoroughly characterize the microbiota present on nasal swab samples obtained before elective surgical procedure using state of the art bacterial gene profiling; to classify individuals into cluster groups, independent and agnostic of postoperative outcomes, based solely on preoperative microbial profiles; and to evaluate the association between microbial clusters and development of postoperative infection. In this report, we describe the microbiologic characteristics that define the cluster groups, the association of cluster with baseline clinical characteristics and *S aureus* nasal colonization, and the associations between microbial features of the clusters and the occurrence of non-nasal infectious complications after surgical procedure.

## Methods

This case-control study was approved by the institutional review boards at Johns Hopkins Hospital and the University of Maryland, Baltimore. All participants signed written informed consent. This study is reported following the Strengthening the Reporting of Observational Studies in Epidemiology (STROBE) reporting guideline.

### Study Design and Participants

We conducted a nested matched case-control study involving participants selected from a prospective cohort study of elective high-risk clean surgical procedures (ie, cardiac, vascular, spinal fusion, and craniotomy procedures).^11^ Among 802 participants, 53 individuals with serious postoperative infection were identified and matched approximately 3 to 1 by age (ie, older or younger by 5 years), sex, and surgical procedure with 144 individuals who were not infected who served as controls; financial constraints limited microbial analyses to a small subset of the entire cohort. Participants were accrued into the original study cohort at the Johns Hopkins Hospital and University of Maryland Medical Center. Full details regarding setting, participants, and protocol of the original cohort are described in detail elsewhere^11^ and in the eAppendix in the Supplement.

### Identification of *S aureus* and Other Microbiota From the Anterior Nares

Nasal swab of the anterior nares was obtained from each participant at baseline before surgical procedure and use of antibiotics. The presence or absence of *S aureus* was determined in the clinical microbiology laboratory by standard culture. Isolation of bacterial DNA from nasal swabs,^12,13^ 16S ribosomal RNA (rRNA) gene profiling of the V3V4 hypervariable region,^14,15^ read processing,^13^ and taxonomic classification^16,17^ of microbiota were performed as previously described (eAppendix in the Supplement).

### Main Exposure

Nasal microbiome cluster class served as the main exposure. We used an unsupervised clustering method (ie, grades of membership model^18,19^) to classify nasal microbial samples based solely on features derived from 16S rRNA gene sequencing. Using this method, microbiome cluster classification was derived independently and agnostic of baseline clinical characteristics and infection status. The grades of membership model allows each sample to have some proportion of its membership, or partial membership, in each cluster. We used these partial membership weights to assign each sample to a cluster and estimated the optimal number of clusters using log Bayes factor. We implemented our analysis using the CountClust package (version 1.4.1) in R statistical software version 3.4.1 (R Project for Statistical Computing)^18^ (eAppendix in the Supplement).

### Outcomes and Covariates

The primary outcome was a composite of deep SSI, pneumonia, or bacteremia, as defined by Centers for Disease Control and Prevention surveillance criteria,^20^ occurring within 6 months postoperatively. Secondary outcomes were SSI, pneumonia, and bacteremia separately; the composite outcome at 30 days; and death at 6 months. Baseline covariates and outcomes were determined by participant interview and medical record review. A full list of variables and definitions is available in the eAppendix in the Supplement.

### Statistical Analysis

Differential abundance of microbial taxa at the aggregate and individual levels was determined from counts of rRNA sequences annotated to the species level. We accounted for variability^21^ and sparsity^22^ in sequence data as previously described. Differential abundance results are reported in terms of false discovery rate with *q* < .05 considered significant. The association of taxa abundance with nasal carriage of *S aureus* was determined by logistic regression using log-transformed taxa counts as an independent variable. Within-sample diversity (ie, α) and between-sample diversity (ie, β) were calculated (eAppendix in the Supplement).

Baseline categorical covariates were expressed as percentages; differences between cluster classes were compared using the χ^2^ or Fisher exact tests. Continuous variables were expressed using mean (SD), and cluster classes were compared using *t* or Kruskal-Wallis tests. A series of generalized linear regression models were performed to assess the association of the primary exposure (ie, probability of assignment to cluster 2) and covariates with the primary and secondary outcomes unadjusted and adjusted for potential confounding baseline covariates. Adjusted model 1 was adjusted for demographic covariates: age, sex, and race. Adjusted model 2 was adjusted for baseline comorbidities: congestive heart failure, peripheral vascular disease, chronic obstructive pulmonary disease, history of smoking, history of cancer, and Charlson Comorbidity Index score. Adjusted model 3 was adjusted for surgical procedure–associated variables: study site, inpatient or outpatient status, and surgical procedure. Adjusted model 4 was adjusted for nasal culture results for *S aureus* and for methicillin-resistant *S aureus*. Adjusted model 5 was adjusted for inverse probability of treatment weighting (IPTW) using propensity score for assignment to microbiome cluster 2. The propensity score for assignment to cluster 2 was generated by incorporating all baseline covariates listed in Table 1. We used IPTW (with treatment defined as assignment to cluster 2) to adjust for all baseline covariates using the propensity score. Propensity score analyses were bootstrapped using 500 subsamples from approximately 70% of all samples comprising the study group (eAppendix in the Supplement). We examined the association of infection with α diversity and abundance of individual microbial taxa using logistic regression.

**Table 1.** Baseline Characteristics of Study Participants by Microbiome Cluster Class

Characteristic	No. (%)		*P* value
	Cluster 1 (n = 167)	Cluster 2 (n = 30)	
Demographic characteristic			
Age, mean (SD), y	64.10 (10.91)	63.83 (9.04)	.88
Sex			
Women	63 (37.7)	9 (30.0)	.54
Men	104 (62.3)	21 (70.0)	
Race			
White	142 (85.0)	26 (87.7)	.99
Black	24 (14.4)	4 (13.3)	
Asian	1 (0.6)	0	
Comorbidity			
Obesity	67 (40.1)	9 (30.0)	.39
Diabetes	29 (17.4)	6 (20.0)	.86
Hypertension	117 (70.1)	19 (63.3)	.60
Myocardial Infarction	26 (15.6)	6 (20.0)	.59
Congestive heart failure	14 (8.3)	6 (20.0)	.09
Peripheral vascular disease	15 (9.0)	5 (16.7)	.19
Cerebrovascular disease	17 (10.2)	5 (16.7)	.34
COPD	11 (6.6)	1 (3.3)	.69
History of smoking	108 (64.7)	20 (66.7)	.99
Gastric ulcer	12 (7.2)	2 (6.7)	.99
Chronic liver disease	2 (1.2)	0	.99
Dialysis dependency	2 (1.2)	0	.99
History of cancer	17 (10.2)	7 (23.3)	.06
Infection treated with antibiotics in previous year	54 (32.3)	11 (36.7)	.79
Hospitalization in previous year	54 (32.3)	13 (43.3)	.36
American Society of Anesthesiologists class			
2	32 (19.2)	6 (20.0)	.96
3	98 (58.7)	18 (60.0)	
4	37(22.2)	6 (20.0)	
Charlson Comorbidity Index score			
0-2	65 (38.9)	8 (26.7)	.16
3-4	53 (31.7)	8 (26.7)	
>5	49 (29.3)	14 (46.7)	
Surgical factors associated with risk of infection			
Inpatient at the time of surgical procedure	41 (24.6)	9 (30.0)	.68
Surgical procedure			
Cardiac	73 (43.7)	12 (40.0)	.73
Vascular	18 (10.8)	5 (16.7)	
Spinal	52 (31.1)	8 (26.7)	
Intracranial	24 (14.4)	5 (16.7)	
Study site			
Johns Hopkins Hospital	149 (89.2)	30 (100)	.08
University of Maryland Medical Center	18 (10.8)	0	
Nasal culture for *S aureus*			
*S aureus* positive	35 (21.0)	6 (20.0)	.99
Methicillin-resistant *S aureus* positive	7 (4.2)	2 (6.7)	.62

Abbreviations: COPD, chronic obstructive pulmonary disease; *S aureus*, *Staphylococcus aureus*.

Statistical significance was set at *P* < .05, and all tests were 2-sided. Benjamini-Hochberg false discovery procedure was used to correct for multiple comparisons. Data analysis was conducted from October 2015 through September 2020.

## Results

Among 197 included patients, 53 individuals had a postoperative infection (29.7%) and 144 individuals did not have infections (ie, the control group; 73.1%). Mean (SD) age was 64.1 (10.6) years, 63 (37.7%) were women, and 24 individuals were Black (14.4%). Among all participants, 41 individuals (20.8%) tested positive for *S aureus* on preoperative nasal culture, and 9 of these individuals (22.0%) tested positive for methicillin-resistant *S aureus*. A total of 4423 operational taxonomic units were identified by 16S rRNA gene sequencing from the 197 nasal swab samples obtained before surgical procedure. These were organized into 477 distinct taxa to the genus level and 553 taxa to the species level (eFigure 1 in the Supplement).

**Figure 1.**
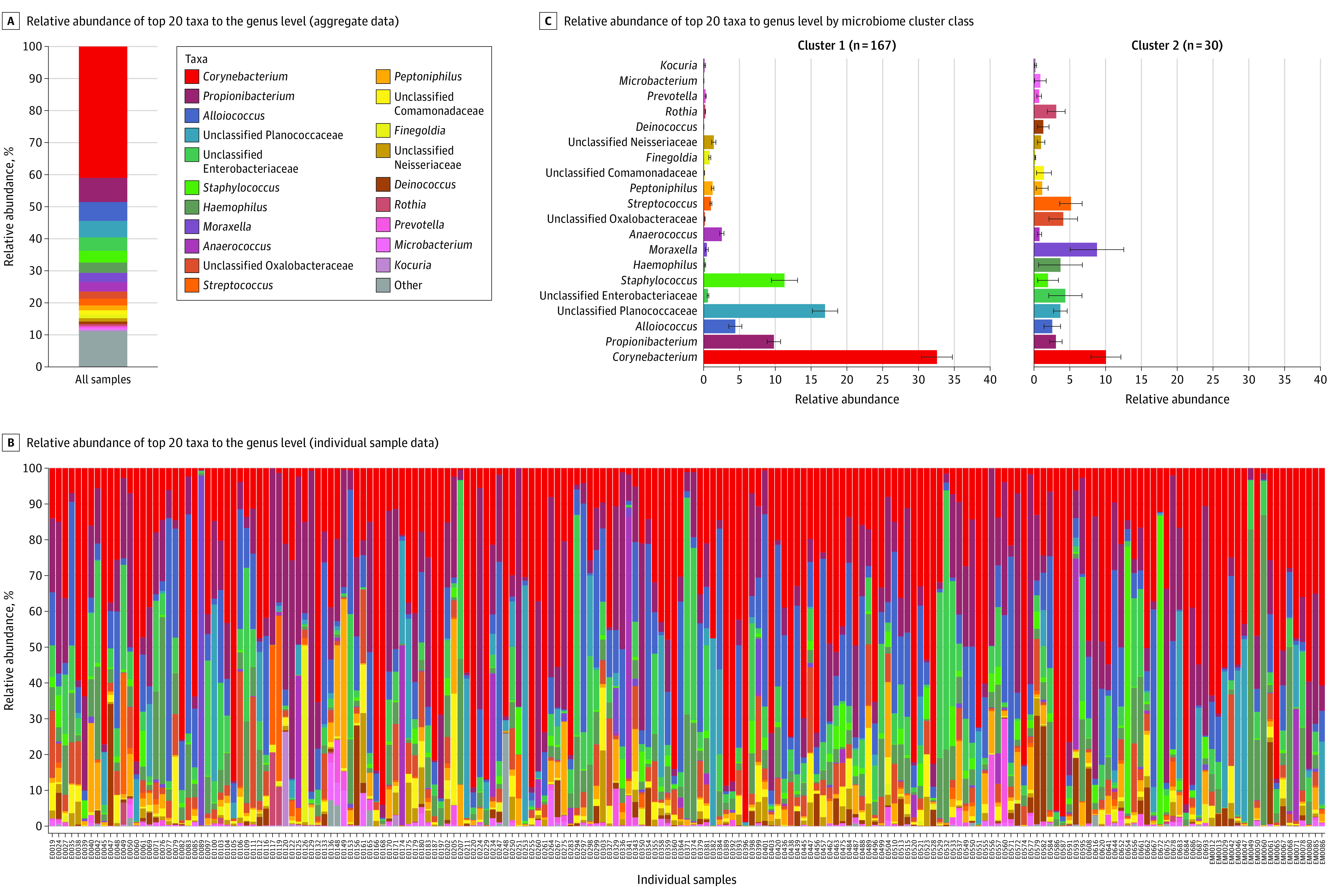
Relative Abundance of Nasal Microbiota Before Surgical Procedure C, Bars indicate mean; whiskers, SE.

Figure 1 part A shows the aggregate proportions of the top 20 most abundant taxa. *Corynebacterium* was the most abundant taxa detected from the anterior nares, constituting 41.0% of all sequences organized to the genus level, followed by *Propionibacterium* (7.6%), *Alloiococcus* (5.9%), *Planococcaceae* (5.1%), *Enterobacterales* (formerly *Enterobacteriaceae*) (4.2%), and *Staphylococcus* (3.7%). There was variability among study participants in proportions of the various taxa present in the anterior nares (Figure 1, part B). We detected 16S rRNA gene sequences for *S aureus* in 194 samples (98.5%). There was a positive association between *S aureus* relative abundance by 16S rRNA gene sequencing and nasal carriage of *S aureus* by standard clinical culture (odds ratio [OR], 1.93; 95% CI, 1.54-2.50; *q* < .0001). None of the other taxa, including other *Staphylococcal* species (ie, *epidermidis*,* pettenkoferi*, or *sciuri*) were associated with nasal carriage of *S aureus*. Pairwise comparisons of relative abundances of *S aureus* with other *Staphylococcal* species and with non-*Staphylococcal* taxa showed no significant associations after correcting for multiple testing.

Unsupervised clustering using grades of membership modeling, which was independent and agnostic of each participant’s clinical characteristics and infection status, classified participants into groups based solely on features derived from 16S rRNA gene sequencing of nasal microbiota. The clustering model with 2 groups (167 samples in cluster 1 and 30 samples in cluster 2) accounted for the greatest proportion of variance in the nasal microbiome and was most parsimonious (eFigure 2 in the Supplement), and it was thus selected for further characterization and hypothesis testing. Proportions of the top 20 most abundant genera differed between cluster 1 and cluster 2 (Figure 1, part C). Among the 553 distinct taxa identified to the species level, 67 taxa were significantly different between cluster 1 and cluster 2 (eTable 1 in the Supplement). Cluster 2 had greater α and β diversity than cluster 1 (eFigure 3 in the Supplement). Results from principal components analysis suggested that the factors that distinguished cluster 1 from cluster 2 were dispersed over a continuum rather than behaving as discrete categories (eFigure 3 in the Supplement).

Comparison of baseline clinical characteristics of the 197 study participants by the main exposure (ie, nasal microbiome cluster class) is shown in Table 1. There were no significant differences by microbial cluster class for any of the measured covariates, including demographic characteristics, comorbidities, surgical risk factors, and preoperative nasal carriage of *S aureus*. Of the 53 infections, 19 infections (35.8%) were SSI, 19 infections were bacteremia, and 27 infections (50.9%) were pneumonia (some participants experienced more than 1 infectious complication). *S aureus* was the most common organism to be isolated, at 23 (43.4%) infections, followed by *Klebsiella* species, at 11 infections (20.8%), and *Streptococcus* species, at 9 infections (17.0%); in some instances, more than 1 bacteria species was recovered from the infected site, and in others none were recovered (Table 2).

**Table 2.** Bacteria Isolated From Infected Individuals by Site of Infection

	No. (%)			
	Any infection (n = 53)	Deep SSI (n = 19)	Bacteremia (n = 19)	Pneumonia (n = 27)
*Staphylococcus epidermidis*	5 (9.4)	5 (26.3)	0	0
*Staphylococcus aureus*	23 (43.4)	9 (47.4)	6 (31.6)	8 (29.6)
*Streptococcus* species	9 (17.0)	3 (15.8)	2 (10.5)	4 (14.8)
*Pseudomonas* species	3 (5.7)	0	1 (5.3)	2 (7.4)
*Enterococcus* species	4 (7.6)	4 (21.1)	0	0
*Klebsiella* species	11 (20.8)	0	5 (26.3)	6 (22.2)
*Proteus* species	2 (3.8)	1 (5.3)	1 (5.3)	0
*Enterobacter* species	5 (9.4)	2 (10.5)	0	3 (11.1)
*Bacteroides* species	1 (1.9)	1 (5.3)	0	0
*Escherichia coli*	6 (11.3)	1 (5.3)	3 (15.8)	2 (7.4)
*Haemophilus* species	1 (1.9)	0	0	1 (3.7)
*Serratia* species	4 (7.6)	0	0	4 (14.8)
*Morganella* species	1 (1.9)	0	0	1 (3.7)
No organism isolated	12 (22.6)	4 (21.1)	0	8 (29.6)

Abbreviation: SSI, surgical site infection.

**Figure 2.**
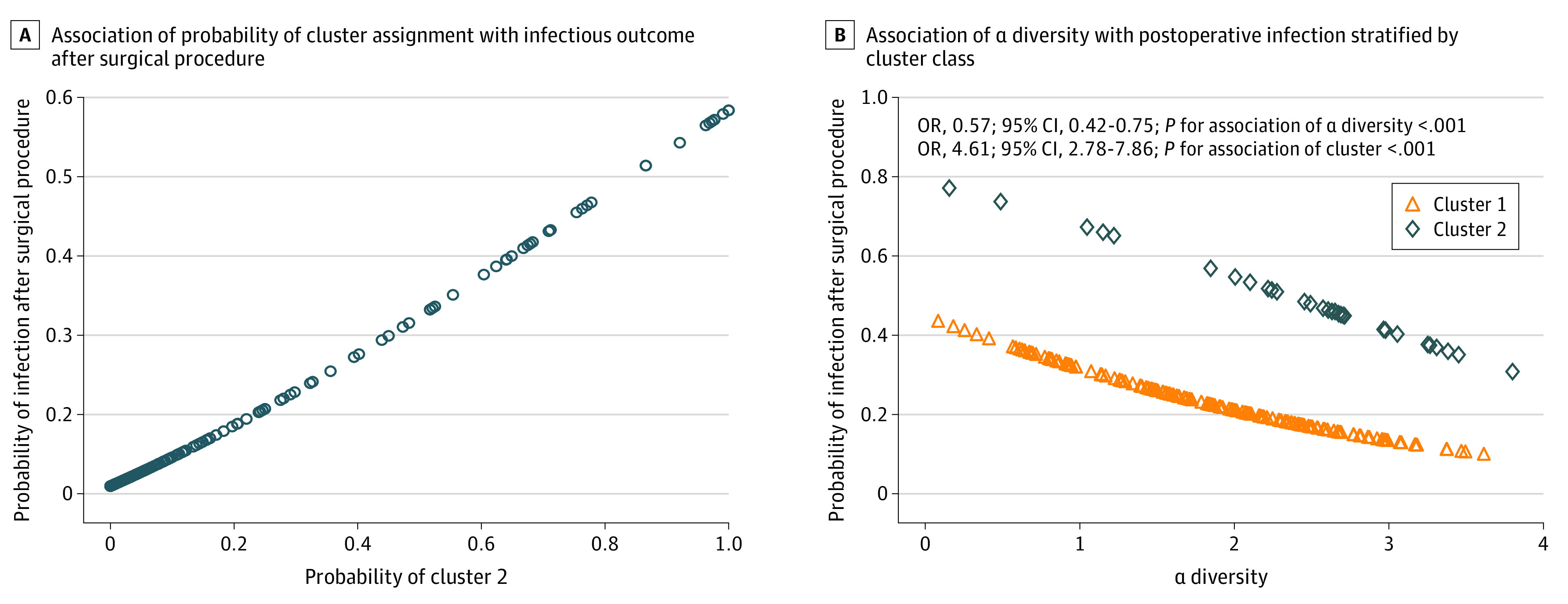
Association of Microbiome Cluster Class With Infectious Outcome After Surgical Procedure

The probability of assignment to cluster 2 was associated with an unadjusted 5-fold higher odds of composite postoperative infectious outcome (OR, 5.41; 95% CI, 1.81-16.54; *P* = .002) (Table 3). There was a dose-response association between probability of assignment to cluster 2 and infectious outcome (Figure 2). Similarly, categorical assignment to cluster 2 was associated with higher odds of infection (OR, 2.87; 95% CI, 1.27- 6.42; *P* = .009) compared with assignment to cluster 1.

**Table 3.** Association of Microbiome Cluster Class and Covariates With Composite Infectious Outcome^a^

Variable	Unadjusted		Model 1^b^		Model 2^c^		Model 3^d^		Model 4^e^		Model 5^f^	
	OR (95% CI)	*P* value	OR (95% CI)	*P* value	OR (95% CI)	*P* value	OR (95% CI)	*P* value	OR (95% CI)	*P* value	OR (95% CI)	*P* value
Cluster 2 probability	5.41 (1.81-16.54)	.002	5.73 (1.90-17.74)	.002	5.47 (1.63-19.00)	.006	5.56 (1.75-18.23)	.003	6.08 (1.92-19.72)	.002	6.18 (3.33-11.70)	<.001
Age	1.01 (0.97-1.03)	.60	1.01 (0.97-1.04)	.52	NA	NA	NA	NA	NA	NA	NA	NA
Women	0.85 (0.43-1.64)	.64	0.95 (0.47-1.88)	.90	NA	NA	NA	NA	NA	NA	NA	NA
Race												
White	1 [Reference]		1 [Reference]	NA	NA	NA	NA	NA	NA	NA	NA	NA
Black	0.72 (0.25-1.79)	.51	0.76 (0.26-1.96)	.99	NA	NA	NA	NA	NA	NA	NA	NA
Congestive heart failure	0.44 (0.10-1.40)	.21	NA	NA	0.10 (0.03-0.69)	.02	NA	NA	NA	NA	NA	NA
Peripheral vascular disease	1.53 (0.54-3.98)	.39	NA	NA	1.11 (0.34-3.34)	.85	NA	NA	NA	NA	NA	NA
COPD	2.93 (0.87-9.81)	.07	NA	NA	3.05 (0.81-11.60)	.09	NA	NA	NA	NA	NA	NA
Smoking	2.23 (1.11-4.78)	.21	NA	NA	2.04 (0.92-4.77)	.08	NA	NA	NA	NA	NA	NA
Cancer	2.63 (1.08-6.34)	.02	NA	NA	1.59 (0.53-4.74)	.40	NA	NA	NA	NA	NA	NA
Charlson Comorbidity Index score												
0-2	1 [Reference]	NA	NA	NA	1 [Reference]	NA	NA	NA	NA	NA	NA	NA
3-4	1.65 (0.71-3.94)	.24	NA	NA	1.58 (0.64-3.94)	.32	NA	NA	NA	NA	NA	NA
>5	3.57 (1.64-8.15)	.001	NA	NA	3.49 (1.34-9.39)	.01	NA	NA	NA	NA	NA	NA
Study site												
Johns Hopkins Hospital	1 [Reference]	NA	NA	NA	NA	NA	1 [Reference]	NA	NA	NA	NA	NA
University of Maryland Medical Center	0.51 (0.11-1.64)	.31	NA	NA	NA	NA	0.75 (0.15-2.74)	.69	NA	NA	NA	NA
Inpatient at the time of surgical procedure	2.03 (1.01-4.04)	.04	NA	NA	NA	NA	2.17 (0.92-5.20)	.08	NA	NA	NA	NA
Surgical procedure												
Cardiac	1 [Reference]		NA	NA	NA	NA	1 [Reference]	NA	NA	NA	NA	NA
Vascular	1.10 (0.38-2.96)	.83	NA	NA	NA	NA	0.96 (0.31-2.76)	.95	NA	NA	NA	NA
Spinal	0.44 (0.18-1.02)	.06	NA	NA	NA	NA	0.56 (0.20-1.48)	.25	NA	NA	NA	NA
Intracranial	2.06 (0.85-4.95)	.10	NA	NA	NA	NA	2.98 (1.07-8.40)	.04	NA	NA	NA	NA
Nasal culture for *S aureus*												
*S aureus* positive	2.70 (1.30-5.57)	.006	NA	NA	NA	NA	NA	NA	2.47 (1.05-5.68)	.03	NA	NA
Methicillin-resistant *S aureus* positive	6.00 (1.52-29.31)	.01	NA	NA	NA	NA	NA	NA	2.69 (0.55-15.30)	.220	NA	NA

Abbreviations: COPD, chronic obstructive pulmonary disease; NA, not applicable; OR, odds ratio; *S aureus*, *Staphylococcus aureus*.

^a^ The association between nasal microbiome cluster class and the composite infectious outcome was assessed in a series of regression models, including an unadjusted model and models adjusted (ie, models 1-5) for potential confounding baseline covariates from Table 1.

^b^ Model 1 adjusted for demographic covariates: age, sex, and race.

^c^ Model 2 adjusted for baseline comorbidities: congestive heart failure, peripheral vascular disease, COPD, history of smoking, history of cancer, and Charlson Comorbidity Index score.

^d^ Model 3 adjusted for surgical risk factors: study site, inpatient or outpatient status, and surgical procedure.

^e^ Model 4 adjusted for nasal culture results for *S aureus* and for methicillin-resistant *S aureus*.

^f^ Model 5 adjusted for inverse probability of treatment weighting using propensity score for assignment to microbiome cluster 2. The propensity score for assignment to cluster 2 incorporated all baseline covariates listed in Table 1.

We sought to determine if the association between probability of assignment to cluster 2 and infectious outcome was independent of baseline clinical covariates in a series of adjusted regression models. After adjustment in regression models, the odds of infection remained 5-fold to 6-fold higher for participants in cluster 2, ranging from an OR of 5.47 (95% CI, 1.90-17.74; P = .002) for model 2 to an OR of 6.18 (95% CI, 3.33-11.70; P < .001) for model 5 (Table 3). These results suggest that none of the baseline covariates, including demographic characteristics, relevant comorbidities, surgical risk factors, or nasal carriage of *S aureus*, were confounding covariates for the association between probability of cluster 2 assignment and infectious outcome. In model 5 (Table 3), with simultaneous adjustment for all baseline covariates using IPTW of the propensity score for assignment to cluster 2, odds of infection remained 6-fold higher. In IPTW-adjusted analyses, probability of assignment to cluster 2 was also associated with statistically significantly higher odds of the secondary outcomes of SSI only (OR, 2.90; 95% CI, 1.14-7.62; *P* = .03), pneumonia only (OR, 5.22; 95% CI, 2.56-10.94; *P* < .001), and composite infection within 30 days of surgical procedure (OR, 6.64; 95% CI, 3.36-13.48; *P* < .001). Odds increases for bacteremia (OR, 1.47; 95% CI, 0.56-3.79) and death (OR, 1.49; 95% CI, 0.59-3.70) were not statistically significant.

In evaluations of adjusted analyses using bootstrapping of subsamples, the mean (range) number of participants in each subsample was 134 (122-144) individuals. Grades of membership classified a mean (SD) 118.3 (12.7) participants into a major cluster 1 and 16.0 (11.8) participants into a minor cluster 2 in each iteration. In IPTW-adjusted analyses, the probability of assignment to cluster 2 was associated with 8-fold higher odds of infection after surgical procedure (OR, 7.91; 95% CI, 3.61-19.29; *P* < .001); categorical assignment to cluster 2 was associated with 4-fold higher odds of infection (OR, 4.20; 95% CI, 2.28-8.51; *P* < .001).

Given the association of microbiome cluster class with infectious outcome, we sought to identify characteristics of cluster 2 that might account for its association with infectious outcome. We found that α diversity was inversely associated with infectious outcome in both cluster groups (OR, 0.57; 95% CI, 0.42-0.75; *P *for main association of α diversity < .001); however, this association was independent of the association between cluster 2 and outcome (OR, 4.61, 95% CI, 2.78-7.86; *P* < .001). At any given level of α diversity, odds of infection were higher for individuals in cluster 2 than those in cluster 1 (Figure 2). We also examined the association of the 553 species-level taxa identified by 16S rRNA gene sequencing with infectious outcome. We found that 43 taxa were significantly associated with the composite infectious outcome after adjusting for multiple comparisons (eTable 2 in the Supplement), and of these, 7 taxa were also significantly associated with cluster. However, none of these taxa were recovered from a clinical site of infection. In adjusted analysis that included cluster 2 as a covariate, *Moraxella* (OR, 1.16, 95% CI, 1.00-1.34; *P* = .04), *Novosphingobium* (OR, 1.13, 95% CI, 1.05-1.23; *P* = .001), *Anaerococcus* (OR, 0.43, 95% CI, 0.31-0.57; *P* < .001), and *Atopobium* (OR, 0.69, 95% CI, 0.53-0.89; *P* = .005) were independently associated with clinical infectious outcome; however, these genera were not associated with changes in the association of cluster 2 with infectious outcome (eTable 3 in the Supplement).

## Discussion

To our knowledge, this case-control study is the first report finding an association between preoperative nasal microbial profiles and development of postoperative infectious complications, an association that was independent of the well-known connection between nasal colonization with *S aureus* and clinical infection. We found that microbial features derived solely from 16S rRNA gene sequencing classified individuals into groups by risk associated with development of SSI and pneumonia after surgical procedure.

This study provides new detail regarding composition of nasal microbiota in a population receiving surgical procedures for whom preoperative culture of the nares is common clinical practice. Similar to previous reports, this study found variability in the composition of nasal microbiota within and between individuals.^1^ Taxa representing common skin commensals, including *Corynebacterium*, *Propionibacterium* (also known as *Cutibacterium*), and *Staphylococcus*, were found in relatively high abundance,^1,23,24^ and we found several additional taxa, including *Alloiococcus*, *Anaerococcus*, *Planococcaceae*, and *Enterobacteriaceae*, to be present at high abundance levels. Differences between our study and others may be due to differences in sequenced amplicons, sequencing methods, bioinformatics methods used to classify operational taxonomic units, and participant populations.

*Staphylococcus* accounted for 3.7% of all observed sequences, and the prevalence of *S aureus* in the nasal microbiome was greater using 16S rRNA gene sequencing (98.5%) than standard clinical culture (20.8%). Other studies have reported higher detection rates of *S aureus* from concurrent samples of the anterior nares when using sequencing approaches vs when using culture methods,^2,25^ reflecting the greater sensitivity of sequencing for microbial detection. We found a positive association between relative abundance of *S aureus* as detected by 16S rRNA gene sequencing and nasal colonization with *S aureus* by clinical culture; however, we found no associations with abundances of other *Staphylococcal* species or non-*Staphylococcal* genera.

In our unsupervised grades of membership approach^18,19^ to classify samples based on 16S rRNA gene sequencing results, assignment of samples to clusters was independent and agnostic of baseline clinical characteristics or infection status. In our analysis, we found that a 2-cluster model accounted for the largest portion of variance while being most parsimonious. The approach used with grades of membership differs from popular methods for clustering sample-level microbiome data, such as hierarchical clustering^26^ and partition around medoids.^27,28^ The grades of membership method is probabilistic and based on the idea that each sample can have partial membership in multiple clusters rather than being forced into categorical assignment. Principal component analyses were consistent with partial membership of samples within clusters, given that samples were dispersed across vectors as a continuum rather than as discrete groupings. We did not find an association between nasal microbiome cluster assignment and several preoperative demographic, clinical, or surgical covariates.

Other investigators have used clustering methods to classify microbial composition of samples obtained from the anterior nares and nasal sinuses. In Liu et al,^2^ microbiome cluster class was associated with abundance of *S aureus* in the anterior nares, and in Abreu et al,^29^ cluster class discriminated between individuals with and without a diagnosis of chronic sinusitis. In Lehtinen et al,^24^ nasal microbiome class at baseline was associated with subsequent viral load, host inflammatory response, and symptom severity after experimental challenge with rhinovirus.

A major novel finding from our study was a temporal, dose-response association between preoperative nasal microbiome cluster class and subsequent development of infection at non-nasal sites. This association was independent of all measured covariates, including nasal carriage of *S aureus*, and was robust to iterative subsampling and bootstrapping analyses. Importantly, the odds of infection associated with nasal microbiome class were as large as or larger than those associated with nasal carriage of *S aureus*.

We found an inverse and independent association between α diversity of the preoperative nasal microbiome and odds of infection after surgical procedure, which is consistent with the well-described association of decreased α diversity with adverse clinical outcomes in a variety of disease states.^3^ A 2020 study^4^ reported that decreased α diversity of gut microbiota was associated with higher risk of death during 2-year follow-up in an observational cohort of patients who underwent allogeneic hematopoietic cell transplantation. Our observations extend previous reports by finding an association between nasal microbiome cluster class and adverse outcome that is independent of α diversity.

To our surprise, there was virtually no concordance between the taxa distinguishing microbiome cluster 1 from cluster 2 and those that caused clinical infection. Several potentially pathogenic taxa, including *Moraxella*,^30^ *Novosphingobium* (also known as *Sphingomonas*),^31^ *Anaerococcus*, and *Atopobium*,^32^ were associated with cluster and postoperative infectious outcome; however, none of these accounted for the association between cluster class and infection. These findings suggest that the taxa that distinguished cluster 1 from cluster 2 are not in the direct causal pathway to infectious outcome.

The mechanisms underlying the association between nasal microbiome cluster class and postoperative infection remain unclear. A possibility is that the aggregate composition of nasal microbiota signifies a latent phenotype of the host that reflects its responsiveness to infectious challenge and susceptibility to clinical infection. This possibility is supported by prior work from Lehtinen et al^24^ that demonstrated an association between characteristics of nasal microbiota at baseline and severity of coryzal symptoms after exposure to rhinovirus. Susceptibility to infection could be associated with an immunologic state inherent to the host, to an interaction between the host and microbiota that modifies susceptibility to infection, or both.

### Limitations

This study has several limitations. Our sample size was relatively small and drawn from patients undergoing a select group of surgical procedures; thus, these results may not generalize to other surgical populations. The association we observed between nasal microbiome cluster class and postoperative infection could be confounded by unmeasured covariates. Although we bootstrapped random subsamples, we lacked an independent sample to replicate our results. Additionally, we could not identify an immunologic mechanism to account for the association between nasal microbiome cluster class and postoperative infectious outcomes. Further studies are needed to replicate our findings and to examine the immunologic basis for differences in microbial profiles between clusters and their association with infection after surgical procedure.

## Conclusions

These findings suggest that nasal microbiome cluster class may be a novel risk factor associated with infection after surgical procedure, with potential to improve preoperative risk stratification. The nasal microbiome may be a biomarker associated with infectious disease susceptibility beyond the niche of the anterior nares.

## Notes

Supplement. **eAppendix.** Supplemental Methods **eReferences** **eTable 1.** Association of Taxa Abundance With Microbiome Cluster Class **eTable 2.** Association of Taxa Abundance With Composite Infectious Outcome **eTable 3.** Association of Individual Taxa and Cluster Class With Composite Infectious Outcome **eFigure 1.** Taxonomic Classification of 16S rRNA Gene Sequences Identified From Preoperative Nasal Swabs **eFigure 2.** Violin Plots of Variance Associated With Microbiome Cluster **eFigure 3.** Diversity Characterization of Nasal Microbiota by Cluster Class
