# Supplementary material for: Nasal Microbiota and Infectious Complications After Elective Surgical Procedures
Source: JAMA Netw Open. 2021 Apr 29;4(4):e218386. doi: 10.1001/jamanetworkopen.2021.8386 (PMC8085724; doi:10.1001/jamanetworkopen.2021.8386)
Supplement: Supplement. — eAppendix. Supplemental Methods eReferences eTable 1. Association of Taxa Abundance With Microbiome Cluster Class eTable 2. Association of Taxa Abundance With Composite Infectious Outcome eTable 3. Association of Individual Taxa and Cluster Class With Composite Infectious Outcome eFigure 1. Taxonomic Classification of 16S rRNA Gene Sequences Identified From Preoperative Nasal Swabs eFigure 2. Violin Plots of Variance Associated With Microbiome Cluster eFigure 3. Diversity Characterization of Nasal Microbiota by Cluster Class [file jamanetwopen-e218386-s001.pdf]

Supplemental Online Content

Hsiao CJ, Paulson JN, Singh S, et al. Nasal microbiota and infectious complications after elective surgical procedures. *JAMA Netw Open*. 2021;4(4):e218386 doi:10.1001/jamanetworkopen.2021.8386

eAppendix. Supplemental Methods

eReferences

eTable 1. Association of Taxa Abundance With Microbiome Cluster Class

eTable 2. Association of Taxa Abundance With Composite Infectious Outcome

eTable 3. Association of Individual Taxa and Cluster Class With Composite Infectious Outcome

eFigure 1. Taxonomic Classification of 16S rRNA Gene Sequences Identified From Preoperative Nasal Swabs

eFigure 2. Violin Plots of Variance Associated With Microbiome Cluster

eFigure 3. Diversity Characterization of Nasal Microbiota by Cluster Class

This supplemental material has been provided by the authors to give readers additional information about their work.

© 2021 Hsiao CJ et al. *JAMA Network Open.*

eAppendix. Supplemental Methods

Study design, participants, and protocol

We conducted a nested matched case-control study to investigate the association between nasal microbiota and postoperative infectious complications using participants selected from a prospective cohort study of elective high risk clean surgical procedures. The protocol for subjects enrolled in the prospective surgical cohort study, which served as the parent to the current microbiome study, was previously published[1]. Of the 802 subjects enrolled into the cohort at the Johns Hopkins Hospital or the University of Maryland Medical Center (from 2/1/2007 to 9/30/2014), we identified 53 participants who developed the composite infectious outcome (deep SSI, bacteremia, or pneumonia) within 6 months of the index surgical procedure. We selected non-infected controls to match cases (~3:1) by age (+/- 5 years), sex, and surgical procedure; for some cases, only 2 matches were identified (yielding a total of 144 controls). We targeted a sample size of ~200 cases plus controls given the financial resources available to us to conduct the microbiome analyses. Cases and controls were selected from a prospective surgical cohort because of the high incidence of bacterial disease expression in the weeks immediately following surgery, allowing temporal inference to be drawn regarding characteristics of microbiota in an infection-free state (before surgery) and subsequent development of clinical infection after a high risk exposure (i.e. surgery).

Patients were eligible for inclusion in the original cohort if they were ≥40 years old and underwent any of the following elective clean surgical procedures: cardiac surgery (coronary artery bypass or valve), vascular surgery (aortic or lower extremity revascularization or open aneurysm repair), spinal fusion, or craniotomy. These surgical procedures were selected because of the relatively high risk for postoperative SSI and pneumonia and because they share a common pathologic basis for SSI, including the type of infecting pathogen, that is, predominantly skin and mucosal flora. Patients were excluded for any of the following: history of immunodeficiency syndrome, autoimmune disease, immunosuppressive medications in the past 6 months (oral corticosteroids <5 days was allowed if not current at the time of surgery), central venous catheter was inserted before surgery, any infection/antibiotic therapy at the time of surgery (excluding antibiotics for perioperative prophylaxis), or inability to obtain written informed consent. (None of the participants was intubated at baseline.)

Baseline information from all subjects was obtained from in-person interviews and medical chart review. The following data elements, which could potentially confound the relation between the main exposure (i.e. nasal microbiome cluster class) and primary outcome (i.e. composite of postoperative SSI, bacteremia, or pneumonia), were recorded: demographics (age, sex, race), baseline comorbidities, infection or hospitalization in the year prior to surgery, American Society of Anesthesiologists Class, Charlson Comorbidity score, inpatient/outpatient status at time of surgery, and study site (Johns Hopkins Hospital, University of Maryland Medical Center). A previous report in twins indicated that genetic factors contribute to composition of nasal microbiota[2]. Because genetic architecture is known to differ by race, we included race as a demographic variable to adjust for potential confounding between the main exposure (nasal microbiome cluster class) and infectious outcome. Race was self-reported by each participant. The following comorbidities were recorded: obesity (BMI >/=30 by weight and height recorded at time of surgery), diabetes, hypertension, myocardial infarction, congestive heart failure, peripheral vascular disease, cerebrovascular disease (previous stroke or TIA), chronic obstructive pulmonary disease (COPD), smoking history (past or present), gastric ulcer disease, chronic liver disease, renal failure requiring dialysis, history of cancer (past or in the context of the scheduled surgical procedure). Each comorbidity was dichotomized and recorded as present if endorsed by the patient during the baseline interview or if the comorbidity was

© 2021 Hsiao CJ et al. *JAMA Network Open.*

recorded as present in the hospital record admission history and physical. For the diagnoses of diabetes, hypertension, and COPD, the subject also required concurrent treatment with a therapeutic agent for that disease for the comorbidity to be recorded as present; i.e., oral hypoglycemic agent or insulin for diabetes, anti-hypertensive agent for hypertension, inhaled bronchodilator or inhaled steroid for COPD.

Identification of *S aureus* from nasal swabs by clinical culture
Both anterior nares were swabbed with culturettes at the baseline visit before surgery. Swabs were transported to the clinical laboratory and plated to Chromagar S. aureus (BD Diagnostics, Sparks, MD) then stored at -80C for subsequent extraction of bacterial DNA for 16S rRNA gene sequencing (see below). Plates were incubated in the dark at 37°C for a minimum of 20 hours and then reincubated for another 24 hours, if negative. All mauve colonies were confirmed as *S aureus* positive using latex agglutination (Staphaurex Plus; Remel, Lenexa, KS).

Main exposure
We classified each of the 197 participants in this case-control study based solely on features derived from 16S rRNA gene sequencing of the material extracted from each subject's preoperative nasal swab. Nasal microbiome cluster class served as the main exposure. Two critical steps- 16S rRNA gene sequencing followed by unsupervised clustering analysis- were taken to classify nasal microbiota samples independently and agnostic of the baseline covariates and infection case/control status of each participant. A detailed description of these two steps follows below:

Microbiota profiling using 16S rRNA gene sequencing
Total metagenomic DNA was extracted and isolated from nasal swabs as previously described[3,4]. Briefly, samples were thawed and spun down, the resulting cell pellet was resuspended in 1 ml ice-cold PBS and transferred into Lysing Matrix B tubes (MP Biomedicals, Solon, OH). Bacterial lysis was then performed using two rounds of enzymatic lysis, first with a cocktail composed of lysozyme, mutanolysin, proteinase K, and then with lysostaphin, followed by mechanical lysis using bead beating. The mgDNA was then further purified using the Zymo fecal DNA kit (Zymogen). Negative extraction controls (PBS) were processed in parallel with each extraction to ensure no contaminating DNA was introduced during the DNA extraction process and PCRs. All samples included in our analyses had negative controls.

Microbiota profiling was performed by PCR amplification of the V3V4 hypervariable region of the 16S rRNA gene, followed by sequencing on the Illumina MiSeq 300-bp paired-reads platform (Illumina, San Diego, CA) using procedures previously published[5,6]. Sample barcoding was performed using the dual-indexing strategy for multiplexed sequencing developed at the Institute for Genome Sciences[5,6]. PCRs were set-up in 96-well microtiter plates using the 319F and 806R 16S universal primers, each of which also included a linker sequence required for Illumina sequencing, and also a 12-bp heterogeneity spacer index sequence aimed at minimizing biases associated with low-diversity amplicon sequencing[5,6]. No-template negative controls were processed for each primer pair. The presence of PCR amplicons was confirmed using gel electrophoresis, after which the SequalPrep normalization plate kit (Life Technologies, Inc.) was used for cleanup and normalization before sequencing.

Following sequencing, initial processing of the raw 16S sequences (read pair merging, read trimming and chimera filtering) was performed as previously described[4]. We then used QIIME 1.9.1[7] to generate an open-reference operational taxonomic unit (OTU) matrix and to perform all downstream analyses. In the process, sequence reads were clustered using a 97% similarity cutoff with UCLUST version 1.2.22 and aligned using PyNAST v1.2.2 against a core

© 2021 Hsiao CJ et al. *JAMA Network Open.*

Greengenes database vgg_13_8[8]. OTUs were assigned a taxonomic annotation using the RDP Classifier v2.0.2[9] and labeled to the most specific known taxonomic level. When taxonomic classification was not possible for a given taxa level, OTUs were labeled as “unclassified”.

Clustering analysis of nasal microbial profiles
CountClust[10] (R package) was used to assign each sample to a clustering group based on the counts for each taxonomic unit in each sample. CountClust fits a topic mixed membership model to visualize and annotate clusters, extracting the top features driving cluster membership. Grades of membership modeling (GoM) was used to determine the number of clusters that provided best fit to the data. GoM allows each sample to have some proportion of its membership – partial membership – in each cluster. For microbiome data, the partial membership assumption corresponds to each sample having some proportion of its reads from each cluster, and each cluster is characterized by a probability vector of relative taxa expression levels. In our analysis, we used these partial membership weights to assign each sample to a cluster. To determine the optimal number of clusters, we computed log Bayes factor (provided in CountClust) and compared model fit under k=2 to 5 versus a null single cluster model. The log Bayes factor was corrected for the number of parameters fitted in each model. We evaluated the data with k = 2 to 5 and found that k=2 produced the largest Bayes factor. Because sample cluster assignment may differ between different random seeds, despite similar cluster characteristics in feature abundance, we ran CountClust under 100 different random seeds. We chose the random seed that produced the largest Bayes Factors across different random seeds and sample cluster membership.

Primary and secondary outcomes
The primary outcome was a composite of deep SSI, pneumonia, or bacteremia, as defined by CDC surveillance criteria[11], occurring within 6 months postoperatively. Bacteremia was defined as the occurrence of one or more positive blood cultures for a pathogenic organism or 2 or more positive cultures at different times for a skin commensal. Secondary outcomes were SSI, pneumonia, and bacteremia separately, the composite outcome at 30 days, and death at 6 months.

Outcomes were ascertained during the 6 month follow-up period after surgery for all participants involved in the original cohort. Follow-up data were obtained from each participant by study team personnel using in-person or phone interview and by review of medical records. Follow-up occurred daily during the inpatient phase of care immediately following the index surgical procedure and then at 30 days and 6 months after the date of surgery. Outpatient follow-up consisted of scripted mail and/or phone surveys. In addition, medical records were obtained from any primary or specialty care visits and for emergency department encounters and hospitalizations that occurred after discharge from the index surgical procedure. All outcomes reported by patients were confirmed from the obtained medical records, and in cases of death, from death certificates and/or autopsy reports. The social security death index was queried for loses to follow-up.

The outcomes for participants in this nested matched case-control study, like all outcomes in the original cohort, were adjudicated by trained research nurses.

Statistical analyses
Aggregated counts of rRNA sequences to the species level annotation were batch corrected and used for differential abundance analysis. To account for the variability in sequencing depth we performed cumulative sum scaling (CSS)[12]. To identify taxa significantly different between

© 2021 Hsiao CJ et al. *JAMA Network Open.*

clusters and accounting for the sparsity inherent to 16S rRNA gene sequencing, we performed differential abundance analysis using a zero-inflated log-normal model as implemented within metagenomeSeq version 1.21.1 9 (software publically available at 10.18129/B9.bioc.metagenomeSeq)[13]. To characterize the within-sample (alpha) diversity, we calculated Shannon's Diversity Index (H). To characterize the between-sample (beta) diversity, we performed Principal Component Analysis on CSS $\log_2$ normalized species abundances.

Generalized linear model (GLM) analyses were performed to determine the factors associated with primary and secondary outcomes, unadjusted and adjusted for potentially cofounding covariates. Weighted probability of assignment to microbiome cluster class served as the main exposure. Models were defined using a binomial probability distribution and a logit link function. In a first step, demographic and comorbidities risk factors were tested individually between clusters. In a second step, a propensity score (PS) for cluster assignment was estimated. To estimate the PS, we used multivariable logistic regression analysis that included all of the baseline variables. Inverse probability of treatment weights (IPTW) used the PS to form a weight. The IPTW approach used weights based on the propensity score to create a sample in which the distribution of measured baseline covariates was independent of cluster assignment. The weights were incorporated in the multivariable weighted logistic regression model to predict infectious outcome.

We bootstrapped propensity score analyses by selecting ~70% of the 197 subjects in 500 subsamples, maintaining a fixed ratio of cases to controls in the subsamples (1:2.8). In each subsample, we first selected the case subjects with matched control subjects. We randomly sampled 38 cases in each subsample with replacement and then iteratively sampled matched control subjects for each case subject until the ratio of case vs control subject satisfied our requirement. The number of subjects in each of the 500 subsamples ranged between 122 to144.

We examined the association of infection with alpha diversity and abundance of individual microbial taxa using logistic regression. Due to skewness in the abundance (count) of microbial taxa, we log transformed counts to approximate normality. Analyses were performed using the R version 3.2.2 (R foundation for Statistical Computing, Vienna, Austria). Statistical significance was set at p-value < 0.05 and all tests were two-sided. Benjamini-Hochberg false discovery procedure was used to correct for multiple comparisons.

Data access
Github site which contains all scripts to reproduce our analysis.
https://jhsiao999.github.io/nasalmicrobiome/

© 2021 Hsiao CJ et al. *JAMA Network Open.*

eReferences

1. Faraday N, Rock P, Lin EE, et al. Past History of Skin Infection and Risk of Surgical Site Infection after Elective Surgery. *Ann. Surg.* May 24 2012.
2. Liu CM, Price LB, Hungate BA, et al. Staphylococcus aureus and the ecology of the nasal microbiome. *Science advances.* Jun 2015;1(5):e1400216.
3. Zupancic ML, Cantarel BL, Liu Z, et al. Analysis of the gut microbiota in the old order Amish and its relation to the metabolic syndrome. *PLoS One.* 2012;7(8):e43052.
4. Roghmann MC, Lydecker AD, Hittle L, et al. Comparison of the Microbiota of Older Adults Living in Nursing Homes and the Community. *mSphere.* Sep-Oct 2017;2(5).
5. Fadrosh DW, Ma B, Gajer P, et al. An improved dual-indexing approach for multiplexed 16S rRNA gene sequencing on the Illumina MiSeq platform. *Microbiome.* Feb 24 2014;2(1):6.
6. Holm JB, Humphrys MS, Robinson CK, et al. Ultrahigh-Throughput Multiplexing and Sequencing of >500-Base-Pair Amplicon Regions on the Illumina HiSeq 2500 Platform. *mSystems.* Jan-Feb 2019;4(1).
7. Caporaso JG, Kuczynski J, Stombaugh J, et al. QIIME allows analysis of high-throughput community sequencing data. *Nature methods.* May 2010;7(5):335-336.
8. DeSantis TZ, Hugenholtz P, Larsen N, et al. Greengenes, a chimera-checked 16S rRNA gene database and workbench compatible with ARB. *Appl. Environ. Microbiol.* Jul 2006;72(7):5069-5072.
9. Wang Q, Garrity GM, Tiedje JM, Cole JR. Naive Bayesian classifier for rapid assignment of rRNA sequences into the new bacterial taxonomy. *Appl. Environ. Microbiol.* Aug 2007;73(16):5261-5267.
10. Dey KK, Hsiao CJ, Stephens M. Visualizing the structure of RNA-seq expression data using grade of membership models. *PLoS genetics.* Mar 2017;13(3):e1006599.
11. A Report from the NNIS System. National Nosocomial Infections Surveillance (NNIS) System Report, data summary from January 1992 through June 2004, issued October 2004. *Am. J. Infect. Control.* 2004;32:470-485.
12. Paulson JN, Stine OC, Bravo HC, Pop M. Differential abundance analysis for microbial marker-gene surveys. *Nature methods.* Dec 2013;10(12):1200-1202.
13. Paulson JN, Olson ND, Braccia DJ, et al. Statistical analysis for sparse high-throughput sequencing. *Biocoductor 3.10.* 2013;DOI: 10.18129/B9.bioc.metagenomeSeq

© 2021 Hsiao CJ et al. *JAMA Network Open.*

eTable 1. Association of Taxa Abundance With Microbiome Cluster Class

Taxa	samples clust1	samples clust2	counts clust1	counts clust2	logFC	se	p-value	q-value
k__Bacteria;p__Actinobacteria;c__Actinobacteria;o__Actinomycetales;f__Mycobacteriaceae;g__Mycobacterium;s__	28	11	76	7171	3.631E+00	0.6016	1.580E-09	5.310E-07
k__Bacteria;p__Proteobacteria;c__Alphaproteobacteria;o__Rhizobiales;f__;g__;s__	154	30	1808	13825	1.609E+00	0.2868	2.020E-08	3.390E-06
k__Bacteria;p__Chloroflexi;c__Thermomicrobia;o__JG30-KF-CM45;f__;g__;s__	11	9	22	3152	4.098E+00	0.7514	4.950E-08	4.140E-06
k__Bacteria;p__TM7;c__TM7-1;o__;f__;g__;s__	19	10	46	6605	3.603E+00	0.6587	4.510E-08	4.140E-06
k__Bacteria;p__Actinobacteria;c__Actinobacteria;o__Actinomycetales;f__;g__;s__	27	15	84	12099	3.057E+00	0.5651	6.330E-08	4.240E-06
k__Bacteria;p__Actinobacteria;c__Thermoleophilia;o__Solirubrobacterales;f__;g__;s__	11	12	19	2749	3.806E+00	0.7125	9.220E-08	4.550E-06
k__Bacteria;p__Proteobacteria;c__Betaproteobacteria;o__Burkholderiales;f__Oxalobacteraceae;g__;s__	139	29	2924	102400	1.756E+00	0.3292	9.520E-08	4.550E-06
k__Bacteria;p__Proteobacteria;c__Betaproteobacteria;o__Burkholderiales;f__Comamonadaceae;g__;s__	128	29	2737	60889	1.508E+00	0.3139	1.570E-06	6.550E-05
k__Bacteria;p__Actinobacteria;c__Actinobacteria;o__Actinomycetales;f__Microbacteriaceae;g__;s__	44	12	214	8241	2.668E+00	0.5643	2.270E-06	8.440E-05
k__Bacteria;p__Actinobacteria;c__Acidimicrobiia;o__Acidimicrobiales;f__;g__;s__	12	10	17	1134	3.266E+00	0.7003	3.100E-06	1.037E-04
k__Bacteria;p__Firmicutes;c__Clostridia;o__Clostridiales;f__Clostridiaceae;g__SMB53;s__	19	11	87	5762	2.969E+00	0.6553	5.890E-06	1.793E-04
k__Bacteria;p__Firmicutes;c__Clostridia;o__Clostridiales;f__Clostridiaceae;g__;s__	16	9	47	2918	3.099E+00	0.6901	7.090E-06	1.979E-04
k__Bacteria;p__Proteobacteria;c__Alphaproteobacteria;o__Rhodobacterales;f__Rhodobacteraceae;g__;s__	23	16	285	1648	2.332E+00	0.5398	1.560E-05	4.021E-04
k__Bacteria;p__TM7;c__TM7-3;o__;f__;g__;s__	49	20	951	2999	1.832E+00	0.4285	1.920E-05	4.584E-04
k__Bacteria;p__Firmicutes;c__Bacilli;o__Bacillales;f__[Exiguobacteraceae];g__Exiguobacterium;s__	41	14	137	2999	2.024E+00	0.4865	3.180E-05	7.104E-04
k__Bacteria;p__Firmicutes;c__Clostridia;o__Clostridiales;f__[Tissierellaceae];g__Parvimonas;s__	28	8	211	751	2.589E+00	0.6532	7.410	1.551

© 2021 Hsiao CJ et al. *JAMA Network Open.*

							E-05	E-03
**k__Bacteria;p__Proteobacteria;c__Gammaproteobacteria;o__Legionellales;f__Legionellaceae;g__;s__**	7	8	8	1892	3.509E+00	0.9033	1.027E-04	1.911E-03
**k__Bacteria;p__Actinobacteria;c__Actinobacteria;o__Actinomycetales;f__Propionibacteriaceae;g__Propionibacterium;s__**	153	21	14734	662	-1.646E+00	0.4236	1.022E-04	1.911E-03
**k__Bacteria;p__Proteobacteria;c__Gammaproteobacteria;o__Pseudomonadales;f__Moraxellaceae;g__Moraxella;s__**	46	12	17486	121207	3.354E+00	0.8735	1.235E-04	2.177E-03
**k__Bacteria;p__Bacteroidetes;c__Sphingobacteriia;o__Sphingobacteriales;f__Sphingobacteriaceae;g__Sphingobacterium;s__**	6	8	11	110	3.258E+00	0.8664	1.697E-04	2.843E-03
**k__Bacteria;p__Cyanobacteria;c__Synechococcophycideae;o__Synechococcales;f__Synechococcaceae;g__Synechococcus;s__**	12	2	26	1593	4.373E+00	1.1851	2.248E-04	3.274E-03
**k__Bacteria;p__[Thermi];c__Deinococci;o__Deinococcales;f__Deinococcaceae;g__Deinococcus;s__**	27	13	401	37448	2.549E+00	0.6880	2.112E-04	3.274E-03
**k__Bacteria;p__Actinobacteria;c__Actinobacteria;o__Actinomycetales;f__Microbacteriaceae;g__Microbacterium;s__**	55	18	343	25345	1.697E+00	0.4596	2.221E-04	3.274E-03
**k__Bacteria;p__Proteobacteria;c__Betaproteobacteria;o__Methylophilales;f__Methylophilaceae;g__;s__**	4	5	4	606	3.794E+00	1.0598	3.434E-04	4.793E-03
**k__Bacteria;p__Firmicutes;c__Clostridia;o__Clostridiales;f__Lachnospiraceae;g__;s__**	59	20	630	1329	1.512E+00	0.4244	3.659E-04	4.903E-03
**k__Bacteria;p__Acidobacteria;c__[Chloracidobacteria];o__RB41;f__Ellin6075;g__;s__**	17	9	151	1918	2.437E+00	0.6860	3.818E-04	4.919E-03
**k__Bacteria;p__Firmicutes;c__Bacilli;o__Lactobacillales;f__Carnobacteriaceae;g__Granulicatella;s__**	86	16	2677	14065	1.759E+00	0.5001	4.363E-04	5.413E-03
**k__Bacteria;p__TM7;c__SC3;o__;f__;g__;s__**	10	7	21	3266	3.192E+00	0.9148	4.852E-04	5.459E-03
**k__Bacteria;p__Proteobacteria;c__Alphaproteobacteria;o__Rickettsiales;f__;g__;s__**	9	6	30	1105	3.100E+00	0.8844	4.574E-04	5.459E-03
**k__Bacteria;p__Actinobacteria;c__Actinobacteria;o__Actinomycetales;f__Actinomycetaceae;g__Actinomyces;s__**	111	24	3395	5006	1.310E+00	0.3756	4.889E-04	5.459E-03
**k__Bacteria;p__Firmicutes;c__Clostridia;o__Clostridiales;f__Peptostreptococcaceae;g__Peptostreptococcus;s__**	15	6	107	492	2.758E+00	0.7981	5.492E-04	5.575E-03
**k__Bacteria;p__Proteobacteria;c__Alphaproteobacteria;o__Sphingomonadales;f__;g__;s__**	12	9	34	872	2.489E+00	0.7201	5.463E-04	5.575E-03
**k__Bacteria;p__Proteobacteria;c__Alphaproteobacteria;o__Sphingomonadales;f__Sphingomonadaceae;g__Novosphingobium;s__**	13	11	41	260	2.214E+00	0.6386	5.265	5.575

© 2021 Hsiao CJ et al. *JAMA Network Open.*

							E-04	E-03
**k__Bacteria;p__Firmicutes;c__Bacilli;o__Lactobacillales;f__Streptococcaceae;g__Streptococcus;s__**	161	30	36652	62811	1.137E+00	0.3298	5.681E-04	5.597E-03
**k__Bacteria;p__Proteobacteria;c__Alphaproteobacteria;o__Sphingomonadales;f__Sphingomonadaceae;g__;s__**	34	13	157	2040	1.749E+00	0.5109	6.178E-04	5.914E-03
**k__Bacteria;p__Actinobacteria;c__Actinobacteria;o__Bifidobacteriales;f__Bifidobacteriaceae;g__Bifidobacterium;s__longum**	13	11	65	836	2.443E+00	0.7192	6.822E-04	6.349E-03
**k__Bacteria;p__Proteobacteria;c__Alphaproteobacteria;o__Rhizobiales;f__Hyphomicrobiaceae;g__Hyphomicrobium;s__**	7	10	25	1637	2.930E+00	0.8723	7.825E-04	7.085E-03
**k__Bacteria;p__Proteobacteria;c__Alphaproteobacteria;o__Rhodobacterales;f__Rhodobacteraceae;g__Paracoccus;s__marcusii**	32	13	85	1483	1.575E+00	0.4708	8.227E-04	7.253E-03
**k__Bacteria;p__Bacteroidetes;c__Cytophagia;o__Cytophagales;f__Cytophagaceae;g__Hymenobacter;s__**	23	11	122	15245	2.284E+00	0.6942	1.001E-03	8.385E-03
**k__Bacteria;p__Actinobacteria;c__Coriobacteriia;o__Coriobacteriales;f__Coriobacteriaceae;g__Atopobium;s__**	37	9	204	401	1.813E+00	0.5505	9.914E-04	8.385E-03
**k__Bacteria;p__Proteobacteria;c__Alphaproteobacteria;o__Rhizobiales;f__Methylocystaceae;g__Methylosinus;s__**	5	8	5	252	2.928E+00	0.8954	1.077E-03	8.803E-03
**k__Bacteria;p__Proteobacteria;c__Alphaproteobacteria;o__Rhizobiales;f__Beijerinckiaceae;g__;s__**	17	10	68	3389	2.319E+00	0.7123	1.131E-03	9.024E-03
**k__Bacteria;p__Proteobacteria;c__Betaproteobacteria;o__Burkholderiales;f__Alcaligenaceae;g__;s__**	25	10	166	1177	2.153E+00	0.6630	1.164E-03	9.070E-03
**k__Bacteria;p__Actinobacteria;c__Acidimicrobiia;o__Acidimicrobiales;f__EB1017;g__;s__**	3	9	3	172	3.320E+00	1.0418	1.439E-03	1.095E-02
**k__Bacteria;p__Proteobacteria;c__Alphaproteobacteria;o__Rhizobiales;f__Hyphomicrobiaceae;g__Rhodoplanes;s__**	12	10	21	259	2.004E+00	0.6317	1.515E-03	1.103E-02
**k__Bacteria;p__Actinobacteria;c__Actinobacteria;o__Actinomycetales;f__Nocardioidaceae;g__;s__**	35	13	288	683	1.606E+00	0.5061	1.506E-03	1.103E-02
**k__Bacteria;p__Actinobacteria;c__Acidimicrobiia;o__Acidimicrobiales;f__C111;g__;s__**	9	8	35	749	2.669E+00	0.8510	1.712E-03	1.220E-02
**k__Bacteria;p__Firmicutes;c__Clostridia;o__Clostridiales;f__Veillonellaceae;g__Veillonella;s__dispar**	114	19	7907	9307	1.299E+00	0.4178	1.878E-03	1.258E-02
**k__Bacteria;p__Proteobacteria;c__Alphaproteobacteria;o__Rhizobiales;f__Bradyrhizobiaceae;g__;s__**	111	27	1318	969	8.217E-01	0.2637	1.836E-03	1.258E-02
**k__Bacteria;p__Actinobacteria;c__Actinobacteria;o__Actinomycetales;f__Micrococcaceae;g__Micrococcus;s__**	145	27	466	37	-6.42	0.2064	1.873	1.258

© 2021 Hsiao CJ et al. *JAMA Network Open.*

					0E-01		E-03	E-02
**k__Bacteria;p__Actinobacteria;c__Actinobacteria;o__Actinomycetales;f__Propionibacteriaceae;g__Propionibacterium;s__granulosum**	154	27	922	73	-6.364E-01	0.2057	1.972E-03	1.296E-02
**k__Bacteria;p__Proteobacteria;c__Gammaproteobacteria;o__Pseudomonadales;f__Moraxellaceae;g__;s__**	67	17	629	16591	1.438E+00	0.4698	2.203E-03	1.420E-02
**k__Bacteria;p__Actinobacteria;c__Acidimicrobiia;o__Acidimicrobiales;f__Microthrixaceae;g__;s__**	3	8	3	219	3.354E+00	1.1142	2.607E-03	1.648E-02
**k__Bacteria;p__Firmicutes;c__Bacilli;o__Bacillales;f__Bacillaceae;g__Anoxybacillus;s__kestanbolensis**	147	27	2201	108	-9.320E-01	0.3104	2.677E-03	1.661E-02
**k__Bacteria;p__Proteobacteria;c__Gammaproteobacteria;o__Xanthomonadales;f__Xanthomonadaceae;g__Stenotrophomonas;s__**	145	26	588	47	-6.092E-01	0.2122	4.088E-03	2.490E-02
**k__Bacteria;p__Firmicutes;c__Clostridia;o__Clostridiales;f__[Tissierellaceae];g__Anaerococcus;s__**	159	27	128879	9195	-1.169E+00	0.4086	4.222E-03	2.495E-02
**k__Bacteria;p__Proteobacteria;c__Alphaproteobacteria;o__Sphingomonadales;f__Sphingomonadaceae;g__Sphingomonas;s__**	72	19	1131	9483	1.159E+00	0.4052	4.246E-03	2.495E-02
**k__Bacteria;p__Firmicutes;c__Bacilli;o__Bacillales;f__;g__;s__**	66	4	1648	29	-2.695E+00	0.9457	4.368E-03	2.514E-02
**k__Bacteria;p__Proteobacteria;c__Alphaproteobacteria;o__Rhodospirillales;f__Acetobacteraceae;g__;s__**	26	10	94	393	1.679E+00	0.5899	4.429E-03	2.514E-02
**k__Bacteria;p__Firmicutes;c__Bacilli;o__Bacillales;f__Planococcaceae;g__Paenisporosarcina;s__**	25	8	79	2314	1.983E+00	0.6992	4.575E-03	2.554E-02
**k__Bacteria;p__Cyanobacteria;c__Chloroplast;o__Chlorophyta;f__Trebouxiophyceae;g__;s__**	8	6	28	16300	3.440E+00	1.2225	4.897E-03	2.689E-02
**k__Bacteria;p__Synergistetes;c__Synergistia;o__Synergistales;f__Dethiosulfovibrionaceae;g__TG5;s__**	4	3	26	176	3.627E+00	1.3003	5.285E-03	2.856E-02
**k__Bacteria;p__Proteobacteria;c__Betaproteobacteria;o__Rhodocyclales;f__Rhodocyclaceae;g__;s__**	17	9	32	388	1.872E+00	0.6781	5.776E-03	3.043E-02
**k__Bacteria;p__Proteobacteria;c__Alphaproteobacteria;o__Rhodospirillales;f__Rhodospirillaceae;g__;s__**	31	14	199	777	1.358E+00	0.4924	5.814E-03	3.043E-02
**k__Bacteria;p__Actinobacteria;c__Actinobacteria;o__Actinomycetales;f__Microbacteriaceae;g__CandidatusAquiluna;s__rubra**	4	4	9	2574	3.823E+00	1.3900	5.959E-03	3.071E-02
**k__Bacteria;p__Fusobacteria;c__Fusobacteriia;o__Fusobacteriales;f__Fusobacteriaceae;g__Fusobacterium;s__**	74	15	2241	1500	1.257E+00	0.4723	7.790E-03	3.954E-02
**k__Bacteria;p__Chlamydiae;c__Chlamydiia;o__Chlamydiales;f__;g__;s__**	3	6	3	648	3.414E+00	1.3084	9.062	4.531

© 2021 Hsiao CJ et al. *JAMA Network Open.*

							E-03	E-02
**k__Bacteria;p__Actinobacteria;c__Actinobacteria;o__Actinomycetales;f__Intrasporangiaceae;g__;s__**	38	8	387	5313	1.801E+00	0.7041	1.054E-02	5.191E-02
**k__Bacteria;p__Bacteroidetes;c__Flavobacteriia;o__Flavobacteriales;f__Flavobacteriaceae;g__Flavobacterium;s__**	46	14	482	2096	1.331E+00	0.5282	1.174E-02	5.701E-02
**k__Bacteria;p__Proteobacteria;c__TA18;o__PHOS-HD29;f__;g__;s__**	2	7	2	112	3.215E+00	1.2860	1.243E-02	5.866E-02
**k__Bacteria;p__Proteobacteria;c__Alphaproteobacteria;o__Sphingomonadales;f__Sphingomonadaceae;g__Sphingopyxis;s__**	4	9	8	470	2.681E+00	1.0713	1.231E-02	5.866E-02
**k__Bacteria;p__Proteobacteria;c__Gammaproteobacteria;o__Aeromonadales;f__Aeromonadaceae;g__;s__**	33	8	191	1067	1.647E+00	0.6655	1.333E-02	6.203E-02
**k__Bacteria;p__Firmicutes;c__Clostridia;o__Clostridiales;f__Lachnospiraceae;g__Moryella;s__**	24	7	283	2262	1.974E+00	0.8009	1.370E-02	6.287E-02
**k__Bacteria;p__TM6;c__SJA-4;o__;f__;g__;s__**	2	6	2	1458	3.891E+00	1.5907	1.444E-02	6.521E-02
**k__Bacteria;p__TM7;c__;o__;f__;g__;s__**	6	7	16	2793	2.609E+00	1.0727	1.499E-02	6.521E-02
**k__Bacteria;p__Firmicutes;c__Erysipelotrichi;o__Erysipelotrichales;f__Erysipelotrichaceae;g__Bulleidia;s__**	8	7	53	755	2.463E+00	1.0118	1.494E-02	6.521E-02
**k__Bacteria;p__Cyanobacteria;c__Chloroplast;o__Stramenopiles;f__;g__;s__**	8	7	54	1063	2.393E+00	0.9823	1.485E-02	6.521E-02
**k__Bacteria;p__Proteobacteria;c__Alphaproteobacteria;o__Caulobacterales;f__Caulobacteraceae;g__;s__**	112	26	1968	1393	8.153E-01	0.3371	1.559E-02	6.696E-02
**k__Bacteria;p__Firmicutes;c__Clostridia;o__Clostridiales;f__Lachnospiraceae;g__Oribacterium;s__**	21	9	134	1226	1.787E+00	0.7405	1.582E-02	6.707E-02
**k__Bacteria;p__Firmicutes;c__Clostridia;o__Clostridiales;f__Ruminococcaceae;g__Ruminococcus;s__**	28	8	453	27	-1.629E+00	0.6947	1.904E-02	7.974E-02
**k__Bacteria;p__Proteobacteria;c__Alphaproteobacteria;o__Rhizobiales;f__Rhizobiaceae;g__Agrobacterium;s__**	42	12	325	856	1.243E+00	0.5335	1.979E-02	7.988E-02
**k__Bacteria;p__Bacteroidetes;c__Bacteroidia;o__Bacteroidales;f__Prevotellaceae;g__Prevotella;s__melaninogenica**	80	16	2634	9623	1.133E+00	0.4858	1.975E-02	7.988E-02
**k__Bacteria;p__Proteobacteria;c__Gammaproteobacteria;o__Pseudomonadales;f__Pseudomonadaceae;g__;s__**	144	25	334	41	-4.812E-01	0.2062	1.963E-02	7.988E-02
**k__Bacteria;p__Proteobacteria;c__Gammaproteobacteria;o__Xanthomonadales;f__Xanthomonadaceae;g__Pseudoxanthomonas;s__mexicana**	3	3	4	164	3.242E+00	1.4001	2.058	8.151

© 2021 Hsiao CJ et al. *JAMA Network Open.*

							E-02	E-02
**k__Bacteria;p__Fusobacteria;c__Fusobacteriia;o__Fusobacteriales;f__Leptotrichiaceae;g__Leptotrichia;s__**	73	20	2445	4266	1.107E+00	0.4786	2.068E-02	8.151E-02
**k__Bacteria;p__Cyanobacteria;c__Chloroplast;o__Chlorophyta;f__;g__;s__**	3	4	7	1523	3.435E+00	1.4974	2.180E-02	8.490E-02
**k__Bacteria;p__Proteobacteria;c__Alphaproteobacteria;o__Rhizobiales;f__Phyllobacteriaceae;g__;s__**	16	9	68	137	1.473E+00	0.6459	2.256E-02	8.590E-02
**k__Bacteria;p__Firmicutes;c__Bacilli;o__Gemellales;f__Gemellaceae;g__;s__**	64	14	1204	2580	1.128E+00	0.4946	2.256E-02	8.590E-02
**k__Bacteria;p__Bacteroidetes;c__Sphingobacteriia;o__Sphingobacteriales;f__Sphingobacteriaceae;g__Pedobacter;s__**	60	16	668	9667	1.061E+00	0.4674	2.327E-02	8.757E-02
**k__Bacteria;p__Bacteroidetes;c__Sphingobacteriia;o__Sphingobacteriales;f__Sphingobacteriaceae;g__;s__**	5	7	77	913	2.334E+00	1.0395	2.476E-02	9.215E-02
**k__Bacteria;p__Proteobacteria;c__Betaproteobacteria;o__Burkholderiales;f__Oxalobacteraceae;g__Janthinobacterium;s__**	23	8	70	754	1.551E+00	0.6946	2.557E-02	9.415E-02
**k__Bacteria;p__[Thermi];c__Deinococci;o__Deinococcales;f__Trueperaceae;g__Truepera;s__**	7	5	46	466	2.367E+00	1.0641	2.615E-02	9.521E-02
**k__Bacteria;p__Firmicutes;c__Clostridia;o__Clostridiales;f__Ruminococcaceae;g__;s__**	38	14	428	328	1.165E+00	0.5261	2.682E-02	9.659E-02
**k__Bacteria;p__Actinobacteria;c__Actinobacteria;o__Actinomycetales;f__Nocardioidaceae;g__Aeromicrobium;s__**	2	5	4	136	3.118E+00	1.4196	2.805E-02	9.811E-02
**k__Bacteria;p__Firmicutes;c__Bacilli;o__Turicibacterales;f__Turicibacteraceae;g__Turicibacter;s__**	7	9	65	497	1.827E+00	0.8321	2.811E-02	9.811E-02
**k__Bacteria;p__Actinobacteria;c__Coriobacteriia;o__Coriobacteriales;f__Coriobacteriaceae;g__;s__**	32	12	233	458	1.249E+00	0.5682	2.791E-02	9.811E-02
**k__Bacteria;p__TM6;c__F38;o__;f__;g__;s__**	3	6	3	540	2.789E+00	1.2838	2.982E-02	1.030E-01
**k__Bacteria;p__Proteobacteria;c__Alphaproteobacteria;o__Rhodobacterales;f__Rhodobacteraceae;g__Paracoccus;s__aminovorans**	144	28	272	49	-4.016E-01	0.1856	3.044E-02	1.041E-01
**k__Bacteria;p__Proteobacteria;c__Alphaproteobacteria;o__Rhodospirillales;f__Acetobacteraceae;g__Roseomonas;s__mucosa**	14	2	201	8	-2.809E+00	1.3054	3.139E-02	1.062E-01
**k__Bacteria;p__Actinobacteria;c__Actinobacteria;o__Actinomycetales;f__Geodermatophilaceae;g__;s__**	28	8	159	187	1.385E+00	0.6458	3.197E-02	1.071E-01
**k__Bacteria;p__Proteobacteria;c__Alphaproteobacteria;o__Rhodospirillales;f__;g__;s__**	2	5	3	184	3.030E+00	1.4191	3.277	1.076

© 2021 Hsiao CJ et al. *JAMA Network Open.*

							E-02	E-01
**k__Bacteria;p__Actinobacteria;c__Actinobacteria;o__Actinomycetales;f__Nocardiaceae;g__Rhodococcus;s__**	31	6	131	22359	1.804E+00	0.8445	3.263E-02	1.076E-01
**k__Bacteria;p__Proteobacteria;c__Gammaproteobacteria;o__Pseudomonadales;f__Moraxellaceae;g__Enhydrobacter;s__**	155	29	3146	2308	6.912E-01	0.3270	3.454E-02	1.123E-01
**k__Bacteria;p__Proteobacteria;c__Deltaproteobacteria;o__Myxococcales;f__0319-6G20;g__;s__**	9	3	132	3	-2.445E+00	1.1612	3.525E-02	1.136E-01
**k__Bacteria;p__Actinobacteria;c__Actinobacteria;o__Actinomycetales;f__Micrococcaceae;g__Rothia;s__dentocariosa**	70	16	1208	2122	1.029E+00	0.4897	3.567E-02	1.138E-01
**k__Bacteria;p__Proteobacteria;c__Alphaproteobacteria;o__Rhodobacterales;f__Hyphomonadaceae;g__;s__**	3	6	11	203	2.524E+00	1.2129	3.743E-02	1.183E-01
**k__Bacteria;p__Firmicutes;c__Clostridia;o__Clostridiales;f__Ruminococcaceae;g__Faecalibacterium;s__prausnitzii**	17	9	124	230	1.464E+00	0.7069	3.839E-02	1.202E-01
**k__Bacteria;p__Actinobacteria;c__Actinobacteria;o__Actinomycetales;f__Microbacteriaceae;g__Salinibacterium;s__**	29	9	131	3773	1.432E+00	0.6965	3.975E-02	1.233E-01
**k__Bacteria;p__Proteobacteria;c__Alphaproteobacteria;o__Rhizobiales;f__Methylobacteriaceae;g__;s__**	27	9	207	219	1.162E+00	0.5716	4.198E-02	1.290E-01
**k__Bacteria;p__Actinobacteria;c__Actinobacteria;o__Actinomycetales;f__Micrococcaceae;g__Rothia;s__mucilaginosa**	117	23	7641	20362	9.409E-01	0.4654	4.321E-02	1.316E-01
**k__Bacteria;p__Proteobacteria;c__Gammaproteobacteria;o__Enterobacteriales;f__Enterobacteriaceae;g__Salmonella;s__**	11	5	36	36	1.722E+00	0.8702	4.784E-02	1.444E-01
**k__Bacteria;p__[Thermi];c__Deinococci;o__Deinococcales;f__Deinococcaceae;g__Deinococcus;s__aquatilis**	2	4	2	710	3.205E+00	1.6233	4.833E-02	1.446E-01
**k__Bacteria;p__Proteobacteria;c__Alphaproteobacteria;o__Sphingomonadales;f__Sphingomonadaceae;g__Kaistobacter;s__**	29	12	252	751	1.129E+00	0.5752	4.967E-02	1.472E-01
**k__Bacteria;p__Firmicutes;c__Bacilli;o__Lactobacillales;f__Streptococcaceae;g__Streptococcus;s__anginosus**	30	11	276	854	1.273E+00	0.6514	5.058E-02	1.486E-01
**k__Bacteria;p__Firmicutes;c__Erysipelotrichi;o__Erysipelotrichales;f__Erysipelotrichaceae;g__;s__**	4	5	5	55	2.172E+00	1.1206	5.259E-02	1.532E-01
**k__Bacteria;p__Firmicutes;c__Bacilli;o__Gemellales;f__Gemellaceae;g__Gemella;s__**	145	27	384	99	-3.866E-01	0.2000	5.330E-02	1.539E-01
**k__Bacteria;p__Bacteroidetes;c__Flavobacteriia;o__Flavobacteriales;f__Flavobacteriaceae;g__Capnocytophaga;s__**	48	9	737	461	1.252E+00	0.6507	5.443E-02	1.549E-01
**k__Bacteria;p__Actinobacteria;c__Actinobacteria;o__Actinomycetales;f__Micrococcaceae;g__;s__**	89	15	729	3050	8.533E-01	0.4439	5.457	1.549

© 2021 Hsiao CJ et al. *JAMA Network Open.*

							E-02	E-01
**k__Bacteria;p__Proteobacteria;c__Gammaproteobacteria;o__Pasteurellales;f__Pasteurellaceae;g__Haemophilus;s__**	52	10	1560	143861	1.354E+00	0.7125	5.742E-02	1.616E-01
**k__Bacteria;p__Proteobacteria;c__Alphaproteobacteria;o__Rhodobacterales;f__Rhodobacteraceae;g__Rubellimicrobium;s__**	20	8	176	1244	1.486E+00	0.7847	5.829E-02	1.627E-01
**k__Bacteria;p__Firmicutes;c__Clostridia;o__Clostridiales;f__Lachnospiraceae;g__Coprococcus;s__**	6	3	66	3	-2.246E+00	1.1930	5.971E-02	1.653E-01
**k__Bacteria;p__Proteobacteria;c__Epsilonproteobacteria;o__Campylobacterales;f__Campylobacteraceae;g__Arcobacter;s__**	8	6	17	744	1.809E+00	0.9689	6.191E-02	1.700E-01
**k__Bacteria;p__Actinobacteria;c__Thermoleophilia;o__Solirubrobacterales;f__Conexibacteraceae;g__;s__**	2	6	3	180	2.620E+00	1.4087	6.285E-02	1.701E-01
**k__Bacteria;p__Proteobacteria;c__Deltaproteobacteria;o__Bdellovibrionales;f__Bdellovibrionaceae;g__Bdellovibrio;s__**	14	6	66	188	1.401E+00	0.7533	6.296E-02	1.701E-01
**k__Bacteria;p__Firmicutes;c__Bacilli;o__Lactobacillales;f__Carnobacteriaceae;g__Carnobacterium;s__**	38	5	287	13	-1.568E+00	0.8501	6.517E-02	1.733E-01
**k__Bacteria;p__Firmicutes;c__Bacilli;o__Bacillales;f__Bacillaceae;g__Bacillus;s__**	43	15	1827	588	9.205E-01	0.4986	6.487E-02	1.733E-01
**k__Bacteria;p__Actinobacteria;c__Actinobacteria;o__Actinomycetales;f__Nocardioidaceae;g__Nocardioides;s__plantarum**	2	5	2	94	2.598E+00	1.4177	6.692E-02	1.765E-01
**k__Bacteria;p__Proteobacteria;c__Betaproteobacteria;o__Rhodocyclales;f__Rhodocyclaceae;g__Zoogloea;s__**	2	6	2	245	2.749E+00	1.5039	6.751E-02	1.767E-01
**k__Bacteria;p__Proteobacteria;c__Alphaproteobacteria;o__Sphingomonadales;f__Sphingomonadaceae;g__Sphingomonas;s__echinoides**	38	10	328	1379	1.021E+00	0.5608	6.857E-02	1.781E-01
**k__Bacteria;p__Firmicutes;c__Bacilli;o__Lactobacillales;f__Enterococcaceae;g__;s__**	28	8	530	32	-1.208E+00	0.6777	7.466E-02	1.924E-01
**k__Bacteria;p__Firmicutes;c__Clostridia;o__Clostridiales;f__Peptococcaceae;g__Peptococcus;s__**	7	4	33	30	1.732E+00	0.9784	7.666E-02	1.960E-01
**k__Bacteria;p__Firmicutes;c__Clostridia;o__Clostridiales;f__[Tissierellaceae];g__Finegoldia;s__**	141	23	50194	3616	-8.165E-01	0.4633	7.798E-02	1.979E-01
**k__Bacteria;p__Proteobacteria;c__Alphaproteobacteria;o__Rhizobiales;f__Phyllobacteriaceae;g__Mesorhizobium;s__**	4	6	26	344	1.870E+00	1.0638	7.883E-02	1.985E-01
**k__Bacteria;p__TM7;c__TM7-3;o__EW055;f__;g__;s__**	2	8	9	284	2.431E+00	1.3912	8.051E-02	2.013E-01
**k__Bacteria;p__Proteobacteria;c__Betaproteobacteria;o__Burkholderiales;f__Comamonadaceae;g__Limnohabitans;s__**	3	8	9	212	1.945E+00	1.1271	8.433	2.093

							E-02	E-01
**k__Bacteria;p__Proteobacteria;c__Betaproteobacteria;o__Burkholderiales;f__Oxalobacteraceae;g__Ralstonia;s__**	128	27	1312	709	6.225E-01	0.3644	8.753E-02	2.156E-01
**k__Bacteria;p__Firmicutes;c__Clostridia;o__Clostridiales;f__Veillonellaceae;g__Megamonas;s__**	2	3	29	223	2.665E+00	1.5836	9.239E-02	2.259E-01
**k__Bacteria;p__Proteobacteria;c__Betaproteobacteria;o__Neisseriales;f__Neisseriaceae;g__Neisseria;s__**	84	16	4172	4899	8.963E-01	0.5338	9.313E-02	2.261E-01
**k__Bacteria;p__Firmicutes;c__Clostridia;o__Clostridiales;f__Lachnospiraceae;g__Catonella;s__**	2	3	32	186	2.673E+00	1.6009	9.499E-02	2.289E-01
**k__Bacteria;p__Fusobacteria;c__Fusobacteriia;o__Fusobacteriales;f__Leptotrichiaceae;g__;s__**	2	4	2	81	2.519E+00	1.5619	1.068E-01	2.556E-01
**k__Bacteria;p__Bacteroidetes;c__[Saprospirae];o__[Saprospirales];f__Chitinophagaceae;g__;s__**	2	7	25	245	2.030E+00	1.2655	1.087E-01	2.558E-01
**k__Bacteria;p__Firmicutes;c__Bacilli;o__Bacillales;f__Staphylococcaceae;g__Jeotgalicoccus;s__**	21	4	543	206	1.522E+00	0.9503	1.092E-01	2.558E-01
**k__Bacteria;p__Firmicutes;c__Bacilli;o__Lactobacillales;f__Streptococcaceae;g__Streptococcus;s__minor**	7	7	19	87	1.388E+00	0.8658	1.089E-01	2.558E-01
**k__Bacteria;p__Actinobacteria;c__Actinobacteria;o__Actinomycetales;f__Nocardioidaceae;g__Friedmanniella;s__**	8	3	46	5	-1.721E+00	1.0793	1.108E-01	2.578E-01
**k__Bacteria;p__Actinobacteria;c__Actinobacteria;o__Bifidobacteriales;f__Bifidobacteriaceae;g__Scardovia;s__**	10	6	147	124	1.584E+00	0.9975	1.124E-01	2.597E-01
**k__Bacteria;p__Actinobacteria;c__Actinobacteria;o__Actinomycetales;f__Sporichthyaceae;g__;s__**	6	5	21	70	1.527E+00	0.9722	1.163E-01	2.668E-01
**k__Bacteria;p__Proteobacteria;c__Betaproteobacteria;o__Burkholderiales;f__Comamonadaceae;g__Hydrogenophaga;s__**	4	5	16	1782	2.010E+00	1.2917	1.197E-01	2.694E-01
**k__Bacteria;p__Bacteroidetes;c__Flavobacteriia;o__Flavobacteriales;f__[Weeksellaceae];g__;s__**	16	5	107	130	1.327E+00	0.8531	1.198E-01	2.694E-01
**k__Bacteria;p__Proteobacteria;c__Betaproteobacteria;o__Burkholderiales;f__Burkholderiaceae;g__Lautropia;s__**	21	6	231	136	1.176E+00	0.7540	1.188E-01	2.694E-01
**k__Bacteria;p__Firmicutes;c__Bacilli;o__Lactobacillales;f__Lactobacillaceae;g__Lactobacillus;s__iners**	55	9	406	35	-7.910E-01	0.5135	1.235E-01	2.757E-01
**k__Bacteria;p__Verrucomicrobia;c__[Spartobacteria];o__[Chthoniobacterales];f__[Chthoniobacteraceae];g__CandidatusXiphinematobacter;s__**	2	3	44	195	2.444E+00	1.5927	1.249E-01	2.771E-01
**k__Bacteria;p__Actinobacteria;c__Actinobacteria;o__Bifidobacteriales;f__Bifidobacteriaceae;g__Bifidobacterium;s__adolescentis**	17	8	74	149	9.095E-01	0.6082	1.348	2.951

© 2021 Hsiao CJ et al. *JAMA Network Open.*

							E-01	E-01
**k__Bacteria;p__Bacteroidetes;c__Bacteroidia;o__Bacteroidales;f__[Paraprevotellaceae];g__[Prevotella];s__**	42	10	576	433	9.018E-01	0.6018	1.340E-01	2.951E-01
**k__Bacteria;p__Bacteroidetes;c__Flavobacteriia;o__Flavobacteriales;f__[Weeksellaceae];g__Elizabethkingia;s__meningoseptica**	12	4	16	104	1.365E+00	0.9270	1.409E-01	3.065E-01
**k__Bacteria;p__Bacteroidetes;c__Bacteroidia;o__Bacteroidales;f__Porphyromonadaceae;g__Porphyromonas;s__**	74	16	1959	769	7.321E-01	0.4998	1.430E-01	3.090E-01
**k__Bacteria;p__Firmicutes;c__Clostridia;o__Clostridiales;f__Veillonellaceae;g__Dialister;s__**	62	13	2830	272	-8.118E-01	0.5635	1.497E-01	3.215E-01
**k__Bacteria;p__Proteobacteria;c__Gammaproteobacteria;o__Pseudomonadales;f__Pseudomonadaceae;g__Pseudomonas;s__fragi**	114	23	2237	835	4.980E-01	0.3523	1.575E-01	3.360E-01
**k__Bacteria;p__Firmicutes;c__Bacilli;o__Lactobacillales;f__Lactobacillaceae;g__Lactobacillus;s__zeae**	3	3	33	32	2.042E+00	1.4619	1.624E-01	3.444E-01
**k__Bacteria;p__Proteobacteria;c__Betaproteobacteria;o__Burkholderiales;f__Comamonadaceae;g__Tepidimonas;s__**	47	2	281	4	-1.712E+00	1.2421	1.680E-01	3.540E-01
**k__Bacteria;p__Proteobacteria;c__Alphaproteobacteria;o__Rhodobacterales;f__Rhodobacteraceae;g__Paracoccus;s__**	49	12	275	209	6.901E-01	0.5062	1.728E-01	3.617E-01
**k__Bacteria;p__Proteobacteria;c__Betaproteobacteria;o__SBla14;f__;g__;s__**	2	3	5	5	2.086E+00	1.5513	1.787E-01	3.706E-01
**k__Bacteria;p__Proteobacteria;c__Alphaproteobacteria;o__Rhizobiales;f__Aurantimonadaceae;g__;s__**	17	6	41	72	1.002E+00	0.7482	1.803E-01	3.706E-01
**k__Bacteria;p__Actinobacteria;c__Actinobacteria;o__Actinomycetales;f__Micrococcaceae;g__Arthrobacter;s__**	19	10	142	179	8.532E-01	0.6365	1.801E-01	3.706E-01
**k__Bacteria;p__Bacteroidetes;c__[Saprospirae];o__[Saprospirales];f__Chitinophagaceae;g__Flavisolibacter;s__**	6	3	585	345	1.904E+00	1.4473	1.884E-01	3.758E-01
**k__Bacteria;p__Synergistetes;c__Synergistia;o__Synergistales;f__Synergistaceae;g__vadinCA02;s__**	4	3	4	49	1.614E+00	1.2300	1.895E-01	3.758E-01
**k__Bacteria;p__Actinobacteria;c__Actinobacteria;o__Actinomycetales;f__Dermacoccaceae;g__Dermacoccus;s__**	22	3	880	10	-1.522E+00	1.1527	1.868E-01	3.758E-01
**k__Bacteria;p__Proteobacteria;c__Betaproteobacteria;o__Burkholderiales;f__Comamonadaceae;g__Acidovorax;s__delafieldii**	4	6	36	533	1.519E+00	1.1535	1.878E-01	3.758E-01
**k__Bacteria;p__Proteobacteria;c__Gammaproteobacteria;o__Vibrionales;f__Pseudoalteromonadaceae;g__Pseudoalteromonas;s__**	12	4	125	217	1.389E+00	1.0619	1.907E-01	3.758E-01
**k__Bacteria;p__Actinobacteria;c__Actinobacteria;o__Actinomycetales;f__Micrococcaceae;g__Micrococcus;s__luteus**	21	5	66	11	-1.01	0.7670	1.869	3.758

© 2021 Hsiao CJ et al. *JAMA Network Open.*

					2E+00		E-01	E-01
**k__Bacteria;p__Proteobacteria;c__Betaproteobacteria;o__Burkholderiales;f__Comamonadaceae;g__Delftia;s__**	135	28	6776	3663	4.377E-01	0.3339	1.900E-01	3.758E-01
**k__Bacteria;p__Bacteroidetes;c__Bacteroidia;o__Bacteroidales;f__Prevotellaceae;g__Prevotella;s__intermedia**	5	2	49	83	1.869E+00	1.4520	1.980E-01	3.875E-01
**k__Bacteria;p__Firmicutes;c__Clostridia;o__Clostridiales;f__Veillonellaceae;g__Selenomonas;s__**	37	12	802	320	7.577E-01	0.5898	1.989E-01	3.875E-01
**k__Bacteria;p__Proteobacteria;c__Alphaproteobacteria;o__Rhizobiales;f__Bradyrhizobiaceae;g__Bradyrhizobium;s__**	146	27	405	64	-2.533E-01	0.1979	2.006E-01	3.884E-01
**k__Bacteria;p__Bacteroidetes;c__Bacteroidia;o__Bacteroidales;f__Prevotellaceae;g__Prevotella;s__nanceiensis**	20	5	120	32	1.000E+00	0.7897	2.054E-01	3.954E-01
**k__Bacteria;p__Proteobacteria;c__Deltaproteobacteria;o__Syntrophobacterales;f__Syntrophobacteraceae;g__;s__**	2	4	2	96	1.930E+00	1.5439	2.112E-01	3.957E-01
**k__Bacteria;p__Actinobacteria;c__Actinobacteria;o__Actinomycetales;f__Microbacteriaceae;g__Leucobacter;s__**	7	8	54	164	1.201E+00	0.9569	2.096E-01	3.957E-01
**k__Bacteria;p__Proteobacteria;c__Gammaproteobacteria;o__Pasteurellales;f__Pasteurellaceae;g__Aggregatibacter;s__segnis**	12	5	123	122	1.137E+00	0.9097	2.114E-01	3.957E-01
**k__Bacteria;p__Firmicutes;c__Bacilli;o__Bacillales;f__Planococcaceae;g__Planomicrobium;s__**	23	9	114	453	8.874E-01	0.7076	2.099E-01	3.957E-01
**k__Bacteria;p__Firmicutes;c__Bacilli;o__Lactobacillales;f__Aerococcaceae;g__Facklamia;s__**	36	6	325	57	-8.844E-01	0.7026	2.082E-01	3.957E-01
**k__Bacteria;p__Actinobacteria;c__Thermoleophilia;o__Solirubrobacterales;f__Solirubrobacteraceae;g__;s__**	6	5	12	40	1.239E+00	0.9971	2.138E-01	3.980E-01
**k__Bacteria;p__Firmicutes;c__Clostridia;o__Clostridiales;f__[Tissierellaceae];g__Gallicola;s__**	8	3	83	342	1.636E+00	1.3325	2.195E-01	4.042E-01
**k__Bacteria;p__Firmicutes;c__Clostridia;o__Clostridiales;f__Clostridiaceae;g__Clostridium;s__**	36	11	242	175	6.609E-01	0.5384	2.196E-01	4.042E-01
**k__Bacteria;p__Actinobacteria;c__Actinobacteria;o__Actinomycetales;f__Corynebacteriaceae;g__Corynebacterium;s__durum**	37	6	458	622	9.402E-01	0.7754	2.253E-01	4.124E-01
**k__Bacteria;p__Actinobacteria;c__Actinobacteria;o__Actinomycetales;f__Actinomycetaceae;g__;s__**	7	4	22	15	1.202E+00	1.0087	2.333E-01	4.247E-01
**k__Bacteria;p__Firmicutes;c__Clostridia;o__Clostridiales;f__Veillonellaceae;g__Veillonella;s__parvula**	15	5	80	75	9.425E-01	0.8043	2.413E-01	4.369E-01
**k__Bacteria;p__Firmicutes;c__Clostridia;o__Clostridiales;f__[Tissierellaceae];g__Peptoniphilus;s__**	137	24	61085	10405	-5.60	0.4828	2.457	4.425

© 2021 Hsiao CJ et al. *JAMA Network Open.*

					5E-01		E-01	E-01
**k__Bacteria;p__Proteobacteria;c__Betaproteobacteria;o__Rhodocyclales;f__Rhodocyclaceae;g__Dechloromonas;s__**	2	8	17	343	1.720E+00	1.4954	2.499E-01	4.453E-01
**k__Bacteria;p__Proteobacteria;c__Gammaproteobacteria;o__Pasteurellales;f__Pasteurellaceae;g__Haemophilus;s__parainfluenzae**	66	10	1794	3352	7.477E-01	0.6511	2.508E-01	4.453E-01
**k__Bacteria;p__Actinobacteria;c__Actinobacteria;o__Actinomycetales;f__Propionibacteriaceae;g__;s__**	19	9	86	97	7.272E-01	0.6338	2.512E-01	4.453E-01
**k__Bacteria;p__Firmicutes;c__Clostridia;o__Clostridiales;f__Lachnospiraceae;g__[Ruminococcus];s__**	14	9	51	48	7.382E-01	0.6482	2.548E-01	4.492E-01
**k__Bacteria;p__Firmicutes;c__Bacilli;o__Bacillales;f__Bacillaceae;g__Geobacillus;s__**	50	9	1066	118	-7.666E-01	0.6845	2.627E-01	4.608E-01
**k__Bacteria;p__Actinobacteria;c__Actinobacteria;o__Actinomycetales;f__Microbacteriaceae;g__Rathayibacter;s__caricis**	6	2	367	116	1.703E+00	1.5927	2.849E-01	4.917E-01
**k__Bacteria;p__Firmicutes;c__Bacilli;o__Lactobacillales;f__Enterococcaceae;g__Enterococcus;s__**	11	4	72	14	-1.103E+00	1.0327	2.855E-01	4.917E-01
**k__Bacteria;p__Proteobacteria;c__Gammaproteobacteria;o__Enterobacteriales;f__Enterobacteriaceae;g__Morganella;s__**	71	3	698	31	-1.062E+00	0.9944	2.857E-01	4.917E-01
**k__Bacteria;p__Firmicutes;c__Bacilli;o__Lactobacillales;f__Aerococcaceae;g__Abiotrophia;s__**	33	6	570	506	8.137E-01	0.7639	2.868E-01	4.917E-01
**k__Bacteria;p__Proteobacteria;c__Gammaproteobacteria;o__Pasteurellales;f__Pasteurellaceae;g__Aggregatibacter;s__**	17	9	320	96	8.028E-01	0.7550	2.877E-01	4.917E-01
**k__Bacteria;p__Bacteroidetes;c__Flavobacteriia;o__Flavobacteriales;f__Flavobacteriaceae;g__Capnocytophaga;s__ochracea**	3	3	5	7	1.412E+00	1.3371	2.909E-01	4.922E-01
**k__Bacteria;p__Proteobacteria;c__Betaproteobacteria;o__Burkholderiales;f__Burkholderiaceae;g__Burkholderia;s__**	145	30	768	2251	2.530E-01	0.2389	2.896E-01	4.922E-01
**k__Bacteria;p__Proteobacteria;c__Betaproteobacteria;o__Burkholderiales;f__Comamonadaceae;g__Acidovorax;s__**	2	3	7	102	1.796E+00	1.7097	2.934E-01	4.940E-01
**k__Bacteria;p__Spirochaetes;c__Spirochaetes;o__Spirochaetales;f__Spirochaetaceae;g__Treponema;s__**	3	2	31	29	1.666E+00	1.5967	2.966E-01	4.964E-01
**k__Bacteria;p__Fusobacteria;c__Fusobacteriia;o__Fusobacteriales;f__Leptotrichiaceae;g__Sneathia;s__**	13	3	230	4	-1.141E+00	1.0959	2.978E-01	4.964E-01
**k__Bacteria;p__Firmicutes;c__Bacilli;o__Bacillales;f__Staphylococcaceae;g__Staphylococcus;s__**	167	30	131054	4931	-1.554E+00	1.5058	3.022E-01	4.986E-01
**k__Bacteria;p__Firmicutes;c__Clostridia;o__Clostridiales;f__Lachnospiraceae;g__Lachnospira;s__**	4	2	9	34	1.526E+00	1.4757	3.011	4.986

© 2021 Hsiao CJ et al. *JAMA Network Open.*

							E-01	E-01
**k__Bacteria;p__Proteobacteria;c__Gammaproteobacteria;o__Enterobacteriales;f__Enterobacteriaceae;g__Serratia;s__marcescens**	11	2	1161	3	-1.575E+00	1.5516	3.101E-01	5.093E-01
**k__Bacteria;p__Firmicutes;c__Clostridia;o__Clostridiales;f__[Mogibacteriaceae];g__;s__**	14	4	58	62	9.428E-01	0.9419	3.168E-01	5.177E-01
**k__Bacteria;p__Proteobacteria;c__Betaproteobacteria;o__Burkholderiales;f__Comamonadaceae;g__Polaromonas;s__**	17	7	32	58	6.283E-01	0.6407	3.268E-01	5.314E-01
**k__Bacteria;p__Firmicutes;c__Clostridia;o__Clostridiales;f__[Tissierellaceae];g__1-68;s__**	17	4	334	387	9.977E-01	1.0300	3.327E-01	5.359E-01
**k__Bacteria;p__Proteobacteria;c__Gammaproteobacteria;o__Pseudomonadales;f__Moraxellaceae;g__Acinetobacter;s__**	162	29	8909	2257	3.163E-01	0.3258	3.317E-01	5.359E-01
**k__Bacteria;p__Actinobacteria;c__Actinobacteria;o__Actinomycetales;f__Pseudonocardiaceae;g__Pseudonocardia;s__**	9	2	51	10	-1.277E+00	1.3381	3.400E-01	5.400E-01
**k__Bacteria;p__Actinobacteria;c__Actinobacteria;o__Actinomycetales;f__Pseudonocardiaceae;g__Actinomycetospora;s__**	5	4	14	10	1.041E+00	1.0915	3.404E-01	5.400E-01
**k__Bacteria;p__Proteobacteria;c__Alphaproteobacteria;o__Caulobacterales;f__Caulobacteraceae;g__Brevundimonas;s__diminuta**	19	6	163	189	7.982E-01	0.8332	3.381E-01	5.400E-01
**k__Bacteria;p__Firmicutes;c__Clostridia;o__Clostridiales;f__Veillonellaceae;g__Megasphaera;s__**	30	8	279	126	5.968E-01	0.6277	3.417E-01	5.400E-01
**k__Bacteria;p__Firmicutes;c__Clostridia;o__Thermoanaerobacterales;f__Caldicellulosiruptoraceae;g__Caldicellulosiruptor;s__saccharolyticus**	13	4	138	38	-9.405E-01	0.9929	3.435E-01	5.403E-01
**k__Bacteria;p__Firmicutes;c__Bacilli;o__Bacillales;f__Staphylococcaceae;g__Staphylococcus;s__epidermidis**	167	30	15399	702	-1.269E+00	1.3450	3.455E-01	5.409E-01
**k__Bacteria;p__Actinobacteria;c__Actinobacteria;o__Actinomycetales;f__Yaniellaceae;g__Yaniella;s__**	4	2	14	9	1.324E+00	1.4135	3.489E-01	5.437E-01
**k__Bacteria;p__Proteobacteria;c__Betaproteobacteria;o__Burkholderiales;f__Comamonadaceae;g__Comamonas;s__**	29	5	339	74	-7.505E-01	0.8118	3.552E-01	5.509E-01
**k__Bacteria;p__Firmicutes;c__Clostridia;o__Clostridiales;f__Lachnospiraceae;g__[Ruminococcus];s__gnavus**	3	4	64	190	1.370E+00	1.4989	3.609E-01	5.545E-01
**k__Bacteria;p__Cyanobacteria;c__4C0d-2;o__MLE1-12;f__;g__;s__**	116	19	2903	5222	3.830E-01	0.4186	3.602E-01	5.545E-01
**k__Bacteria;p__Proteobacteria;c__Alphaproteobacteria;o__Rhizobiales;f__Brucellaceae;g__;s__**	22	9	74	34	5.361E-01	0.5971	3.693E-01	5.623E-01
**k__Bacteria;p__Proteobacteria;c__Gammaproteobacteria;o__Pseudomonadales;f__Pseudomonadaceae;g__Pseudomonas;s__viridiflava**	47	14	673	741	4.714E-01	0.5242	3.685	5.623

© 2021 Hsiao CJ et al. *JAMA Network Open.*

							E-01	E-01
**k__Bacteria;p__Firmicutes;c__Bacilli;o__Bacillales;f__Planococcaceae;g__;s__**	167	30	228426	13928	-1.484E+00	1.6665	3.731E-01	5.637E-01
**k__Bacteria;p__Proteobacteria;c__Gammaproteobacteria;o__Enterobacteriales;f__Enterobacteriaceae;g__Proteus;s__**	21	5	4981	852	1.025E+00	1.1523	3.735E-01	5.637E-01
**k__Bacteria;p__Bacteroidetes;c__[Saprospirae];o__[Saprospirales];f__Chitinophagaceae;g__Sediminibacterium;s__**	109	19	3014	438	-3.557E-01	0.4073	3.824E-01	5.744E-01
**k__Bacteria;p__Actinobacteria;c__Actinobacteria;o__Actinomycetales;f__Sanguibacteraceae;g__Sanguibacter;s__**	3	4	5	30	1.176E+00	1.3550	3.852E-01	5.761E-01
**k__Bacteria;p__Proteobacteria;c__Betaproteobacteria;o__Neisseriales;f__Neisseriaceae;g__Eikenella;s__**	6	4	28	20	9.324E-01	1.0910	3.927E-01	5.796E-01
**k__Bacteria;p__Proteobacteria;c__Betaproteobacteria;o__Neisseriales;f__Neisseriaceae;g__Kingella;s__**	14	4	137	66	8.656E-01	1.0109	3.919E-01	5.796E-01
**k__Bacteria;p__Proteobacteria;c__Alphaproteobacteria;o__Rhizobiales;f__Methylobacteriaceae;g__Methylobacterium;s__adhaesivum**	22	6	115	107	6.179E-01	0.7207	3.913E-01	5.796E-01
**k__Bacteria;p__Proteobacteria;c__Betaproteobacteria;o__Neisseriales;f__Neisseriaceae;g__Neisseria;s__subflava**	10	2	87	10	-1.140E+00	1.3583	4.013E-01	5.812E-01
**k__Bacteria;p__Bacteroidetes;c__Bacteroidia;o__Bacteroidales;f__Porphyromonadaceae;g__Paludibacter;s__**	8	5	47	62	8.571E-01	1.0210	4.012E-01	5.812E-01
**k__Bacteria;p__Proteobacteria;c__Alphaproteobacteria;o__Caulobacterales;f__Caulobacteraceae;g__Mycoplana;s__**	21	8	131	184	5.588E-01	0.6675	4.025E-01	5.812E-01
**k__Bacteria;p__Proteobacteria;c__Gammaproteobacteria;o__Xanthomonadales;f__Xanthomonadaceae;g__;s__**	107	23	1698	798	2.949E-01	0.3497	3.991E-01	5.812E-01
**k__Bacteria;p__Proteobacteria;c__Gammaproteobacteria;o__Pseudomonadales;f__Moraxellaceae;g__Acinetobacter;s__johnsonii**	151	26	825	281	-2.245E-01	0.2644	3.959E-01	5.812E-01
**k__Bacteria;p__Proteobacteria;c__Deltaproteobacteria;o__Myxococcales;f__;g__;s__**	29	5	286	200	7.084E-01	0.8509	4.052E-01	5.825E-01
**k__Bacteria;p__Proteobacteria;c__Betaproteobacteria;o__Burkholderiales;f__Comamonadaceae;g__Hylemonella;s__**	3	4	24	7	-1.064E+00	1.2936	4.107E-01	5.865E-01
**k__Bacteria;p__Bacteroidetes;c__[Saprospirae];o__[Saprospirales];f__Saprospiraceae;g__;s__**	4	6	70	143	9.640E-01	1.1737	4.115E-01	5.865E-01
**k__Bacteria;p__Proteobacteria;c__Alphaproteobacteria;o__Rhizobiales;f__Hyphomicrobiaceae;g__;s__**	2	5	10	84	1.152E+00	1.4116	4.146E-01	5.885E-01
**k__Bacteria;p__Bacteroidetes;c__Flavobacteriia;o__Flavobacteriales;f__[Weeksellaceae];g__Chryseobacterium;s__**	153	29	2726	9296	2.616E-01	0.3238	4.191	5.925

© 2021 Hsiao CJ et al. *JAMA Network Open.*

							E-01	E-01
**k__Bacteria;p__Actinobacteria;c__Actinobacteria;o__Actinomycetales;f__Corynebacteriaceae;g__Corynebacterium;s__**	166	29	1822190	120246	-1.448E+00	1.8067	4.227E-01	5.950E-01
**k__Bacteria;p__Firmicutes;c__Bacilli;o__Bacillales;f__Staphylococcaceae;g__Staphylococcus;s__aureus**	166	28	21401	433	-1.326E+00	1.6644	4.258E-01	5.968E-01
**k__Bacteria;p__WPS-2;c__;o__;f__;g__;s__**	5	5	115	308	8.729E-01	1.1174	4.347E-01	6.068E-01
**k__Bacteria;p__Proteobacteria;c__Gammaproteobacteria;o__Cardiobacteriales;f__Cardiobacteriaceae;g__Cardiobacterium;s__**	6	2	18	9	1.044E+00	1.3441	4.375E-01	6.082E-01
**k__Bacteria;p__Proteobacteria;c__Gammaproteobacteria;o__Enterobacteriales;f__Enterobacteriaceae;g__Klebsiella;s__**	9	2	310	36	-1.196E+00	1.5713	4.467E-01	6.184E-01
**k__Bacteria;p__Bacteroidetes;c__Bacteroidia;o__Bacteroidales;f__Prevotellaceae;g__Prevotella;s__copri**	9	5	140	103	-7.606E-01	1.0096	4.512E-01	6.221E-01
**k__Bacteria;p__Actinobacteria;c__Actinobacteria;o__Actinomycetales;f__Dermabacteraceae;g__Dermabacter;s__**	39	7	301	56	-4.893E-01	0.6576	4.568E-01	6.272E-01
**k__Bacteria;p__Bacteroidetes;c__Cytophagia;o__Cytophagales;f__Cytophagaceae;g__Spirosoma;s__**	3	2	64	747	1.213E+00	1.6503	4.625E-01	6.323E-01
**k__Bacteria;p__Proteobacteria;c__Gammaproteobacteria;o__Oceanospirillales;f__Halomonadaceae;g__Halomonas;s__**	162	29	3507	1138	1.912E-01	0.2624	4.661E-01	6.348E-01
**k__Bacteria;p__SR1;c__;o__;f__;g__;s__**	4	4	24	57	8.874E-01	1.2262	4.693E-01	6.364E-01
**k__Bacteria;p__Actinobacteria;c__Actinobacteria;o__Actinomycetales;f__Nakamurellaceae;g__;s__**	6	7	21	51	7.288E-01	1.0132	4.719E-01	6.375E-01
**k__Bacteria;p__Bacteroidetes;c__Bacteroidia;o__Bacteroidales;f__Porphyromonadaceae;g__Parabacteroides;s__**	5	2	10	13	1.018E+00	1.4339	4.776E-01	6.396E-01
**k__Bacteria;p__Bacteroidetes;c__Bacteroidia;o__Bacteroidales;f__Bacteroidaceae;g__Bacteroides;s__uniformis**	17	8	166	102	5.183E-01	0.7324	4.792E-01	6.396E-01
**k__Bacteria;p__Proteobacteria;c__Gammaproteobacteria;o__Alteromonadales;f__Shewanellaceae;g__Shewanella;s__algae**	157	29	2115	714	1.848E-01	0.2590	4.756E-01	6.396E-01
**k__Bacteria;p__Actinobacteria;c__Actinobacteria;o__Actinomycetales;f__Micromonosporaceae;g__;s__**	5	4	65	10	-8.298E-01	1.1918	4.863E-01	6.464E-01
**k__Bacteria;p__Actinobacteria;c__Actinobacteria;o__Actinomycetales;f__Propionibacteriaceae;g__Propionibacterium;s__acnes**	166	30	320674	21902	-1.216E+00	1.8046	5.006E-01	6.571E-01
**k__Bacteria;p__Tenericutes;c__Mollicutes;o__Mycoplasmatales;f__Mycoplasmataceae;g__Mycoplasma;s__**	3	4	71	42	-9.04	1.3429	5.008	6.571

© 2021 Hsiao CJ et al. *JAMA Network Open.*

					1E-01		E-01	E-01
**k__Bacteria;p__Actinobacteria;c__Actinobacteria;o__Actinomycetales;f__Corynebacteriaceae;g__Corynebacterium;s__lubricantis**	8	4	48	122	6.601E-01	0.9835	5.021E-01	6.571E-01
**k__Bacteria;p__Proteobacteria;c__Alphaproteobacteria;o__Sphingomonadales;f__Sphingomonadaceae;g__Sphingobium;s__**	37	9	249	189	3.983E-01	0.5875	4.978E-01	6.571E-01
**k__Bacteria;p__Firmicutes;c__Clostridia;o__Clostridiales;f__Veillonellaceae;g__Phascolarctobacterium;s__**	6	4	52	50	-7.265E-01	1.1022	5.098E-01	6.645E-01
**k__Bacteria;p__Actinobacteria;c__Coriobacteriia;o__Coriobacteriales;f__Coriobacteriaceae;g__Eggerthella;s__lenta**	2	4	10	20	9.825E-01	1.5119	5.158E-01	6.685E-01
**k__Bacteria;p__Bacteroidetes;c__Bacteroidia;o__Bacteroidales;f__[Paraprevotellaceae];g__[Prevotella];s__tannerae**	13	2	98	51	-8.819E-01	1.3660	5.185E-01	6.685E-01
**k__Bacteria;p__Proteobacteria;c__Gammaproteobacteria;o__Alteromonadales;f__Shewanellaceae;g__Shewanella;s__**	17	8	101	13	4.095E-01	0.6357	5.195E-01	6.685E-01
**k__Bacteria;p__Proteobacteria;c__Alphaproteobacteria;o__Rhizobiales;f__Methylobacteriaceae;g__Methylobacterium;s__**	47	11	496	130	3.582E-01	0.5578	5.208E-01	6.685E-01
**k__Bacteria;p__Actinobacteria;c__Actinobacteria;o__Actinomycetales;f__Propionibacteriaceae;g__Microlunatus;s__**	3	3	9	24	8.724E-01	1.3797	5.272E-01	6.691E-01
**k__Bacteria;p__Actinobacteria;c__Actinobacteria;o__Actinomycetales;f__Geodermatophilaceae;g__Modestobacter;s__**	10	3	113	7	-7.207E-01	1.1453	5.292E-01	6.691E-01
**k__Bacteria;p__Firmicutes;c__Bacilli;o__Lactobacillales;f__Lactobacillaceae;g__Lactobacillus;s__reuteri**	13	4	126	525	7.115E-01	1.1347	5.306E-01	6.691E-01
**k__Bacteria;p__Actinobacteria;c__Actinobacteria;o__Actinomycetales;f__Dermabacteraceae;g__Brachybacterium;s__conglomeratum**	35	7	191	50	4.018E-01	0.6418	5.313E-01	6.691E-01
**k__Bacteria;p__Proteobacteria;c__Betaproteobacteria;o__Neisseriales;f__Neisseriaceae;g__;s__**	124	22	44053	4758	-3.474E-01	0.5483	5.263E-01	6.691E-01
**k__Bacteria;p__Acidobacteria;c__Acidobacteria-6;o__iii1-15;f__;g__;s__**	6	4	145	73	-7.406E-01	1.1914	5.342E-01	6.693E-01
**k__Bacteria;p__Proteobacteria;c__Gammaproteobacteria;o__Xanthomonadales;f__Sinobacteraceae;g__;s__**	80	20	1013	280	2.239E-01	0.3613	5.354E-01	6.693E-01
**k__Bacteria;p__Proteobacteria;c__Alphaproteobacteria;o__;f__;g__;s__**	63	12	609	657	3.024E-01	0.4989	5.444E-01	6.779E-01
**k__Bacteria;p__Firmicutes;c__Clostridia;o__Clostridiales;f__;g__;s__**	20	9	142	121	3.865E-01	0.6421	5.472E-01	6.790E-01
**k__Bacteria;p__Firmicutes;c__Clostridia;o__Clostridiales;f__[Mogibacteriaceae];g__Mogibacterium;s__**	5	3	11	6	7.148E-01	1.2035	5.525	6.830

© 2021 Hsiao CJ et al. *JAMA Network Open.*

							E-01	E-01
**k__Bacteria;p__Firmicutes;c__Clostridia;o__Clostridiales;f__[Tissierellaceae];g__WAL_1855D;s__**	21	3	707	1400	7.314E-01	1.2565	5.605E-01	6.903E-01
**k__Bacteria;p__Proteobacteria;c__Alphaproteobacteria;o__Rhizobiales;f__Phyllobacteriaceae;g__Aminobacter;s__**	8	6	41	116	5.060E-01	0.9040	5.756E-01	7.064E-01
**k__Bacteria;p__Proteobacteria;c__Alphaproteobacteria;o__Rhizobiales;f__Phyllobacteriaceae;g__Phyllobacterium;s__**	144	30	490	535	1.304E-01	0.2346	5.784E-01	7.072E-01
**k__Bacteria;p__Actinobacteria;c__Actinobacteria;o__Actinomycetales;f__Nocardiaceae;g__Rhodococcus;s__fascians**	4	2	13	4	-7.966E-01	1.4688	5.876E-01	7.157E-01
**k__Bacteria;p__Proteobacteria;c__Gammaproteobacteria;o__Pasteurellales;f__Pasteurellaceae;g__Actinobacillus;s__parahaemolyticus**	11	3	79	157	6.137E-01	1.1435	5.915E-01	7.180E-01
**k__Bacteria;p__Firmicutes;c__Clostridia;o__Clostridiales;f__Veillonellaceae;g__Veillonella;s__**	20	4	360	49	-4.887E-01	0.9295	5.991E-01	7.245E-01
**k__Bacteria;p__Actinobacteria;c__Actinobacteria;o__Actinomycetales;f__Actinomycetaceae;g__Varibaculum;s__**	16	3	152	14	-5.358E-01	1.0326	6.038E-01	7.250E-01
**k__Bacteria;p__Bacteroidetes;c__Bacteroidia;o__Bacteroidales;f__Prevotellaceae;g__Prevotella;s__**	96	23	11849	1386	2.256E-01	0.4324	6.018E-01	7.250E-01
**k__Bacteria;p__Actinobacteria;c__Actinobacteria;o__Actinomycetales;f__Gordoniaceae;g__Gordonia;s__**	5	3	108	46	6.405E-01	1.3193	6.274E-01	7.479E-01
**k__Bacteria;p__TM7;c__TM7-3;o__CW040;f__F16;g__;s__**	8	4	61	113	5.612E-01	1.1520	6.261E-01	7.479E-01
**k__Bacteria;p__Chloroflexi;c__Ellin6529;o__;f__;g__;s__**	7	6	27	48	4.028E-01	0.8726	6.444E-01	7.655E-01
**k__Bacteria;p__Firmicutes;c__Bacilli;o__Lactobacillales;f__Carnobacteriaceae;g__;s__**	19	10	84	103	2.647E-01	0.5963	6.571E-01	7.778E-01
**k__Bacteria;p__Cyanobacteria;c__Nostocophycideae;o__Stigonematales;f__Rivulariaceae;g__Calothrix;s__**	5	2	229	139	-7.036E-01	1.6023	6.606E-01	7.792E-01
**k__Bacteria;p__Proteobacteria;c__Alphaproteobacteria;o__Rhizobiales;f__Bradyrhizobiaceae;g__Bosea;s__genosp.**	14	6	56	38	3.520E-01	0.8166	6.664E-01	7.833E-01
**k__Bacteria;p__Proteobacteria;c__Alphaproteobacteria;o__Rhizobiales;f__Hyphomicrobiaceae;g__Devosia;s__**	35	11	242	220	2.380E-01	0.5613	6.715E-01	7.866E-01
**k__Bacteria;p__Proteobacteria;c__Betaproteobacteria;o__Burkholderiales;f__Alcaligenaceae;g__Achromobacter;s__**	89	20	948	304	-1.641E-01	0.4017	6.830E-01	7.972E-01
**k__Bacteria;p__Actinobacteria;c__Acidimicrobiia;o__Acidimicrobiales;f__Microthrixaceae;g__CandidatusMicrothrix;s__parvicella**	4	2	17	10	5.742E-01	1.4673	6.955	7.994

© 2021 Hsiao CJ et al. *JAMA Network Open.*

							E-01	E-01
**k__Bacteria;p__Proteobacteria;c__Alphaproteobacteria;o__Rhodobacterales;f__Rhodobacteraceae;g__Amaricoccus;s__**	3	4	8	39	5.541E-01	1.3874	6.896E-01	7.994E-01
**k__Bacteria;p__Proteobacteria;c__Gammaproteobacteria;o__Pseudomonadales;f__Moraxellaceae;g__Alkanindiges;s__**	4	3	12	42	5.130E-01	1.3327	7.003E-01	7.994E-01
**k__Bacteria;p__Proteobacteria;c__Alphaproteobacteria;o__Rhizobiales;f__Rhizobiaceae;g__Rhizobium;s__**	6	3	50	19	-4.721E-01	1.2366	7.027E-01	7.994E-01
**k__Bacteria;p__Bacteroidetes;c__Flavobacteriia;o__Flavobacteriales;f__[Weeksellaceae];g__Wautersiella;s__**	5	3	9	12	4.702E-01	1.2444	7.055E-01	7.994E-01
**k__Bacteria;p__Cyanobacteria;c__Oscillatoriophycideae;o__Chroococcales;f__Xenococcaceae;g__;s__**	20	5	794	795	-3.685E-01	0.9644	7.024E-01	7.994E-01
**k__Bacteria;p__Proteobacteria;c__Betaproteobacteria;o__Burkholderiales;f__Oxalobacteraceae;g__Cupriavidus;s__**	30	3	218	13	-3.607E-01	0.9573	7.063E-01	7.994E-01
**k__Bacteria;p__Bacteroidetes;c__Bacteroidia;o__Bacteroidales;f__Prevotellaceae;g__Prevotella;s__nigrescens**	20	5	103	72	2.958E-01	0.7808	7.048E-01	7.994E-01
**k__Bacteria;p__Actinobacteria;c__Coriobacteriia;o__Coriobacteriales;f__Coriobacteriaceae;g__Collinsella;s__aerofaciens**	21	7	187	203	2.798E-01	0.7304	7.016E-01	7.994E-01
**k__Bacteria;p__Proteobacteria;c__Gammaproteobacteria;o__Enterobacteriales;f__Enterobacteriaceae;g__;s__**	165	30	28143	171053	6.425E-01	1.8241	7.247E-01	8.146E-01
**k__Bacteria;p__Actinobacteria;c__Actinobacteria;o__Actinomycetales;f__Cellulomonadaceae;g__Cellulomonas;s__**	23	4	158	47	-3.230E-01	0.9103	7.228E-01	8.146E-01
**k__Bacteria;p__Proteobacteria;c__Gammaproteobacteria;o__Vibrionales;f__Vibrionaceae;g__Vibrio;s__**	13	2	44	9	-4.313E-01	1.2654	7.332E-01	8.215E-01
**k__Bacteria;p__Firmicutes;c__Bacilli;o__Lactobacillales;f__Aerococcaceae;g__Alloiococcus;s__**	87	16	245413	35744	2.721E-01	0.8266	7.420E-01	8.286E-01
**k__Bacteria;p__Firmicutes;c__Clostridia;o__Clostridiales;f__Lachnospiraceae;g__Blautia;s__**	38	11	349	289	1.766E-01	0.5588	7.520E-01	8.369E-01
**k__Bacteria;p__Firmicutes;c__Bacilli;o__Lactobacillales;f__Lactobacillaceae;g__Lactobacillus;s__**	89	15	2425	1389	1.463E-01	0.4858	7.633E-01	8.467E-01
**k__Bacteria;p__Proteobacteria;c__Betaproteobacteria;o__Burkholderiales;f__Comamonadaceae;g__Variovorax;s__paradoxus**	151	30	624	374	-6.074E-02	0.2055	7.676E-01	8.487E-01
**k__Bacteria;p__Cyanobacteria;c__Oscillatoriophycideae;o__Chroococcales;f__Xenococcaceae;g__Chroococcidiopsis;s__**	8	3	70	43	-3.276E-01	1.2000	7.849E-01	8.649E-01
**k__Bacteria;p__Actinobacteria;c__Actinobacteria;o__Actinomycetales;f__Dietziaceae;g__;s__**	5	3	26	14	3.368E-01	1.2699	7.908	8.684

© 2021 Hsiao CJ et al. *JAMA Network Open.*

							E-01	E-01
**k__Bacteria;p__Proteobacteria;c__Gammaproteobacteria;o__Pseudomonadales;f__Pseudomonadaceae;g__Pseudomonas;s__**	144	27	12292	1218	-8.047E-02	0.3069	7.932E-01	8.684E-01
**k__Bacteria;p__Proteobacteria;c__Alphaproteobacteria;o__Rhizobiales;f__Rhizobiaceae;g__;s__**	13	6	39	56	2.002E-01	0.7751	7.962E-01	8.688E-01
**k__Bacteria;p__Proteobacteria;c__Gammaproteobacteria;o__Pseudomonadales;f__Moraxellaceae;g__Psychrobacter;s__sanguinis**	14	2	36	15	-3.079E-01	1.2241	8.014E-01	8.712E-01
**k__Bacteria;p__Firmicutes;c__Clostridia;o__Clostridiales;f__Lachnospiraceae;g__Dorea;s__**	6	4	47	21	2.742E-01	1.1027	8.036E-01	8.712E-01
**k__Bacteria;p__Proteobacteria;c__Alphaproteobacteria;o__Caulobacterales;f__Caulobacteraceae;g__Phenylobacterium;s__**	6	5	50	14	-2.354E-01	0.9935	8.127E-01	8.782E-01
**k__Bacteria;p__Proteobacteria;c__Betaproteobacteria;o__Burkholderiales;f__Oxalobacteraceae;g__Janthinobacterium;s__lividum**	156	30	3010	1456	-6.666E-02	0.2853	8.153E-01	8.782E-01
**k__Bacteria;p__Actinobacteria;c__Actinobacteria;o__Bifidobacteriales;f__Bifidobacteriaceae;g__;s__**	40	6	256	281	1.537E-01	0.6982	8.257E-01	8.841E-01
**k__Bacteria;p__[Thermi];c__Deinococci;o__Thermales;f__Thermaceae;g__Thermus;s__**	67	11	1197	302	-1.229E-01	0.5593	8.260E-01	8.841E-01
**k__Bacteria;p__Actinobacteria;c__Actinobacteria;o__Actinomycetales;f__Brevibacteriaceae;g__Brevibacterium;s__**	40	9	1058	119	-1.380E-01	0.6615	8.347E-01	8.905E-01
**k__Bacteria;p__Firmicutes;c__Bacilli;o__Lactobacillales;f__Aerococcaceae;g__Aerococcus;s__**	15	3	151	14	-2.247E-01	1.1190	8.409E-01	8.941E-01
**k__Bacteria;p__Cyanobacteria;c__Chloroplast;o__Streptophyta;f__;g__;s__**	110	21	5634	1447	-7.896E-02	0.3996	8.434E-01	8.941E-01
**k__Bacteria;p__Proteobacteria;c__Gammaproteobacteria;o__Alteromonadales;f__Psychromonadaceae;g__Psychromonas;s__**	3	2	33	12	2.444E-01	1.5984	8.785E-01	9.172E-01
**k__Bacteria;p__Actinobacteria;c__Actinobacteria;o__Actinomycetales;f__Dietziaceae;g__Dietzia;s__**	10	2	287	5	-2.128E-01	1.4051	8.796E-01	9.172E-01
**k__Bacteria;p__Firmicutes;c__Erysipelotrichi;o__Erysipelotrichales;f__Erysipelotrichaceae;g__[Eubacterium];s__biforme**	4	3	8	12	-1.964E-01	1.3203	8.818E-01	9.172E-01
**k__Bacteria;p__Firmicutes;c__Bacilli;o__Lactobacillales;f__Streptococcaceae;g__Lactococcus;s__**	47	7	527	174	1.066E-01	0.7117	8.810E-01	9.172E-01
**k__Bacteria;p__Proteobacteria;c__Gammaproteobacteria;o__Enterobacteriales;f__Enterobacteriaceae;g__Erwinia;s__**	27	7	146	26	-1.065E-01	0.6633	8.725E-01	9.172E-01
**k__Bacteria;p__Actinobacteria;c__Actinobacteria;o__Actinomycetales;f__Micrococcaceae;g__Kocuria;s__rhizophila**	77	15	21509	2495	8.936E-02	0.5764	8.768	9.172

© 2021 Hsiao CJ et al. *JAMA Network Open.*

							E-01	E-01
**k__Bacteria;p__Proteobacteria;c__Epsilonproteobacteria;o__Campylobacterales;f__Campylobacteraceae;g__Campylobacter;s__**	69	14	4611	374	-8.045E-02	0.5530	8.843E-01	9.172E-01
**k__Bacteria;p__Actinobacteria;c__Actinobacteria;o__Actinomycetales;f__Actinomycetaceae;g__Actinobaculum;s__**	10	2	42	33	1.640E-01	1.2892	8.987E-01	9.293E-01
**k__Bacteria;p__Proteobacteria;c__Alphaproteobacteria;o__Sphingomonadales;f__Sphingomonadaceae;g__Sphingopyxis;s__alaskensis**	2	3	4	12	1.686E-01	1.6492	9.186E-01	9.398E-01
**k__Bacteria;p__Proteobacteria;c__Gammaproteobacteria;o__Alteromonadales;f__[Chromatiaceae];g__Rheinheimera;s__**	2	4	19	18	1.586E-01	1.4780	9.146E-01	9.398E-01
**k__Bacteria;p__Proteobacteria;c__Gammaproteobacteria;o__Pseudomonadales;f__Moraxellaceae;g__Psychrobacter;s__**	14	4	27	9	9.196E-02	0.9173	9.201E-01	9.398E-01
**k__Bacteria;p__Bacteroidetes;c__Bacteroidia;o__Bacteroidales;f__Bacteroidaceae;g__Bacteroides;s__**	30	9	471	314	6.942E-02	0.6382	9.134E-01	9.398E-01
**k__Bacteria;p__Bacteroidetes;c__Bacteroidia;o__Bacteroidales;f__Bacteroidaceae;g__Bacteroides;s__caccae**	5	2	23	32	1.235E-01	1.4600	9.326E-01	9.494E-01
**k__Bacteria;p__Firmicutes;c__Bacilli;o__Bacillales;f__Bacillaceae;g__Bacillus;s__flexus**	5	3	30	13	1.062E-01	1.3136	9.356E-01	9.494E-01
**k__Bacteria;p__Bacteroidetes;c__Cytophagia;o__Cytophagales;f__Cytophagaceae;g__Dyadobacter;s__**	3	4	38	43	-1.031E-01	1.3281	9.381E-01	9.494E-01
**k__Bacteria;p__Bacteroidetes;c__Flavobacteriia;o__Flavobacteriales;f__[Weeksellaceae];g__Cloacibacterium;s__**	44	9	349	236	3.330E-02	0.6085	9.564E-01	9.650E-01
**k__Bacteria;p__Firmicutes;c__Bacilli;o__Lactobacillales;f__Aerococcaceae;g__;s__**	18	5	72	33	3.569E-02	0.7851	9.637E-01	9.695E-01
**k__Bacteria;p__Actinobacteria;c__Actinobacteria;o__Actinomycetales;f__Nocardioidaceae;g__Propionicimonas;s__**	7	7	423	69	-3.987E-02	0.9883	9.678E-01	9.707E-01
**k__Bacteria;p__Bacteroidetes;c__Bacteroidia;o__Bacteroidales;f__Bacteroidaceae;g__Bacteroides;s__ovatus**	7	4	219	65	5.892E-03	1.2341	9.962E-01	9.962E-01
**k__Bacteria;p__[Thermi];c__Deinococci;o__Deinococcales;f__Deinococcaceae;g__CM44;s__**	1	2	1	115	NA	NA	NA	NA
**k__Bacteria;p__Acidobacteria;c__[Chloracidobacteria];o__DS-100;f__;g__;s__**	1	6	1	434	NA	NA	NA	NA
**k__Bacteria;p__Acidobacteria;c__[Chloracidobacteria];o__RB41;f__;g__;s__**	0	3	0	74	NA	NA	NA	NA
**k__Bacteria;p__Acidobacteria;c__Acidobacteria-6;o__CCU21;f__;g__;s__**	1	3	6	30	NA	NA	NA	NA
**k__Bacteria;p__Acidobacteria;c__Acidobacteria-6;o__iii1-15;f__mb2424;g__;s__**	1	5	10	111	NA	NA	NA	NA
**k__Bacteria;p__Acidobacteria;c__DA052;o__Ellin6513;f__;g__;s__**	2	0	65	0	NA	NA	NA	NA

© 2021 Hsiao CJ et al. *JAMA Network Open.*

k__Bacteria;p__Acidobacteria;c__Holophagae;o__Holophagales;f__Holophagaceae;g__Geothrix;s__	0	2	0	40	NA	NA	NA	NA
k__Bacteria;p__Acidobacteria;c__Sva0725;o__Sva0725;f__;g__;s__	0	6	0	60	NA	NA	NA	NA
k__Bacteria;p__Actinobacteria;c__Actinobacteria;o__Actinomycetales;f__ACK-M1;g__;s__	0	3	0	144	NA	NA	NA	NA
k__Bacteria;p__Actinobacteria;c__Actinobacteria;o__Actinomycetales;f__Actinomycetaceae;g__Mobiluncus;s__	7	1	133	1	NA	NA	NA	NA
k__Bacteria;p__Actinobacteria;c__Actinobacteria;o__Actinomycetales;f__Geodermatophilaceae;g__Geodermatophilus;s__	5	0	11	0	NA	NA	NA	NA
k__Bacteria;p__Actinobacteria;c__Actinobacteria;o__Actinomycetales;f__Intrasporangiaceae;g__Arsenicicoccus;s__	2	0	21	0	NA	NA	NA	NA
k__Bacteria;p__Actinobacteria;c__Actinobacteria;o__Actinomycetales;f__Kineosporiaceae;g__Kineococcus;s__	3	0	73	0	NA	NA	NA	NA
k__Bacteria;p__Actinobacteria;c__Actinobacteria;o__Actinomycetales;f__Micrococcaceae;g__Kocuria;s__	4	1	8	2	NA	NA	NA	NA
k__Bacteria;p__Actinobacteria;c__Actinobacteria;o__Actinomycetales;f__Micrococcaceae;g__Nesterenkonia;s__	0	2	0	40	NA	NA	NA	NA
k__Bacteria;p__Actinobacteria;c__Actinobacteria;o__Actinomycetales;f__Nocardiopsaceae;g__;s__	5	0	15	0	NA	NA	NA	NA
k__Bacteria;p__Actinobacteria;c__Actinobacteria;o__Actinomycetales;f__Propionibacteriaceae;g__Luteococcus;s__	2	1	22	1	NA	NA	NA	NA
k__Bacteria;p__Actinobacteria;c__Actinobacteria;o__Actinomycetales;f__Pseudonocardiaceae;g__Saccharopolyspora;s__	5	0	75	0	NA	NA	NA	NA
k__Bacteria;p__Actinobacteria;c__Actinobacteria;o__Actinomycetales;f__Streptomycetaceae;g__Streptomyces;s__	6	0	40	0	NA	NA	NA	NA
k__Bacteria;p__Actinobacteria;c__Actinobacteria;o__Actinomycetales;f__Williamsiaceae;g__Williamsia;s__	5	1	61	3	NA	NA	NA	NA
k__Bacteria;p__Actinobacteria;c__Coriobacteriia;o__Coriobacteriales;f__Coriobacteriaceae;g__Slackia;s__	0	2	0	37	NA	NA	NA	NA
k__Bacteria;p__Actinobacteria;c__KIST-JJY010;o__;f__;g__;s__	1	2	2	80	NA	NA	NA	NA
k__Bacteria;p__Actinobacteria;c__Rubrobacteria;o__Rubrobacterales;f__Rubrobacteraceae;g__Rubrobacter;s__	1	3	1	108	NA	NA	NA	NA
k__Bacteria;p__Actinobacteria;c__Thermoleophilia;o__Gaiellales;f__;g__;s__	1	5	1	243	NA	NA	NA	NA
k__Bacteria;p__Actinobacteria;c__Thermoleophilia;o__Gaiellales;f__AK1AB1_02E;g__;s__	1	4	8	79	NA	NA	NA	NA
k__Bacteria;p__Actinobacteria;c__Thermoleophilia;o__Gaiellales;f__Gaiellaceae;g__;s__	4	1	37	26	NA	NA	NA	NA
k__Bacteria;p__Aquificae;c__Aquificae;o__Aquificales;f__Aquificaceae;g__;s__	4	1	45	2	NA	NA	NA	NA
k__Bacteria;p__Armatimonadetes;c__[Fimbriimonadia];o__[Fimbriimonadales];f__[Fimbriimonadaceae];g__Fimbriimonas;s__	5	0	21	0	NA	NA	NA	NA
k__Bacteria;p__Bacteroidetes;c__[Rhodothermi];o__[Rhodothermales];f__Rhodothermaceae;g__Rubricoccus;s__	0	2	0	724	NA	NA	NA	NA
k__Bacteria;p__Bacteroidetes;c__[Saprospirae];o__[Saprospirales];f__Chitinophagaceae;g__Segetibacter;s__	1	2	29	39	NA	NA	NA	NA
k__Bacteria;p__Bacteroidetes;c__Bacteroidia;o__Bacteroidales;f__BA008;g__;s__	0	1	0	57	NA	NA	NA	NA
k__Bacteria;p__Bacteroidetes;c__Bacteroidia;o__Bacteroidales;f__Porphyromonadaceae;g__;s__	0	2	0	53	NA	NA	NA	NA
k__Bacteria;p__Bacteroidetes;c__Bacteroidia;o__Bacteroidales;f__Porphyromonadaceae;g__Dysgonomonas;s__	5	1	93	1	NA	NA	NA	NA
k__Bacteria;p__Bacteroidetes;c__Bacteroidia;o__Bacteroidales;f__Porphyromonadaceae;g__Tannerella;s__	3	0	43	0	NA	NA	NA	NA
k__Bacteria;p__Bacteroidetes;c__Bacteroidia;o__Bacteroidales;f__S24-7;g__;s__	1	0	21	0	NA	NA	NA	NA
k__Bacteria;p__Bacteroidetes;c__Cytophagia;o__Cytophagales;f__Cytophagaceae;g__;s__	1	3	69	48	NA	NA	NA	NA
k__Bacteria;p__Bacteroidetes;c__Cytophagia;o__Cytophagales;f__Cytophagaceae;g__Rhodocytophaga;s__	1	0	78	0	NA	NA	NA	NA

**k__Bacteria;p__Bacteroidetes;c__Cytophagia;o__Cytophagales;f__Cytophagaceae;g__Rudanella;s__**	1	2	1	474	NA	NA	NA	NA
**k__Bacteria;p__Bacteroidetes;c__Flavobacteriia;o__Flavobacteriales;f__Cryomorphaceae;g__Fluviicola;s__**	1	0	22	0	NA	NA	NA	NA
**k__Bacteria;p__Bacteroidetes;c__Flavobacteriia;o__Flavobacteriales;f__Flavobacteriaceae;g__;s__**	1	3	22	101	NA	NA	NA	NA
**k__Bacteria;p__Bacteroidetes;c__Flavobacteriia;o__Flavobacteriales;f__Flavobacteriaceae;g__Myroides;s__**	2	0	27	0	NA	NA	NA	NA
**k__Bacteria;p__Bacteroidetes;c__Sphingobacteriia;o__Sphingobacteriales;f__;g__;s__**	1	1	44	137	NA	NA	NA	NA
**k__Bacteria;p__Chlamydiae;c__Chlamydiia;o__Chlamydiales;f__Parachlamydiaceae;g__;s__**	1	6	1	181	NA	NA	NA	NA
**k__Bacteria;p__Chlamydiae;c__Chlamydiia;o__Chlamydiales;f__Parachlamydiaceae;g__CandidatusProtochlamydia;s__**	1	1	3	644	NA	NA	NA	NA
**k__Bacteria;p__Chlamydiae;c__Chlamydiia;o__Chlamydiales;f__Parachlamydiaceae;g__Parachlamydia;s__**	0	1	0	22	NA	NA	NA	NA
**k__Bacteria;p__Chlamydiae;c__Chlamydiia;o__Chlamydiales;f__Rhabdochlamydiaceae;g__CandidatusRhabdochlamydia;s__**	0	1	0	97	NA	NA	NA	NA
**k__Bacteria;p__Chlorobi;c__Ignavibacteria;o__Ignavibacteriales;f__Ignavibacteriaceae;g__;s__**	0	1	0	71	NA	NA	NA	NA
**k__Bacteria;p__Chloroflexi;c__;o__;f__;g__;s__**	0	6	0	34	NA	NA	NA	NA
**k__Bacteria;p__Chloroflexi;c__Anaerolineae;o__A31;f__S47;g__;s__**	0	4	0	49	NA	NA	NA	NA
**k__Bacteria;p__Chloroflexi;c__Anaerolineae;o__Anaerolineales;f__Anaerolinaceae;g__Anaerolinea;s__**	1	0	26	0	NA	NA	NA	NA
**k__Bacteria;p__Chloroflexi;c__Anaerolineae;o__Caldilineales;f__Caldilineaceae;g__;s__**	1	6	1	58	NA	NA	NA	NA
**k__Bacteria;p__Chloroflexi;c__Anaerolineae;o__Caldilineales;f__Caldilineaceae;g__Caldilinea;s__**	1	7	1	346	NA	NA	NA	NA
**k__Bacteria;p__Chloroflexi;c__Anaerolineae;o__H39;f__;g__;s__**	0	2	0	55	NA	NA	NA	NA
**k__Bacteria;p__Chloroflexi;c__Anaerolineae;o__SHA-20;f__;g__;s__**	0	2	0	93	NA	NA	NA	NA
**k__Bacteria;p__Chloroflexi;c__Chloroflexi;o__[Roseiflexales];f__[Kouleothrixaceae];g__Kouleothrix;s__**	0	1	0	52	NA	NA	NA	NA
**k__Bacteria;p__Chloroflexi;c__Chloroflexi;o__AKIW781;f__;g__;s__**	2	0	40	0	NA	NA	NA	NA
**k__Bacteria;p__Chloroflexi;c__TK10;o__AKYG885;f__Dolo_23;g__;s__**	0	5	0	63	NA	NA	NA	NA
**k__Bacteria;p__Chloroflexi;c__TK17;o__mle1-48;f__;g__;s__**	0	4	0	88	NA	NA	NA	NA
**k__Bacteria;p__Cyanobacteria;c__4C0d-2;o__SM1D11;f__;g__;s__**	0	1	0	71	NA	NA	NA	NA
**k__Bacteria;p__Cyanobacteria;c__4C0d-2;o__YS2;f__;g__;s__**	1	5	1	117	NA	NA	NA	NA
**k__Bacteria;p__Cyanobacteria;c__Chloroplast;o__Chlorophyta;f__Chlamydomonadaceae;g__;s__**	0	2	0	74	NA	NA	NA	NA
**k__Bacteria;p__Cyanobacteria;c__Oscillatoriophycideae;o__Chroococcales;f__;g__;s__**	0	2	0	93	NA	NA	NA	NA
**k__Bacteria;p__Cyanobacteria;c__Oscillatoriophycideae;o__Chroococcales;f__Cyanobacteriaceae;g__Cyanobacterium;s__**	0	1	0	24	NA	NA	NA	NA
**k__Bacteria;p__Cyanobacteria;c__Oscillatoriophycideae;o__Oscillatoriales;f__Phormidiaceae;g__Phormidium;s__**	2	0	326	0	NA	NA	NA	NA
**k__Bacteria;p__Cyanobacteria;c__Synechococcophycideae;o__Pseudanabaenales;f__;g__;s__**	1	0	29	0	NA	NA	NA	NA
**k__Bacteria;p__Firmicutes;c__;o__;f__;g__;s__**	2	0	26	0	NA	NA	NA	NA
**k__Bacteria;p__Firmicutes;c__Bacilli;o__Bacillales;f__Bacillaceae;g__;s__**	11	1	75	77	NA	NA	NA	NA
**k__Bacteria;p__Firmicutes;c__Bacilli;o__Bacillales;f__Listeriaceae;g__Brochothrix;s__**	9	0	64	0	NA	NA	NA	NA

© 2021 Hsiao CJ et al. *JAMA Network Open.*

**k__Bacteria;p__Firmicutes;c__Bacilli;o__Bacillales;f__Paenibacillaceae;g__Aneurinibacillus;s__**	1	1	1	31	NA	NA	NA	NA
**k__Bacteria;p__Firmicutes;c__Bacilli;o__Bacillales;f__Planococcaceae;g__Sporosarcina;s__**	4	1	5	4	NA	NA	NA	NA
**k__Bacteria;p__Firmicutes;c__Bacilli;o__Bacillales;f__Staphylococcaceae;g__Salinicoccus;s__**	2	0	27	0	NA	NA	NA	NA
**k__Bacteria;p__Firmicutes;c__Bacilli;o__Gemellales;f__;g__;s__**	16	0	32	0	NA	NA	NA	NA
**k__Bacteria;p__Firmicutes;c__Bacilli;o__Lactobacillales;f__;g__;s__**	13	1	46	1	NA	NA	NA	NA
**k__Bacteria;p__Firmicutes;c__Bacilli;o__Lactobacillales;f__Aerococcaceae;g__Alkalibacterium;s__**	7	0	294	0	NA	NA	NA	NA
**k__Bacteria;p__Firmicutes;c__Bacilli;o__Lactobacillales;f__Carnobacteriaceae;g__Trichococcus;s__**	1	0	37	0	NA	NA	NA	NA
**k__Bacteria;p__Firmicutes;c__Bacilli;o__Lactobacillales;f__Enterococcaceae;g__Vagococcus;s__**	5	0	6	0	NA	NA	NA	NA
**k__Bacteria;p__Firmicutes;c__Bacilli;o__Lactobacillales;f__Lactobacillaceae;g__;s__**	6	0	390	0	NA	NA	NA	NA
**k__Bacteria;p__Firmicutes;c__Bacilli;o__Lactobacillales;f__Lactobacillaceae;g__Pediococcus;s__**	1	0	238	0	NA	NA	NA	NA
**k__Bacteria;p__Firmicutes;c__Bacilli;o__Lactobacillales;f__Leuconostocaceae;g__Leuconostoc;s__**	13	1	80	1	NA	NA	NA	NA
**k__Bacteria;p__Firmicutes;c__Bacilli;o__Lactobacillales;f__Leuconostocaceae;g__Weissella;s__**	4	1	9	3	NA	NA	NA	NA
**k__Bacteria;p__Firmicutes;c__Bacilli;o__Lactobacillales;f__Streptococcaceae;g__;s__**	1	2	1	46	NA	NA	NA	NA
**k__Bacteria;p__Firmicutes;c__Clostridia;o__Clostridiales;f__[Tissierellaceae];g__;s__**	3	0	35	0	NA	NA	NA	NA
**k__Bacteria;p__Firmicutes;c__Clostridia;o__Clostridiales;f__[Tissierellaceae];g__Helcococcus;s__**	5	1	54	2	NA	NA	NA	NA
**k__Bacteria;p__Firmicutes;c__Clostridia;o__Clostridiales;f__[Tissierellaceae];g__ph2;s__**	13	0	103	0	NA	NA	NA	NA
**k__Bacteria;p__Firmicutes;c__Clostridia;o__Clostridiales;f__Christensenellaceae;g__;s__**	1	0	24	0	NA	NA	NA	NA
**k__Bacteria;p__Firmicutes;c__Clostridia;o__Clostridiales;f__Eubacteriaceae;g__Acetobacterium;s__**	0	6	0	33	NA	NA	NA	NA
**k__Bacteria;p__Firmicutes;c__Clostridia;o__Clostridiales;f__Eubacteriaceae;g__Pseudoramibacter_Eubacterium;s__**	0	4	0	88	NA	NA	NA	NA
**k__Bacteria;p__Firmicutes;c__Clostridia;o__Clostridiales;f__Lachnospiraceae;g__Shuttleworthia;s__**	9	0	37	0	NA	NA	NA	NA
**k__Bacteria;p__Firmicutes;c__Clostridia;o__Clostridiales;f__Veillonellaceae;g__;s__**	0	3	0	11 4	NA	NA	NA	NA
**k__Bacteria;p__Firmicutes;c__Clostridia;o__Clostridiales;f__Veillonellaceae;g__Anaeromusa;s__**	0	3	0	41	NA	NA	NA	NA
**k__Bacteria;p__Firmicutes;c__Clostridia;o__Clostridiales;f__Veillonellaceae;g__Schwartzia;s__**	1	1	5	69	NA	NA	NA	NA
**k__Bacteria;p__Firmicutes;c__Clostridia;o__Clostridiales;f__Veillonellaceae;g__vadinHB04;s__**	0	1	0	34	NA	NA	NA	NA
**k__Bacteria;p__Fusobacteria;c__Fusobacteriia;o__Fusobacteriales;f__;g__;s__**	1	1	1	87	NA	NA	NA	NA
**k__Bacteria;p__Fusobacteria;c__Fusobacteriia;o__Fusobacteriales;f__Fusobacteriaceae;g__u114;s__**	0	5	0	17	NA	NA	NA	NA
**k__Bacteria;p__Gemmatimonadetes;c__Gemm-1;o__;f__;g__;s__**	0	1	0	43	NA	NA	NA	NA
**k__Bacteria;p__Gemmatimonadetes;c__Gemm-3;o__;f__;g__;s__**	2	0	48	0	NA	NA	NA	NA
**k__Bacteria;p__Gemmatimonadetes;c__Gemmatimonadetes;o__;f__;g__;s__**	1	0	18	0	NA	NA	NA	NA
**k__Bacteria;p__Gemmatimonadetes;c__Gemmatimonadetes;o__Gemmatimonadales;f__;g__;s__**	0	2	0	15 3	NA	NA	NA	NA
**k__Bacteria;p__GN02;c__BD1-5;o__;f__;g__;s__**	1	1	1	69	NA	NA	NA	NA

© 2021 Hsiao CJ et al. *JAMA Network Open.*

**k__Bacteria;p__GN02;c__GKS2-174;o__;f__;g__;s__**	0	1	0	26	NA	NA	NA	NA
**k__Bacteria;p__Kazan-3B-28;c__;o__;f__;g__;s__**	0	2	0	36	NA	NA	NA	NA
**k__Bacteria;p__Nitrospirae;c__Nitrospira;o__Nitrospirales;f__Nitrospiraceae;g__Nitrospira;s__**	1	3	1	447	NA	NA	NA	NA
**k__Bacteria;p__OD1;c__;o__;f__;g__;s__**	0	5	0	95	NA	NA	NA	NA
**k__Bacteria;p__OD1;c__ABY1;o__;f__;g__;s__**	0	1	0	41	NA	NA	NA	NA
**k__Bacteria;p__OD1;c__SM2F11;o__;f__;g__;s__**	0	1	0	176	NA	NA	NA	NA
**k__Bacteria;p__OP11;c__WCHB1-64;o__d153;f__;g__;s__**	1	1	19	30	NA	NA	NA	NA
**k__Bacteria;p__Planctomycetes;c__C6;o__d113;f__;g__;s__**	0	1	0	34	NA	NA	NA	NA
**k__Bacteria;p__Planctomycetes;c__Phycisphaerae;o__Phycisphaerales;f__;g__;s__**	1	0	25	0	NA	NA	NA	NA
**k__Bacteria;p__Planctomycetes;c__Phycisphaerae;o__WD2101;f__;g__;s__**	2	0	22	0	NA	NA	NA	NA
**k__Bacteria;p__Planctomycetes;c__Planctomycetia;o__B97;f__;g__;s__**	1	5	1	73	NA	NA	NA	NA
**k__Bacteria;p__Planctomycetes;c__Planctomycetia;o__Gemmatales;f__Gemmataceae;g__;s__**	0	3	0	299	NA	NA	NA	NA
**k__Bacteria;p__Proteobacteria;c__Alphaproteobacteria;o__BD7-3;f__;g__;s__**	0	2	0	145	NA	NA	NA	NA
**k__Bacteria;p__Proteobacteria;c__Alphaproteobacteria;o__Caulobacterales;f__Caulobacteraceae;g__Arthrospira;s__**	4	1	5	4	NA	NA	NA	NA
**k__Bacteria;p__Proteobacteria;c__Alphaproteobacteria;o__Rhizobiales;f__Bradyrhizobiaceae;g__Balneimonas;s__**	7	0	23	0	NA	NA	NA	NA
**k__Bacteria;p__Proteobacteria;c__Alphaproteobacteria;o__Rhizobiales;f__Brucellaceae;g__Ochrobactrum;s__**	15	1	68	2	NA	NA	NA	NA
**k__Bacteria;p__Proteobacteria;c__Alphaproteobacteria;o__Rhizobiales;f__Hyphomicrobiaceae;g__Parvibaculum;s__**	1	0	71	0	NA	NA	NA	NA
**k__Bacteria;p__Proteobacteria;c__Alphaproteobacteria;o__Rhizobiales;f__Hyphomicrobiaceae;g__Pedomicrobium;s__**	0	3	0	49	NA	NA	NA	NA
**k__Bacteria;p__Proteobacteria;c__Alphaproteobacteria;o__Rhizobiales;f__Methylocystaceae;g__;s__**	5	0	36	0	NA	NA	NA	NA
**k__Bacteria;p__Proteobacteria;c__Alphaproteobacteria;o__Rhodobacterales;f__Rhodobacteraceae;g__Jannaschia;s__**	0	2	0	32	NA	NA	NA	NA
**k__Bacteria;p__Proteobacteria;c__Alphaproteobacteria;o__Rhodobacterales;f__Rhodobacteraceae;g__Rhodobacter;s__**	1	6	1	57	NA	NA	NA	NA
**k__Bacteria;p__Proteobacteria;c__Alphaproteobacteria;o__Rhodospirillales;f__Acetobacteraceae;g__Roseococcus;s__**	1	3	1	110	NA	NA	NA	NA
**k__Bacteria;p__Proteobacteria;c__Alphaproteobacteria;o__Rhodospirillales;f__Rhodospirillaceae;g__Azospirillum;s__**	1	3	2	71	NA	NA	NA	NA
**k__Bacteria;p__Proteobacteria;c__Alphaproteobacteria;o__Rickettsiales;f__mitochondria;g__;s__**	17	0	555	0	NA	NA	NA	NA
**k__Bacteria;p__Proteobacteria;c__Alphaproteobacteria;o__Rickettsiales;f__Rickettsiaceae;g__;s__**	1	5	1	131	NA	NA	NA	NA
**k__Bacteria;p__Proteobacteria;c__Alphaproteobacteria;o__Sphingomonadales;f__Sphingomonadaceae;g__Zymomonas;s__**	0	3	0	40	NA	NA	NA	NA
**k__Bacteria;p__Proteobacteria;c__Betaproteobacteria;o__;f__;g__;s__**	0	1	0	40	NA	NA	NA	NA
**k__Bacteria;p__Proteobacteria;c__Betaproteobacteria;o__ASSO-13;f__;g__;s__**	1	4	1	103	NA	NA	NA	NA
**k__Bacteria;p__Proteobacteria;c__Betaproteobacteria;o__Burkholderiales;f__;g__;s__**	0	3	0	64	NA	NA	NA	NA
**k__Bacteria;p__Proteobacteria;c__Betaproteobacteria;o__Burkholderiales;f__Burkholderiaceae;g__;s__**	0	2	0	33	NA	NA	NA	NA
**k__Bacteria;p__Proteobacteria;c__Betaproteobacteria;o__Burkholderiales;f__Comamonadaceae;g__Variovorax;s__**	5	0	9	0	NA	NA	NA	NA

© 2021 Hsiao CJ et al. *JAMA Network Open.*

**k__Bacteria;p__Proteobacteria;c__Betaproteobacteria;o__Ellin6067;f__;g__;s__**	0	3	0	23	NA	NA	NA	NA
**k__Bacteria;p__Proteobacteria;c__Betaproteobacteria;o__Hydrogenophilales;f__Hydrogenophilaceae;g__Thiobacillus;s__**	9	1	43	7	NA	NA	NA	NA
**k__Bacteria;p__Proteobacteria;c__Betaproteobacteria;o__Neisseriales;f__Neisseriaceae;g__Microvirgula;s__**	0	2	0	42	NA	NA	NA	NA
**k__Bacteria;p__Proteobacteria;c__Betaproteobacteria;o__Nitrosomonadales;f__Nitrosomonadaceae;g__;s__**	1	4	1	145	NA	NA	NA	NA
**k__Bacteria;p__Proteobacteria;c__Betaproteobacteria;o__Procabacteriales;f__Procabacteriaceae;g__;s__**	1	4	1	377	NA	NA	NA	NA
**k__Bacteria;p__Proteobacteria;c__Betaproteobacteria;o__Rhodocyclales;f__Rhodocyclaceae;g__C39;s__**	0	3	0	51	NA	NA	NA	NA
**k__Bacteria;p__Proteobacteria;c__Betaproteobacteria;o__Rhodocyclales;f__Rhodocyclaceae;g__Hydrogenophilus;s__**	9	0	61	0	NA	NA	NA	NA
**k__Bacteria;p__Proteobacteria;c__Betaproteobacteria;o__SC-I-84;f__;g__;s__**	0	6	0	232	NA	NA	NA	NA
**k__Bacteria;p__Proteobacteria;c__Deltaproteobacteria;o__;f__;g__;s__**	0	2	0	143	NA	NA	NA	NA
**k__Bacteria;p__Proteobacteria;c__Deltaproteobacteria;o__Bdellovibrionales;f__Bacteriovoracaceae;g__;s__**	4	1	64	73	NA	NA	NA	NA
**k__Bacteria;p__Proteobacteria;c__Deltaproteobacteria;o__Desulfobacterales;f__Desulfobulbaceae;g__Desulfobulbus;s__**	1	2	11	65	NA	NA	NA	NA
**k__Bacteria;p__Proteobacteria;c__Deltaproteobacteria;o__FAC87;f__;g__;s__**	0	1	0	38	NA	NA	NA	NA
**k__Bacteria;p__Proteobacteria;c__Deltaproteobacteria;o__MIZ46;f__;g__;s__**	0	1	0	30	NA	NA	NA	NA
**k__Bacteria;p__Proteobacteria;c__Deltaproteobacteria;o__Myxococcales;f__OM27;g__;s__**	3	0	20	0	NA	NA	NA	NA
**k__Bacteria;p__Proteobacteria;c__Deltaproteobacteria;o__Myxococcales;f__Polyangiaceae;g__;s__**	0	2	0	137	NA	NA	NA	NA
**k__Bacteria;p__Proteobacteria;c__Deltaproteobacteria;o__NB1-j;f__JTB38;g__;s__**	0	1	0	37	NA	NA	NA	NA
**k__Bacteria;p__Proteobacteria;c__Deltaproteobacteria;o__NKB15;f__;g__;s__**	0	3	0	28	NA	NA	NA	NA
**k__Bacteria;p__Proteobacteria;c__Deltaproteobacteria;o__Spirobacillales;f__;g__;s__**	0	3	0	1918	NA	NA	NA	NA
**k__Bacteria;p__Proteobacteria;c__Deltaproteobacteria;o__Syntrophobacterales;f__Syntrophaceae;g__Desulfomonile;s__**	0	3	0	39	NA	NA	NA	NA
**k__Bacteria;p__Proteobacteria;c__Deltaproteobacteria;o__Syntrophobacterales;f__Syntrophorhabdaceae;g__;s__**	0	1	0	23	NA	NA	NA	NA
**k__Bacteria;p__Proteobacteria;c__Epsilonproteobacteria;o__Campylobacterales;f__Campylobacteraceae;g__Sulfurospirillum;s__**	0	3	0	42	NA	NA	NA	NA
**k__Bacteria;p__Proteobacteria;c__Epsilonproteobacteria;o__Campylobacterales;f__Helicobacteraceae;g__;s__**	1	2	1	24	NA	NA	NA	NA
**k__Bacteria;p__Proteobacteria;c__Gammaproteobacteria;o__;f__;g__;s__**	4	0	27	0	NA	NA	NA	NA
**k__Bacteria;p__Proteobacteria;c__Gammaproteobacteria;o__Aeromonadales;f__Aeromonadaceae;g__Tolumonas;s__**	1	2	7	194	NA	NA	NA	NA
**k__Bacteria;p__Proteobacteria;c__Gammaproteobacteria;o__Alteromonadales;f__[Chromatiaceae];g__;s__**	6	0	9	0	NA	NA	NA	NA
**k__Bacteria;p__Proteobacteria;c__Gammaproteobacteria;o__Alteromonadales;f__OM60;g__;s__**	0	1	0	21	NA	NA	NA	NA
**k__Bacteria;p__Proteobacteria;c__Gammaproteobacteria;o__Chromatiales;f__Chromatiaceae;g__Allochromatium;s__**	0	1	0	116	NA	NA	NA	NA
**k__Bacteria;p__Proteobacteria;c__Gammaproteobacteria;o__Chromatiales;f__Halothiobacillaceae;g__Thiovirga;s__**	0	1	0	123	NA	NA	NA	NA
**k__Bacteria;p__Proteobacteria;c__Gammaproteobacteria;o__Enterobacteriales;f__Enterobacteriaceae;g__Buchnera;s__**	1	0	29	0	NA	NA	NA	NA
**k__Bacteria;p__Proteobacteria;c__Gammaproteobacteria;o__Enterobacteriales;f__Enterobacteriaceae;g__Citrobacter;s__**	10	0	26	0	NA	NA	NA	NA
**k__Bacteria;p__Proteobacteria;c__Gammaproteobacteria;o__Enterobacteriales;f__Enterobacteriaceae;g__Yersinia;s__**	6	0	9	0	NA	NA	NA	NA

© 2021 Hsiao CJ et al. *JAMA Network Open.*

**k__Bacteria;p__Proteobacteria;c__Gammaproteobacteria;o__HOC36;f__;g__;s__**	0	2	0	888	NA	NA	NA	NA
**k__Bacteria;p__Proteobacteria;c__Gammaproteobacteria;o__Legionellales;f__;g__;s__**	1	7	5	1471	NA	NA	NA	NA
**k__Bacteria;p__Proteobacteria;c__Gammaproteobacteria;o__Legionellales;f__Coxiellaceae;g__;s__**	0	5	0	326	NA	NA	NA	NA
**k__Bacteria;p__Proteobacteria;c__Gammaproteobacteria;o__Legionellales;f__Coxiellaceae;g__Aquicella;s__**	1	7	6	1922	NA	NA	NA	NA
**k__Bacteria;p__Proteobacteria;c__Gammaproteobacteria;o__Legionellales;f__Francisellaceae;g__;s__**	0	1	0	23	NA	NA	NA	NA
**k__Bacteria;p__Proteobacteria;c__Gammaproteobacteria;o__Legionellales;f__Legionellaceae;g__Legionella;s__**	1	5	1	479	NA	NA	NA	NA
**k__Bacteria;p__Proteobacteria;c__Gammaproteobacteria;o__Legionellales;f__Legionellaceae;g__Tatlockia;s__**	0	1	0	32	NA	NA	NA	NA
**k__Bacteria;p__Proteobacteria;c__Gammaproteobacteria;o__Methylococcales;f__Methylococcaceae;g__Methylocaldum;s__**	0	2	0	197	NA	NA	NA	NA
**k__Bacteria;p__Proteobacteria;c__Gammaproteobacteria;o__Oceanospirillales;f__Halomonadaceae;g__;s__**	1	0	62	0	NA	NA	NA	NA
**k__Bacteria;p__Proteobacteria;c__Gammaproteobacteria;o__Thiotrichales;f__Thiotrichaceae;g__Thiothrix;s__**	0	3	0	37	NA	NA	NA	NA
**k__Bacteria;p__Proteobacteria;c__Gammaproteobacteria;o__Vibrionales;f__Vibrionaceae;g__Photobacterium;s__**	5	0	73	0	NA	NA	NA	NA
**k__Bacteria;p__Proteobacteria;c__Gammaproteobacteria;o__Xanthomonadales;f__Xanthomonadaceae;g__Luteimonas;s__**	3	0	35	0	NA	NA	NA	NA
**k__Bacteria;p__Spirochaetes;c__Spirochaetes;o__Sphaerochaetales;f__Sphaerochaetaceae;g__Sphaerochaeta;s__**	0	2	0	140	NA	NA	NA	NA
**k__Bacteria;p__TM6;c__SJA-4;o__S1198;f__;g__;s__**	0	4	0	112	NA	NA	NA	NA
**k__Bacteria;p__TM7;c__TM7-3;o__CW040;f__;g__;s__**	6	1	99	2	NA	NA	NA	NA
**k__Bacteria;p__TM7;c__TM7-3;o__I025;f__;g__;s__**	1	1	1	185	NA	NA	NA	NA
**k__Bacteria;p__Verrucomicrobia;c__[Spartobacteria];o__[Chthoniobacterales];f__[Chthoniobacteraceae];g__DA101;s__**	1	0	47	0	NA	NA	NA	NA
**k__Bacteria;p__WS3;c__PRR-12;o__Sediment-1;f__PRR-10;g__;s__**	0	1	0	23	NA	NA	NA	NA
**k__Bacteria;p__WS6;c__SC72;o__WCHB1-15;f__;g__;s__**	0	3	0	29	NA	NA	NA	NA
**k__Bacteria;p__Chlamydiae;c__Chlamydiia;o__Chlamydiales;f__Parachlamydiaceae;g__Parachlamydia;s__acanthamoebae**	0	2	0	133	NA	NA	NA	NA
**k__Bacteria;p__Firmicutes;c__Clostridia;o__Clostridiales;f__Peptostreptococcaceae;g__Peptostreptococcus;s__anaerobius**	5	0	17	0	NA	NA	NA	NA
**k__Bacteria;p__Synergistetes;c__Synergistia;o__Synergistales;f__Dethiosulfovibrionaceae;g__Jonquetella;s__anthropi**	1	0	65	0	NA	NA	NA	NA
**k__Bacteria;p__Actinobacteria;c__Actinobacteria;o__Actinomycetales;f__Mycobacteriaceae;g__Mycobacterium;s__arupense**	1	3	1	25	NA	NA	NA	NA
**k__Bacteria;p__Proteobacteria;c__Deltaproteobacteria;o__Bdellovibrionales;f__Bdellovibrionaceae;g__Bdellovibrio;s__bacteriovorus**	0	1	0	78	NA	NA	NA	NA
**k__Bacteria;p__Actinobacteria;c__Actinobacteria;o__Actinomycetales;f__Corynebacteriaceae;g__Corynebacterium;s__bovis**	2	0	22	0	NA	NA	NA	NA
**k__Bacteria;p__Proteobacteria;c__Gammaproteobacteria;o__Xanthomonadales;f__Xanthomonadaceae;g__Lysobacter;s__brunescens**	0	2	0	104	NA	NA	NA	NA
**k__Bacteria;p__Proteobacteria;c__Betaproteobacteria;o__Neisseriales;f__Neisseriaceae;g__Neisseria;s__cinerea**	1	2	10	39	NA	NA	NA	NA
**k__Bacteria;p__Proteobacteria;c__Betaproteobacteria;o__Burkholderiales;f__Oxalobacteraceae;g__Polynucleobacter;s__cosmopolitanus**	0	2	0	139	NA	NA	NA	NA
**k__Bacteria;p__Bacteroidetes;c__Bacteroidia;o__Bacteroidales;f__Porphyromonadaceae;g__Parabacteroides;s__distasonis**	5	1	15	7	NA	NA	NA	NA
**k__Bacteria;p__Bacteroidetes;c__Bacteroidia;o__Bacteroidales;f__Porphyromonadaceae;g__Porphyromonas;s__endodontalis**	10	1	42	2	NA	NA	NA	NA
**k__Bacteria;p__Actinobacteria;c__Actinobacteria;o__Actinomycetales;f__Actinomycetaceae;g__Actinomyces;s__europaeus**	4	1	74	15	NA	NA	NA	NA

© 2021 Hsiao CJ et al. *JAMA Network Open.*

**k__Bacteria;p__Bacteroidetes;c__Flavobacteriia;o__Flavobacteriales;f__Flavobacteriaceae;g__Flavobacterium;s__frigidarium**	6	0	7	0	NA	NA	NA	NA
**k__Bacteria;p__Proteobacteria;c__Gammaproteobacteria;o__Pasteurellales;f__Pasteurellaceae;g__Haemophilus;s__influenzae**	6	0	748	0	NA	NA	NA	NA
**k__Bacteria;p__Actinobacteria;c__Actinobacteria;o__Actinomycetales;f__Microbacteriaceae;g__Agrococcus;s__jenensis**	9	1	19	14	NA	NA	NA	NA
**k__Bacteria;p__Proteobacteria;c__Alphaproteobacteria;o__Rhizobiales;f__Rhizobiaceae;g__Rhizobium;s__leguminosarum**	13	0	333	0	NA	NA	NA	NA
**k__Bacteria;p__Firmicutes;c__Bacilli;o__Lactobacillales;f__Leuconostocaceae;g__Leuconostoc;s__mesenteroides**	7	1	18	68	NA	NA	NA	NA
**k__Bacteria;p__Proteobacteria;c__Betaproteobacteria;o__Methylophilales;f__Methylophilaceae;g__Methylotenera;s__mobilis**	1	6	3	535	NA	NA	NA	NA
**k__Bacteria;p__Verrucomicrobia;c__Verrucomicrobiae;o__Verrucomicrobiales;f__Verrucomicrobiaceae;g__Akkermansia;s__muciniphila**	1	4	1	9	NA	NA	NA	NA
**k__Bacteria;p__Bacteroidetes;c__Sphingobacteriia;o__Sphingobacteriales;f__Sphingobacteriaceae;g__Sphingobacterium;s__multivorum**	15	1	130	4	NA	NA	NA	NA
**k__Bacteria;p__Firmicutes;c__Bacilli;o__Lactobacillales;f__Aerococcaceae;g__Alloiococcus;s__otitis**	6	0	46	0	NA	NA	NA	NA
**k__Bacteria;p__Actinobacteria;c__Actinobacteria;o__Actinomycetales;f__Micrococcaceae;g__Kocuria;s__palustris**	5	1	8	2	NA	NA	NA	NA
**k__Bacteria;p__Firmicutes;c__Bacilli;o__Bacillales;f__Staphylococcaceae;g__Staphylococcus;s__pettenkoferi**	6	0	15	0	NA	NA	NA	NA
**k__Bacteria;p__Synergistetes;c__Synergistia;o__Synergistales;f__Dethiosulfovibrionaceae;g__Pyramidobacter;s__piscolens**	0	1	0	70	NA	NA	NA	NA
**k__Bacteria;p__Actinobacteria;c__Actinobacteria;o__Actinomycetales;f__Micrococcaceae;g__Arthrobacter;s__psychrolactophilus**	5	1	22	2	NA	NA	NA	NA
**k__Bacteria;p__Firmicutes;c__Bacilli;o__Bacillales;f__Staphylococcaceae;g__Jeotgalicoccus;s__psychrophilus**	8	0	33	0	NA	NA	NA	NA
**k__Bacteria;p__Firmicutes;c__Bacilli;o__Lactobacillales;f__Aerococcaceae;g__Marinilactibacillus;s__psychrotolerans**	2	0	44	0	NA	NA	NA	NA
**k__Bacteria;p__Proteobacteria;c__Gammaproteobacteria;o__Pseudomonadales;f__Moraxellaceae;g__Acinetobacter;s__rhizosphaerae**	29	1	184	2	NA	NA	NA	NA
**k__Bacteria;p__Actinobacteria;c__Actinobacteria;o__Actinomycetales;f__Micrococcaceae;g__Microbispora;s__rosea**	10	1	76	91	NA	NA	NA	NA
**k__Bacteria;p__Proteobacteria;c__Gammaproteobacteria;o__Pseudomonadales;f__Moraxellaceae;g__Acinetobacter;s__schindleri**	5	0	193	0	NA	NA	NA	NA
**k__Bacteria;p__Firmicutes;c__Bacilli;o__Bacillales;f__Staphylococcaceae;g__Staphylococcus;s__sciuri**	11	1	43	79	NA	NA	NA	NA
**k__Bacteria;p__Firmicutes;c__Bacilli;o__Bacillales;f__Bacillaceae;g__Bacillus;s__selenatarsenatis**	8	0	85	0	NA	NA	NA	NA
**k__Bacteria;p__Firmicutes;c__Bacilli;o__Lactobacillales;f__Streptococcaceae;g__Streptococcus;s__sobrinus**	2	1	72	1	NA	NA	NA	NA
**k__Bacteria;p__Spirochaetes;c__Spirochaetes;o__Spirochaetales;f__Spirochaetaceae;g__Treponema;s__socranskii**	1	2	1	33	NA	NA	NA	NA
**k__Bacteria;p__Bacteroidetes;c__Flavobacteriia;o__Flavobacteriales;f__Flavobacteriaceae;g__Flavobacterium;s__succinicans**	10	1	17	2	NA	NA	NA	NA
**k__Bacteria;p__Actinobacteria;c__Actinobacteria;o__Actinomycetales;f__Corynebacteriaceae;g__Corynebacterium;s__variabile**	15	1	102	1	NA	NA	NA	NA
**k__Bacteria;p__Proteobacteria;c__Alphaproteobacteria;o__Sphingomonadales;f__Sphingomonadaceae;g__Sphingomonas;s__wittichii**	5	0	111	0	NA	NA	NA	NA
**k__Bacteria;p__Proteobacteria;c__Alphaproteobacteria;o__Sphingomonadales;f__Sphingomonadaceae;g__Sphingomonas;s__yabuuchiae**	3	1	22	30	NA	NA	NA	NA

© 2021 Hsiao CJ et al. *JAMA Network Open.*

eTable 2. Association of Taxa Abundance With Composite Infectious Outcome

taxa	rank cluster	rank infection	infection q-value	infection OR	infection lower 95	infection upper 95
k__Bacteria;p__Proteobacteria;c__Gammaproteobacteria;o__Pasteurellales;f__Pasteurellaceae;g__Aggregatibacter;s__segnis	177	1	1.148 E-03	1.610	1.336	1.986
k__Bacteria;p__Proteobacteria;c__Gammaproteobacteria;o__Pseudomonadales;f__Moraxellaceae;g__Moraxella;s__	19	2	1.302 E-03	1.162	1.091	1.240
k__Bacteria;p__Proteobacteria;c__Alphaproteobacteria;o__;f__;g__;s__	269	3	4.397 E-03	0.741	0.641	0.848
k__Bacteria;p__Proteobacteria;c__Gammaproteobacteria;o__Enterobacteriales;f__Enterobacteriaceae;g__Salmonella;s__	111	4	5.636 E-03	1.626	1.305	2.086
k__Bacteria;p__Firmicutes;c__Bacilli;o__Bacillales;f__Staphylococcaceae;g__Jeotgalicoccus;s__	142	5	1.055 E-02	1.320	1.151	1.530
k__Bacteria;p__Actinobacteria;c__Actinobacteria;o__Actinomycetales;f__Propionibacteriaceae;g__;s__	189	6	1.055 E-02	1.417	1.190	1.702
k__Bacteria;p__Bacteroidetes;c__Bacteroidia;o__Bacteroidales;f__[Paraprevotellaceae];g__[Prevotella];s__	153	7	1.055 E-02	1.269	1.124	1.437
k__Bacteria;p__Proteobacteria;c__Alphaproteobacteria;o__Rhizobiales;f__Rhizobiaceae;g__;s__	307	8	1.284 E-02	0.454	0.284	0.659
k__Bacteria;p__Proteobacteria;c__Betaproteobacteria;o__Burkholderiales;f__Comamonadaceae;g__Variovorax;s__paradoxus	303	9	1.284 E-02	1.410	1.182	1.703
k__Bacteria;p__Firmicutes;c__Clostridia;o__Clostridiales;f__Peptococcaceae;g__Peptococcus;s__	131	10	1.302 E-02	1.594	1.255	2.081
k__Bacteria;p__Actinobacteria;c__Actinobacteria;o__Actinomycetales;f__Propionibacteriaceae;g__Propionibacterium;s__acnes	253	11	1.302 E-02	0.818	0.732	0.910
k__Bacteria;p__Proteobacteria;c__Betaproteobacteria;o__Burkholderiales;f__Oxalobacteraceae;g__Janthinobacterium;s__lividum	311	12	1.302 E-02	1.277	1.121	1.461
k__Bacteria;p__Proteobacteria;c__Gammaproteobacteria;o__Pasteurellales;f__Pasteurellaceae;g__Haemophilus;s__	119	13	1.328 E-02	1.158	1.072	1.258
k__Bacteria;p__Actinobacteria;c__Actinobacteria;o__Actinomycetales;f__Propionibacteriaceae;g__Propionibacterium;s__granulosum	51	14	1.557 E-02	0.706	0.580	0.854
k__Bacteria;p__Firmicutes;c__Clostridia;o__Clostridiales;f__Ruminococcaceae;g__;s__	93	15	1.629 E-02	0.774	0.666	0.888
k__Bacteria;p__Bacteroidetes;c__Flavobacteriia;o__Flavobacteriales;f__Flavobacteriaceae;g__Capnocytophaga;s__ochracea	197	16	1.629 E-02	2.563	1.603	4.809
k__Bacteria;p__Firmicutes;c__Clostridia;o__Clostridiales;f__[Tissierellaceae];g__Finegoldia;s__	132	17	1.629 E-02	0.866	0.798	0.939
k__Bacteria;p__Proteobacteria;c__Gammaproteobacteria;o__Enterobacteriales;f__Enterobacteriaceae;g__Morganella;s__	194	18	1.629 E-02	0.743	0.622	0.871
k__Bacteria;p__Cyanobacteria;c__Chloroplast;o__Streptophyta;f__;g__;s__	316	19	1.931 E-02	1.160	1.066	1.265
k__Bacteria;p__Proteobacteria;c__Alphaproteobacteria;o__Sphingomonadales;f__Sphingomonadaceae;g__Novosphingobium;s__	33	20	2.017 E-02	0.719	0.586	0.863
k__Bacteria;p__Firmicutes;c__Clostridia;o__Clostridiales;f__[Tissierellaceae];g__Anaerococcus;s__	56	21	2.017 E-02	0.864	0.792	0.940
k__Bacteria;p__Proteobacteria;c__Alphaproteobacteria;o__Rhizobiales;f__Bradyrhizobiaceae;g__Bosea;s__genosp.	285	22	2.067 E-02	0.607	0.440	0.794
k__Bacteria;p__Proteobacteria;c__Gammaproteobacteria;o__Xanthomonadales;f__Xanthomonadaceae;g__;s__	231	23	2.091 E-02	0.839	0.755	0.929
k__Bacteria;p__Actinobacteria;c__Actinobacteria;o__Actinomycetales;f__Brevibacteriaceae;g__Brevibacterium;s__	314	24	2.298 E-02	1.236	1.090	1.405
k__Bacteria;p__Actinobacteria;c__Actinobacteria;o__Actinomycetales;f__Propionibacteriaceae;g__Propionibacterium;s__	18	25	2.298 E-02	0.862	0.788	0.941
k__Bacteria;p__Proteobacteria;c__Deltaproteobacteria;o__Desulfobacterales;f__Desulfobulbaceae;g__Desulfobulbus;s__	476	26	2.395 E-02	2.068	1.466	3.868
k__Bacteria;p__Actinobacteria;c__Actinobacteria;o__Actinomycetales;f__Geodermatophilaceae;g__Modestobacter;s__	263	27	2.484 E-02	0.484	0.290	0.714
k__Bacteria;p__Proteobacteria;c__Alphaproteobacteria;o__Rhizobiales;f__Phyllobacteriaceae;g__Phyllobacterium;s__	274	28	2.484 E-02	1.285	1.105	1.502
k__Bacteria;p__Actinobacteria;c__Actinobacteria;o__Actinomycetales;f__Actinomycetaceae;g__;s__	184	29	2.484 E-02	0.449	0.252	0.686

© 2021 Hsiao CJ et al. *JAMA Network Open.*

k__Bacteria;p__Firmicutes;c__Bacilli;o__Lactobacillales;f__Aerococcaceae;g__Facklamia;s__	179	30	2.861 E-02	1.252	1.090	1.440
k__Bacteria;p__Firmicutes;c__Bacilli;o__Lactobacillales;f__Aerococcaceae;g__Aerococcus;s__	315	31	2.960 E-02	0.565	0.376	0.777
k__Bacteria;p__Fusobacteria;c__Fusobacteriia;o__Fusobacteriales;f__Fusobacteriaceae;g__u114;s__	429	32	2.960 E-02	0.266	0.098	0.541
k__Bacteria;p__Proteobacteria;c__Gammaproteobacteria;o__Pasteurellales;f__Pasteurellaceae;g__Actinobacillus;s__parahaemolyticus	276	33	3.403 E-02	1.435	1.156	1.839
k__Bacteria;p__Actinobacteria;c__Actinobacteria;o__Bifidobacteriales;f__Bifidobacteriaceae;g__Scardovia;s__	145	34	3.784 E-02	1.361	1.126	1.684
k__Bacteria;p__Actinobacteria;c__Coriobacteriia;o__Coriobacteriales;f__Coriobacteriaceae;g__Atopobium;s__	40	35	3.794 E-02	1.190	1.064	1.332
k__Bacteria;p__Synergistetes;c__Synergistia;o__Synergistales;f__Synergistaceae;g__vadinCA02;s__	165	36	3.866 E-02	0.341	0.131	0.596
k__Bacteria;p__Firmicutes;c__Clostridia;o__Clostridiales;f__Lachnospiraceae;g__Catonella;s__	139	37	3.866 E-02	1.415	1.148	1.822
k__Bacteria;p__Proteobacteria;c__Alphaproteobacteria;o__Rhodobacterales;f__Rhodobacteraceae;g__Paracoccus;s__	160	38	4.210 E-02	1.211	1.068	1.375
k__Bacteria;p__Firmicutes;c__Erysipelotrichi;o__Erysipelotrichales;f__Erysipelotrichaceae;g__Bulleidia;s__	76	39	4.210 E-02	1.261	1.086	1.479
k__Bacteria;p__Firmicutes;c__Bacilli;o__Bacillales;f__Staphylococcaceae;g__Staphylococcus;s__aureus	239	40	4.244 E-02	0.834	0.738	0.939
k__Bacteria;p__Firmicutes;c__Clostridia;o__Clostridiales;f__[Tissierellaceae];g__Parvimonas;s__	16	41	4.453 E-02	1.168	1.053	1.296
k__Bacteria;p__Proteobacteria;c__Gammaproteobacteria;o__Alteromonadales;f__Shewanellaceae;g__Shewanella;s__	260	42	4.794 E-02	0.618	0.432	0.834
k__Bacteria;p__Firmicutes;c__Bacilli;o__Lactobacillales;f__;g__;s__	408	43	4.833 E-02	0.281	0.099	0.577
k__Bacteria;p__Firmicutes;c__Bacilli;o__Bacillales;f__Planococcaceae;g__Sporosarcina;s__	405	44	5.180 E-02	2.036	1.337	3.718
k__Bacteria;p__Firmicutes;c__Bacilli;o__Bacillales;f__Staphylococcaceae;g__Staphylococcus;s__sciuri	546	45	5.379 E-02	1.501	1.170	2.089
k__Bacteria;p__Chloroflexi;c__Anaerolineae;o__A31;f__S47;g__;s__	384	46	5.837 E-02	1.761	1.216	2.730
k__Bacteria;p__Actinobacteria;c__Actinobacteria;o__Actinomycetales;f__Micrococcaceae;g__Kocuria;s__	349	47	5.837 E-02	2.655	1.450	6.058
k__Bacteria;p__Firmicutes;c__Bacilli;o__Bacillales;f__Bacillaceae;g__;s__	402	48	5.837 E-02	1.459	1.148	1.981
k__Bacteria;p__SR1;c__;o__;f__;g__;s__	247	49	6.181 E-02	1.430	1.116	1.862
k__Bacteria;p__Actinobacteria;c__Actinobacteria;o__Actinomycetales;f__Mycobacteriaceae;g__Mycobacterium;s__arupense	519	50	6.181 E-02	2.526	1.406	5.334
k__Bacteria;p__Spirochaetes;c__Spirochaetes;o__Spirochaetales;f__Spirochaetaceae;g__Treponema;s__socranskii	549	51	6.181 E-02	1.622	1.195	2.451
k__Bacteria;p__Proteobacteria;c__Betaproteobacteria;o__Burkholderiales;f__Comamonadaceae;g__Hydrogenophaga;s__	147	52	6.340 E-02	0.678	0.495	0.868
k__Bacteria;p__Bacteroidetes;c__Sphingobacteriia;o__Sphingobacteriales;f__Sphingobacteriaceae;g__Sphingobacterium;s__	20	53	6.436 E-02	0.630	0.420	0.837
k__Bacteria;p__Proteobacteria;c__Alphaproteobacteria;o__Sphingomonadales;f__Sphingomonadaceae;g__;s__	35	54	6.436 E-02	0.845	0.745	0.950
k__Bacteria;p__Firmicutes;c__Clostridia;o__Clostridiales;f__[Tissierellaceae];g__Gallicola;s__	181	55	6.436 E-02	1.280	1.078	1.548
k__Bacteria;p__Actinobacteria;c__Actinobacteria;o__Actinomycetales;f__Propionibacteriaceae;g__Microlunatus;s__	262	56	6.436 E-02	0.589	0.384	0.835
k__Bacteria;p__Proteobacteria;c__Alphaproteobacteria;o__Rhodospirillales;f__;g__;s__	101	57	6.788 E-02	1.351	1.089	1.695
k__Bacteria;p__Firmicutes;c__Clostridia;o__Clostridiales;f__Veillonellaceae;g__Phascolarctobacterium;s__	257	58	7.066 E-02	0.379	0.159	0.679
k__Bacteria;p__Actinobacteria;c__Actinobacteria;o__Actinomycetales;f__Micrococcaceae;g__Kocuria;s__palustris	537	59	7.110 E-02	2.285	1.304	4.584
k__Bacteria;p__Actinobacteria;c__Actinobacteria;o__Actinomycetales;f__Micrococcaceae;g__Microbispora;s__rosea	544	60	7.110 E-02	1.394	1.112	1.842

k__Bacteria;p__Actinobacteria;c__Coriobacteriia;o__Coriobacteriales;f__Coriobacteriaceae;g__Slackia;s__	356	61	7.137 E-02	1.885	1.298	3.609
k__Bacteria;p__Bacteroidetes;c__Bacteroidia;o__Bacteroidales;f__Prevotellaceae;g__Prevotella;s__copri	243	62	7.287 E-02	0.665	0.473	0.875
k__Bacteria;p__Proteobacteria;c__Gammaproteobacteria;o__Pseudomonadales;f__Moraxellaceae;g__Acinetobacter;s__	208	63	8.260 E-02	0.841	0.737	0.958
k__Bacteria;p__Planctomycetes;c__Planctomycetia;o__Gemmatales;f__Gemmataceae;g__;s__	446	64	8.548 E-02	1.469	1.117	2.027
k__Bacteria;p__Proteobacteria;c__Betaproteobacteria;o__Burkholderiales;f__Comamonadaceae;g__Limnohabitans;s__	135	65	1.003 E-01	0.759	0.602	0.929
k__Bacteria;p__Actinobacteria;c__Actinobacteria;o__Actinomycetales;f__Corynebacteriaceae;g__Corynebacterium;s__durum	183	66	1.015 E-01	1.170	1.035	1.325
k__Bacteria;p__Bacteroidetes;c__Bacteroidia;o__Bacteroidales;f__Prevotellaceae;g__Prevotella;s__nanceiensis	174	67	1.029 E-01	1.256	1.050	1.505
k__Bacteria;p__Bacteroidetes;c__Bacteroidia;o__Bacteroidales;f__Prevotellaceae;g__Prevotella;s__intermedia	171	68	1.029 E-01	1.310	1.068	1.650
k__Bacteria;p__Firmicutes;c__Clostridia;o__Clostridiales;f__Eubacteriaceae;g__Pseudoramibacter_Eubacterium;s__	422	69	1.034 E-01	1.366	1.084	1.803
k__Bacteria;p__Actinobacteria;c__Actinobacteria;o__Actinomycetales;f__Nocardioidaceae;g__Propionicimonas;s__	334	70	1.140 E-01	0.615	0.398	0.872
k__Bacteria;p__Proteobacteria;c__Betaproteobacteria;o__Burkholderiales;f__Burkholderiaceae;g__Burkholderia;s__	198	71	1.143 E-01	1.164	1.031	1.318
k__Bacteria;p__Proteobacteria;c__Gammaproteobacteria;o__Enterobacteriales;f__Enterobacteriaceae;g__;s__	297	72	1.172 E-01	1.135	1.026	1.260
k__Bacteria;p__Proteobacteria;c__Betaproteobacteria;o__ASSO-13;f__;g__;s__	462	73	1.197 E-01	0.542	0.303	0.837
k__Bacteria;p__Firmicutes;c__Clostridia;o__Clostridiales;f__[Tissierellaceae];g__Helcococcus;s__	418	74	1.197 E-01	1.732	1.150	2.873
k__Bacteria;p__Actinobacteria;c__Actinobacteria;o__Actinomycetales;f__Nocardiaceae;g__Rhodococcus;s__fascians	275	75	1.201 E-01	0.476	0.238	0.820
k__Bacteria;p__Firmicutes;c__Clostridia;o__Clostridiales;f__Lachnospiraceae;g__[Ruminococcus];s__	190	76	1.201 E-01	0.720	0.539	0.928
k__Bacteria;p__Actinobacteria;c__Thermoleophilia;o__Solirubrobacterales;f__Solirubrobacteraceae;g__;s__	180	77	1.213 E-01	0.456	0.191	0.763
k__Bacteria;p__Acidobacteria;c__[Chloracidobacteria];o__DS-100;f__;g__;s__	337	78	1.216 E-01	1.249	1.042	1.508
k__Bacteria;p__Proteobacteria;c__Alphaproteobacteria;o__Rhizobiales;f__Rhizobiaceae;g__Agrobacterium;s__	81	79	1.216 E-01	0.875	0.780	0.974
k__Bacteria;p__Proteobacteria;c__Alphaproteobacteria;o__Sphingomonadales;f__Sphingomonadaceae;g__Sphingomonas;s__echinoides	129	80	1.216 E-01	0.878	0.786	0.975
k__Bacteria;p__Actinobacteria;c__Actinobacteria;o__Bifidobacteriales;f__Bifidobacteriaceae;g__Bifidobacterium;s__longum	36	81	1.216 E-01	0.804	0.661	0.952
k__Bacteria;p__Proteobacteria;c__Alphaproteobacteria;o__Rickettsiales;f__Rickettsiaceae;g__;s__	459	82	1.216 E-01	0.632	0.410	0.891
k__Bacteria;p__Proteobacteria;c__Gammaproteobacteria;o__Pseudomonadales;f__Moraxellaceae;g__Psychrobacter;s__	327	83	1.216 E-01	0.629	0.403	0.887
k__Bacteria;p__[Thermi];c__Deinococci;o__Thermales;f__Thermaceae;g__Thermus;s__	313	84	1.278 E-01	0.870	0.772	0.976
k__Bacteria;p__Firmicutes;c__Bacilli;o__Lactobacillales;f__Carnobacteriaceae;g__Carnobacterium;s__	125	85	1.356 E-01	0.761	0.592	0.945
k__Bacteria;p__Proteobacteria;c__Alphaproteobacteria;o__Sphingomonadales;f__Sphingomonadaceae;g__Sphingopyxis;s__	71	86	1.356 E-01	1.253	1.037	1.529
k__Bacteria;p__Firmicutes;c__Bacilli;o__Lactobacillales;f__Streptococcaceae;g__Streptococcus;s__anginosus	114	87	1.396 E-01	1.143	1.019	1.283
k__Bacteria;p__Actinobacteria;c__Actinobacteria;o__Actinomycetales;f__Yaniellaceae;g__Yaniella;s__	215	88	1.485 E-01	1.529	1.071	2.278
k__Bacteria;p__Cyanobacteria;c__Oscillatoriophycideae;o__Chroococcales;f__Xenococcaceae;g__;s__	293	89	1.508 E-01	1.159	1.019	1.320
k__Bacteria;p__Firmicutes;c__Clostridia;o__Clostridiales;f__Veillonellaceae;g__Megamonas;s__	137	90	1.515 E-01	0.581	0.284	0.831
k__Bacteria;p__Actinobacteria;c__Rubrobacteria;o__Rubrobacterales;f__Rubrobacteraceae;g__Rubrobacter;s__	358	91	1.515 E-01	1.325	1.038	1.710

© 2021 Hsiao CJ et al. *JAMA Network Open.*

**k__Bacteria;p__Proteobacteria;c__Betaproteobacteria;o__Burkholderiales;f__Comamonadaceae;g__Delftia;s__**	170	92	1.515 E-01	1.121	1.016	1.242
**k__Bacteria;p__Proteobacteria;c__Alphaproteobacteria;o__Rhizobiales;f__;g__;s__**	2	93	1.558 E-01	1.113	1.013	1.224
**k__Bacteria;p__Firmicutes;c__Clostridia;o__Clostridiales;f__Lachnospiraceae;g__[Ruminococcus];s__gnavus**	217	94	1.580 E-01	1.427	1.078	2.069
**k__Bacteria;p__Proteobacteria;c__Betaproteobacteria;o__Nitrosomonadales;f__Nitrosomonadaceae;g__;s__**	469	95	1.598 E-01	1.501	1.074	2.268
**k__Bacteria;p__Proteobacteria;c__Gammaproteobacteria;o__Pseudomonadales;f__Pseudomonadaceae;g__Pseudomonas;s__viridiflava**	220	96	1.617 E-01	0.889	0.798	0.985
**k__Bacteria;p__Firmicutes;c__Bacilli;o__Lactobacillales;f__Lactobacillaceae;g__Lactobacillus;s__**	302	97	1.617 E-01	1.125	1.013	1.251
**k__Bacteria;p__Firmicutes;c__Bacilli;o__Lactobacillales;f__Leuconostocaceae;g__Leuconostoc;s__mesenteroides**	532	98	1.686 E-01	1.491	1.087	2.336
**k__Bacteria;p__Proteobacteria;c__Alphaproteobacteria;o__Sphingomonadales;f__Sphingomonadaceae;g__Kaistobacter;s__**	113	99	1.722 E-01	0.864	0.754	0.983
**k__Bacteria;p__Firmicutes;c__Bacilli;o__Bacillales;f__;g__;s__**	58	100	1.796 E-01	0.845	0.718	0.980
**k__Bacteria;p__Kazan-3B-28;c__;o__;f__;g__;s__**	436	101	1.796 E-01	0.744	0.545	0.951
**k__Bacteria;p__Proteobacteria;c__Betaproteobacteria;o__Neisseriales;f__Neisseriaceae;g__Neisseria;s__subflava**	228	102	1.894 E-01	1.367	1.026	1.857
**k__Bacteria;p__TM7;c__TM7-3;o__EW055;f__;g__;s__**	134	103	1.894 E-01	0.844	0.712	0.980
**k__Bacteria;p__Firmicutes;c__Clostridia;o__Clostridiales;f__Lachnospiraceae;g__Dorea;s__**	309	104	1.911 E-01	1.338	1.022	1.775
**k__Bacteria;p__Firmicutes;c__Clostridia;o__Clostridiales;f__Veillonellaceae;g__Veillonella;s__**	277	105	1.981 E-01	1.194	1.010	1.415
**k__Bacteria;p__Actinobacteria;c__Actinobacteria;o__Actinomycetales;f__Actinomycetaceae;g__Actinomyces;s__europaeus**	527	106	1.981 E-01	1.330	1.025	1.784
**k__Bacteria;p__Actinobacteria;c__Thermoleophilia;o__Gaiellales;f__Gaiellaceae;g__;s__**	361	107	2.070 E-01	1.550	1.065	2.613
**k__Bacteria;p__Actinobacteria;c__Thermoleophilia;o__Gaiellales;f__AK1AB1_02E;g__;s__**	360	108	2.070 E-01	1.417	1.021	2.017
**k__Bacteria;p__Firmicutes;c__Bacilli;o__Bacillales;f__Staphylococcaceae;g__Staphylococcus;s__**	202	109	2.105 E-01	0.884	0.784	0.995
**k__Bacteria;p__Proteobacteria;c__Gammaproteobacteria;o__Pasteurellales;f__Pasteurellaceae;g__Aggregatibacter;s__**	196	110	2.110 E-01	1.186	1.005	1.399
**k__Bacteria;p__Actinobacteria;c__Actinobacteria;o__Actinomycetales;f__Micrococcaceae;g__Nesterenkonia;s__**	350	111	2.145 E-01	1.244	1.010	1.557
**k__Bacteria;p__Proteobacteria;c__Betaproteobacteria;o__Rhodocyclales;f__Rhodocyclaceae;g__Zoogloea;s__**	128	112	2.158 E-01	1.288	1.009	1.664
**k__Bacteria;p__Proteobacteria;c__Betaproteobacteria;o__Ellin6067;f__;g__;s__**	466	113	2.158 E-01	3.223	1.340	12.749
**k__Bacteria;p__Actinobacteria;c__Actinobacteria;o__Actinomycetales;f__Micrococcaceae;g__Micrococcus;s__luteus**	169	114	2.220 E-01	1.277	1.004	1.630
**k__Bacteria;p__Proteobacteria;c__Gammaproteobacteria;o__Enterobacteriales;f__Enterobacteriaceae;g__Erwinia;s__**	321	115	2.244 E-01	0.779	0.599	0.984
**k__Bacteria;p__Proteobacteria;c__Alphaproteobacteria;o__Caulobacterales;f__Caulobacteraceae;g__Phenylobacterium;s__**	310	116	2.251 E-01	0.556	0.270	0.905
**k__Bacteria;p__Firmicutes;c__Bacilli;o__Lactobacillales;f__Lactobacillaceae;g__Lactobacillus;s__zeae**	158	117	2.258 E-01	1.390	1.022	2.018
**k__Bacteria;p__Firmicutes;c__Bacilli;o__Bacillales;f__Planococcaceae;g__;s__**	221	118	2.258 E-01	0.893	0.798	0.999
**k__Bacteria;p__Actinobacteria;c__Actinobacteria;o__Actinomycetales;f__Dermabacteraceae;g__Brachybacterium;s__conglomeratum**	265	119	2.293 E-01	0.833	0.687	0.993
**k__Bacteria;p__Firmicutes;c__Clostridia;o__Clostridiales;f__[Mogibacteriaceae];g__;s__**	205	120	2.293 E-01	1.222	1.001	1.502
**k__Bacteria;p__Bacteroidetes;c__Bacteroidia;o__Bacteroidales;f__Porphyromonadaceae;g__Dysgonomonas;s__**	368	121	2.322 E-01	1.912	1.076	4.045
**k__Bacteria;p__Actinobacteria;c__Actinobacteria;o__Actinomycetales;f__Microbacteriaceae;g__Microbacterium;s__**	23	122	2.322 E-01	0.910	0.825	0.999

© 2021 Hsiao CJ et al. *JAMA Network Open.*

**k__Bacteria;p__Actinobacteria;c__Actinobacteria;o__Actinomycetales;f__Dietziaceae;g__;s__**	305	123	2.322 E-01	0.595	0.295	0.903
**k__Bacteria;p__Chloroflexi;c__;o__;f__;g__;s__**	383	124	2.322 E-01	1.402	0.997	1.988
**k__Bacteria;p__Firmicutes;c__Bacilli;o__Lactobacillales;f__Leuconostocaceae;g__Leuconostoc;s__**	414	125	2.334 E-01	0.286	0.047	0.715
**k__Bacteria;p__Proteobacteria;c__Alphaproteobacteria;o__Sphingomonadales;f__Sphingomonadaceae;g__Zymomonas;s__**	460	126	2.350 E-01	1.427	0.997	2.090
**k__Bacteria;p__Proteobacteria;c__Betaproteobacteria;o__Burkholderiales;f__Comamonadaceae;g__Comamonas;s__**	216	127	2.350 E-01	0.808	0.638	0.991
**k__Bacteria;p__Actinobacteria;c__Actinobacteria;o__Actinomycetales;f__Dermabacteraceae;g__Dermabacter;s__**	244	128	2.350 E-01	0.828	0.676	0.996
**k__Bacteria;p__Firmicutes;c__Erysipelotrichi;o__Erysipelotrichales;f__Erysipelotrichaceae;g__[Eubacterium];s__biforme**	319	129	2.390 E-01	0.437	0.146	0.886
**k__Bacteria;p__Proteobacteria;c__Alphaproteobacteria;o__Sphingomonadales;f__Sphingomonadaceae;g__Sphingomonas;s__**	57	130	2.390 E-01	0.920	0.844	1.001
**k__Bacteria;p__Proteobacteria;c__Betaproteobacteria;o__Burkholderiales;f__Comamonadaceae;g__Acidovorax;s__**	199	131	2.459 E-01	1.308	0.997	1.766
**k__Bacteria;p__Firmicutes;c__Bacilli;o__Bacillales;f__Staphylococcaceae;g__Staphylococcus;s__epidermidis**	214	132	2.532 E-01	0.870	0.751	1.005
**k__Bacteria;p__Actinobacteria;c__Actinobacteria;o__Actinomycetales;f__Microbacteriaceae;g__Rathayibacter;s__caricis**	192	133	2.633 E-01	0.841	0.688	0.997
**k__Bacteria;p__Proteobacteria;c__Gammaproteobacteria;o__Xanthomonadales;f__Xanthomonadaceae;g__Stenotrophomonas;s__**	55	134	2.663 E-01	0.825	0.670	1.010
**k__Bacteria;p__Proteobacteria;c__Gammaproteobacteria;o__Pseudomonadales;f__Moraxellaceae;g__Psychrobacter;s__sanguinis**	308	135	2.663 E-01	0.604	0.313	0.958
**k__Bacteria;p__Actinobacteria;c__Actinobacteria;o__Actinomycetales;f__Actinomycetaceae;g__Actinobaculum;s__**	324	136	2.668 E-01	1.285	0.987	1.703
**k__Bacteria;p__Bacteroidetes;c__Flavobacteriia;o__Flavobacteriales;f__[Weeksellaceae];g__Elizabethkingia;s__meningoseptica**	154	137	2.703 E-01	0.833	0.675	1.002
**k__Bacteria;p__Firmicutes;c__Clostridia;o__Clostridiales;f__[Mogibacteriaceae];g__Mogibacterium;s__**	271	138	2.703 E-01	1.427	0.975	2.120
**k__Bacteria;p__Bacteroidetes;c__Bacteroidia;o__Bacteroidales;f__Porphyromonadaceae;g__Porphyromonas;s__endodontalis**	526	139	2.726 E-01	1.508	0.979	2.430
**k__Bacteria;p__Firmicutes;c__Bacilli;o__Gemellales;f__;g__;s__**	407	140	2.732 E-01	0.468	0.167	0.923
**k__Bacteria;p__Bacteroidetes;c__Flavobacteriia;o__Flavobacteriales;f__[Weeksellaceae];g__;s__**	148	141	2.745 E-01	1.183	0.986	1.425
**k__Bacteria;p__Acidobacteria;c__Acidobacteria-6;o__CCU21;f__;g__;s__**	339	142	2.755 E-01	1.549	0.989	2.671
**k__Bacteria;p__Proteobacteria;c__Gammaproteobacteria;o__Cardiobacteriales;f__Cardiobacteriaceae;g__Cardiobacterium;s__**	241	143	2.780 E-01	0.426	0.106	0.852
**k__Bacteria;p__Actinobacteria;c__Actinobacteria;o__Actinomycetales;f__Microbacteriaceae;g__;s__**	9	144	2.904 E-01	0.925	0.846	1.005
**k__Bacteria;p__Bacteroidetes;c__Bacteroidia;o__Bacteroidales;f__[Paraprevotellaceae];g__[Prevotella];s__tannerae**	259	145	2.909 E-01	1.193	0.981	1.458
**k__Bacteria;p__Proteobacteria;c__Gammaproteobacteria;o__Pseudomonadales;f__Pseudomonadaceae;g__;s__**	83	146	2.981 E-01	0.832	0.676	1.020
**k__Bacteria;p__Proteobacteria;c__Deltaproteobacteria;o__Syntrophobacterales;f__Syntrophobacteraceae;g__;s__**	175	147	2.986 E-01	1.332	0.969	1.863
**k__Bacteria;p__Bacteroidetes;c__Bacteroidia;o__Bacteroidales;f__Prevotellaceae;g__Prevotella;s__nigrescens**	295	148	3.009 E-01	1.194	0.977	1.461
**k__Bacteria;p__Proteobacteria;c__Betaproteobacteria;o__Burkholderiales;f__Alcaligenaceae;g__Achromobacter;s__**	287	149	3.070 E-01	0.910	0.817	1.012
**k__Bacteria;p__Proteobacteria;c__Gammaproteobacteria;o__Pseudomonadales;f__Pseudomonadaceae;g__Pseudomonas;s__fragi**	157	150	3.070 E-01	1.093	0.989	1.209
**k__Bacteria;p__Chloroflexi;c__Thermomicrobia;o__JG30-KF-CM45;f__;g__;s__**	3	151	3.141 E-01	1.081	0.988	1.182
**k__Bacteria;p__Proteobacteria;c__Alphaproteobacteria;o__Rhizobiales;f__Hyphomicrobiaceae;g__;s__**	236	152	3.168 E-01	1.261	0.964	1.652
**k__Bacteria;p__Verrucomicrobia;c__[Spartobacteria];o__[Chthoniobacterales];f__[Chthoniobacteraceae];g__CandidatusXiphinematobacter;s__**	151	153	3.168 E-01	0.868	0.726	1.011

© 2021 Hsiao CJ et al. *JAMA Network Open.*

**k__Bacteria;p__Proteobacteria;c__Gammaproteobacteria;o__Enterobacteriales;f__Enterobacteriaceae;g__Klebsiella;s__**	242	154	3.186 E-01	0.665	0.352	0.970
**k__Bacteria;p__Actinobacteria;c__Coriobacteriia;o__Coriobacteriales;f__Coriobacteriaceae;g__Collinsella;s__aerofaciens**	296	155	3.198 E-01	0.843	0.683	1.018
**k__Bacteria;p__Proteobacteria;c__Epsilonproteobacteria;o__Campylobacterales;f__Campylobacteraceae;g__Campylobacter;s__**	323	156	3.296 E-01	0.916	0.826	1.013
**k__Bacteria;p__[Thermi];c__Deinococci;o__Deinococcales;f__Deinococcaceae;g__Deinococcus;s__**	22	157	3.296 E-01	1.073	0.988	1.165
**k__Bacteria;p__WPS-2;c__;o__;f__;g__;s__**	240	158	3.296 E-01	0.799	0.594	1.017
**k__Bacteria;p__Bacteroidetes;c__Cytophagia;o__Cytophagales;f__Cytophagaceae;g__;s__**	371	159	3.296 E-01	1.264	0.959	1.680
**k__Bacteria;p__Verrucomicrobia;c__Verrucomicrobiae;o__Verrucomicrobiales;f__Verrucomicrobiaceae;g__Akkermansia;s__muciniphila**	534	160	3.296 E-01	0.572	0.279	1.060
**k__Bacteria;p__Synergistetes;c__Synergistia;o__Synergistales;f__Dethiosulfovibrionaceae;g__TG5;s__**	62	161	3.296 E-01	1.182	0.971	1.452
**k__Bacteria;p__Proteobacteria;c__Gammaproteobacteria;o__Thiotrichales;f__Thiotrichaceae;g__Thiothrix;s__**	506	162	3.394 E-01	0.815	0.625	1.024
**k__Bacteria;p__Bacteroidetes;c__Bacteroidia;o__Bacteroidales;f__Prevotellaceae;g__Prevotella;s__melaninogenica**	82	163	3.425 E-01	1.073	0.986	1.167
**k__Bacteria;p__Chloroflexi;c__Anaerolineae;o__Caldilineales;f__Caldilineaceae;g__;s__**	386	164	3.436 E-01	1.254	0.954	1.645
**k__Bacteria;p__Proteobacteria;c__Gammaproteobacteria;o__Pseudomonadales;f__Moraxellaceae;g__;s__**	52	165	3.441 E-01	1.072	0.986	1.166
**k__Bacteria;p__Actinobacteria;c__Actinobacteria;o__Actinomycetales;f__Sanguibacteraceae;g__Sanguibacter;s__**	224	166	3.454 E-01	0.810	0.616	1.030
**k__Bacteria;p__Proteobacteria;c__Alphaproteobacteria;o__Rickettsiales;f__mitochondria;g__;s__**	458	167	3.454 E-01	0.658	0.354	1.007
**k__Bacteria;p__Actinobacteria;c__Actinobacteria;o__Actinomycetales;f__Microbacteriaceae;g__Agrococcus;s__jenensis**	530	168	3.486 E-01	0.335	0.045	0.851
**k__Bacteria;p__Proteobacteria;c__Betaproteobacteria;o__Burkholderiales;f__Oxalobacteraceae;g__;s__**	7	169	3.489 E-01	1.071	0.986	1.165
**k__Bacteria;p__Proteobacteria;c__Gammaproteobacteria;o__Pseudomonadales;f__Moraxellaceae;g__Acinetobacter;s__rhizosphaerae**	543	170	3.517 E-01	0.785	0.564	1.031
**k__Bacteria;p__TM6;c__SJA-4;o__S1198;f__;g__;s__**	510	171	3.517 E-01	1.380	0.946	2.142
**k__Bacteria;p__Proteobacteria;c__Alphaproteobacteria;o__Rhizobiales;f__Rhizobiaceae;g__Rhizobium;s__leguminosarum**	531	172	3.582 E-01	0.606	0.253	0.963
**k__Bacteria;p__Proteobacteria;c__Gammaproteobacteria;o__Enterobacteriales;f__Enterobacteriaceae;g__Serratia;s__marcescens**	204	173	3.694 E-01	0.676	0.366	1.000
**k__Bacteria;p__Bacteroidetes;c__Sphingobacteriia;o__Sphingobacteriales;f__Sphingobacteriaceae;g__;s__**	90	174	3.704 E-01	0.894	0.771	1.023
**k__Bacteria;p__Actinobacteria;c__Actinobacteria;o__Actinomycetales;f__Micrococcaceae;g__Kocuria;s__rhizophila**	322	175	3.752 E-01	1.077	0.981	1.182
**k__Bacteria;p__Acidobacteria;c__Acidobacteria-6;o__iii1-15;f__mb2424;g__;s__**	340	176	3.752 E-01	1.241	0.944	1.638
**k__Bacteria;p__Chloroflexi;c__TK10;o__AKYG885;f__Dolo_23;g__;s__**	392	177	3.752 E-01	1.287	0.933	1.778
**k__Bacteria;p__Proteobacteria;c__Betaproteobacteria;o__Burkholderiales;f__Burkholderiaceae;g__;s__**	464	178	3.855 E-01	0.826	0.633	1.038
**k__Bacteria;p__Proteobacteria;c__Alphaproteobacteria;o__Rhizobiales;f__Methylobacteriaceae;g__Methylobacterium;s__**	261	179	3.855 E-01	0.904	0.792	1.026
**k__Bacteria;p__Cyanobacteria;c__Oscillatoriophycideae;o__Chroococcales;f__;g__;s__**	397	180	3.858 E-01	1.245	0.938	1.664
**k__Bacteria;p__Proteobacteria;c__Gammaproteobacteria;o__Legionellales;f__;g__;s__**	498	181	3.858 E-01	0.895	0.771	1.026
**k__Bacteria;p__Cyanobacteria;c__Chloroplast;o__Chlorophyta;f__Trebouxiophyceae;g__;s__**	61	182	3.858 E-01	0.934	0.849	1.015
**k__Bacteria;p__Proteobacteria;c__Gammaproteobacteria;o__Pasteurellales;f__Pasteurellaceae;g__Haemophilus;s__parainfluenzae**	188	183	3.858 E-01	0.919	0.820	1.021
**k__Bacteria;p__Firmicutes;c__Bacilli;o__Bacillales;f__[Exiguobacteraceae];g__Exiguobacterium;s__**	15	184	3.858 E-01	1.085	0.976	1.205

**k__Bacteria;p__Firmicutes;c__Bacilli;o__Lactobacillales;f__Lactobacillaceae;g__Lactobacillus;s__reuteri**	264	185	3.858 E-01	1.149	0.959	1.381
**k__Bacteria;p__Proteobacteria;c__Alphaproteobacteria;o__Rhizobiales;f__Hyphomicrobiaceae;g__Devosia;s__**	286	186	3.858 E-01	0.904	0.790	1.027
**k__Bacteria;p__Actinobacteria;c__Actinobacteria;o__Actinomycetales;f__Microbacteriaceae;g__Salinibacterium;s__**	108	187	3.858 E-01	0.926	0.834	1.019
**k__Bacteria;p__Proteobacteria;c__Betaproteobacteria;o__Burkholderiales;f__Comamonadaceae;g__Tepidimonas;s__**	159	188	3.858 E-01	0.860	0.699	1.037
**k__Bacteria;p__Proteobacteria;c__Betaproteobacteria;o__Burkholderiales;f__Oxalobacteraceae;g__Cupriavidus;s__**	294	189	3.879 E-01	0.852	0.682	1.040
**k__Bacteria;p__Actinobacteria;c__Coriobacteriia;o__Coriobacteriales;f__Coriobacteriaceae;g__;s__**	96	190	4.046 E-01	1.097	0.969	1.239
**k__Bacteria;p__Proteobacteria;c__Alphaproteobacteria;o__Rhizobiales;f__Beijerinckiaceae;g__;s__**	42	191	4.226 E-01	0.912	0.800	1.028
**k__Bacteria;p__Proteobacteria;c__Betaproteobacteria;o__Neisseriales;f__Neisseriaceae;g__Neisseria;s__**	138	192	4.235 E-01	1.066	0.977	1.163
**k__Bacteria;p__Cyanobacteria;c__4C0d-2;o__MLE1-12;f__;g__;s__**	218	193	4.421 E-01	1.067	0.976	1.166
**k__Bacteria;p__Proteobacteria;c__Deltaproteobacteria;o__Spirobacillales;f__;g__;s__**	483	194	4.421 E-01	0.921	0.816	1.025
**k__Bacteria;p__TM7;c__TM7-3;o__CW040;f__F16;g__;s__**	281	195	4.421 E-01	1.185	0.935	1.511
**k__Bacteria;p__Bacteroidetes;c__Bacteroidia;o__Bacteroidales;f__Prevotellaceae;g__Prevotella;s__**	279	196	4.421 E-01	1.067	0.975	1.168
**k__Bacteria;p__TM7;c__TM7-3;o__CW040;f__;g__;s__**	511	197	4.421 E-01	0.597	0.214	1.038
**k__Bacteria;p__OD1;c__;o__;f__;g__;s__**	438	198	4.421 E-01	0.866	0.701	1.048
**k__Bacteria;p__Proteobacteria;c__Betaproteobacteria;o__Burkholderiales;f__;g__;s__**	463	199	4.435 E-01	1.353	0.893	2.163
**k__Bacteria;p__Firmicutes;c__Clostridia;o__Clostridiales;f__Ruminococcaceae;g__Faecalibacterium;s__prausnitzii**	107	200	4.451 E-01	1.112	0.956	1.291
**k__Bacteria;p__Firmicutes;c__Clostridia;o__Clostridiales;f__Lachnospiraceae;g__Coprococcus;s__**	121	201	4.451 E-01	1.319	0.896	2.000
**k__Bacteria;p__Firmicutes;c__Clostridia;o__Clostridiales;f__Veillonellaceae;g__Dialister;s__**	156	202	4.457 E-01	0.927	0.830	1.029
**k__Bacteria;p__Proteobacteria;c__Betaproteobacteria;o__Rhodocyclales;f__Rhodocyclaceae;g__;s__**	63	203	4.457 E-01	0.869	0.705	1.051
**k__Bacteria;p__Actinobacteria;c__Actinobacteria;o__Actinomycetales;f__Corynebacteriaceae;g__Corynebacterium;s__**	238	204	4.595 E-01	0.940	0.860	1.027
**k__Bacteria;p__Firmicutes;c__Clostridia;o__Clostridiales;f__Clostridiaceae;g__SMB53;s__**	11	205	4.595 E-01	1.072	0.969	1.185
**k__Bacteria;p__Proteobacteria;c__Epsilonproteobacteria;o__Campylobacterales;f__Helicobacteraceae;g__;s__**	487	206	4.595 E-01	1.240	0.907	1.699
**k__Bacteria;p__Proteobacteria;c__Betaproteobacteria;o__Methylophilales;f__Methylophilaceae;g__Methylotenera;s__mobilis**	533	207	4.595 E-01	1.134	0.944	1.360
**k__Bacteria;p__Chloroflexi;c__Anaerolineae;o__SHA-20;f__;g__;s__**	389	208	4.662 E-01	1.211	0.913	1.611
**k__Bacteria;p__Bacteroidetes;c__[Saprospirae];o__[Saprospirales];f__Saprospiraceae;g__;s__**	235	209	4.662 E-01	1.186	0.923	1.524
**k__Bacteria;p__Firmicutes;c__Bacilli;o__Bacillales;f__Bacillaceae;g__Geobacillus;s__**	191	210	4.668 E-01	1.086	0.962	1.225
**k__Bacteria;p__Firmicutes;c__Clostridia;o__Clostridiales;f__Eubacteriaceae;g__Acetobacterium;s__**	421	211	4.676 E-01	1.282	0.887	1.847
**k__Bacteria;p__Proteobacteria;c__Alphaproteobacteria;o__Rhodospirillales;f__Rhodospirillaceae;g__;s__**	64	212	4.714 E-01	1.080	0.964	1.208
**k__Bacteria;p__Actinobacteria;c__Actinobacteria;o__Actinomycetales;f__Nocardioidaceae;g__Friedmanniella;s__**	144	213	4.714 E-01	0.789	0.540	1.095
**k__Bacteria;p__Proteobacteria;c__Alphaproteobacteria;o__Rhizobiales;f__Brucellaceae;g__Ochrobactrum;s__**	450	214	4.714 E-01	0.677	0.342	1.122
**k__Bacteria;p__Actinobacteria;c__Actinobacteria;o__Actinomycetales;f__Geodermatophilaceae;g__;s__**	100	215	4.714 E-01	0.912	0.793	1.042

© 2021 Hsiao CJ et al. *JAMA Network Open.*

**k__Bacteria;p__Chlamydiae;c__Chlamydiia;o__Chlamydiales;f__Parachlamydiaceae;g__;s__**	378	216	4.714 E-01	1.162	0.927	1.454
**k__Bacteria;p__Actinobacteria;c__Actinobacteria;o__Actinomycetales;f__Micrococcaceae;g__Rothia;s__mucilaginosa**	110	217	4.714 E-01	1.055	0.974	1.143
**k__Bacteria;p__Bacteroidetes;c__[Saprospirae];o__[Saprospirales];f__Chitinophagaceae;g__Sediminibacterium;s__**	223	218	4.734 E-01	0.936	0.848	1.032
**k__Bacteria;p__Proteobacteria;c__Betaproteobacteria;o__Neisseriales;f__Neisseriaceae;g__Neisseria;s__cinerea**	523	219	4.791 E-01	1.210	0.907	1.631
**k__Bacteria;p__Acidobacteria;c__Sva0725;o__Sva0725;f__;g__;s__**	343	220	4.849 E-01	1.225	0.898	1.667
**k__Bacteria;p__Bacteroidetes;c__Bacteroidia;o__Bacteroidales;f__Porphyromonadaceae;g__Parabacteroides;s__distasonis**	525	221	4.853 E-01	0.607	0.210	1.097
**k__Bacteria;p__Firmicutes;c__Bacilli;o__Bacillales;f__Bacillaceae;g__Bacillus;s__flexus**	330	222	4.880 E-01	0.800	0.534	1.079
**k__Bacteria;p__Proteobacteria;c__Gammaproteobacteria;o__Legionellales;f__Legionellaceae;g__Legionella;s__**	502	223	5.019 E-01	1.140	0.929	1.397
**k__Bacteria;p__Proteobacteria;c__Gammaproteobacteria;o__Vibrionales;f__Pseudoalteromonadaceae;g__Pseudoalteromonas;s__**	168	224	5.132 E-01	1.108	0.942	1.303
**k__Bacteria;p__Firmicutes;c__Bacilli;o__Bacillales;f__Bacillaceae;g__Anoxybacillus;s__kestanbolensis**	54	225	5.138 E-01	0.912	0.788	1.051
**k__Bacteria;p__Actinobacteria;c__Actinobacteria;o__Actinomycetales;f__Microbacteriaceae;g__Leucobacter;s__**	176	226	5.138 E-01	0.905	0.768	1.051
**k__Bacteria;p__Chloroflexi;c__Ellin6529;o__;f__;g__;s__**	282	227	5.138 E-01	1.185	0.904	1.547
**k__Bacteria;p__Chlamydiae;c__Chlamydiia;o__Chlamydiales;f__Parachlamydiaceae;g__Parachlamydia;s__acanthamoebae**	516	228	5.138 E-01	1.176	0.907	1.523
**k__Bacteria;p__Cyanobacteria;c__4C0d-2;o__YS2;f__;g__;s__**	395	229	5.250 E-01	1.196	0.895	1.597
**k__Bacteria;p__Proteobacteria;c__Gammaproteobacteria;o__Enterobacteriales;f__Enterobacteriaceae;g__Citrobacter;s__**	495	230	5.350 E-01	0.607	0.221	1.211
**k__Bacteria;p__Proteobacteria;c__Alphaproteobacteria;o__Rickettsiales;f__;g__;s__**	29	231	5.376 E-01	0.908	0.769	1.055
**k__Bacteria;p__Actinobacteria;c__Actinobacteria;o__Actinomycetales;f__Dermacoccaceae;g__Dermacoccus;s__**	166	232	5.376 E-01	1.122	0.928	1.355
**k__Bacteria;p__Bacteroidetes;c__Bacteroidia;o__Bacteroidales;f__Porphyromonadaceae;g__Parabacteroides;s__**	249	233	5.388 E-01	0.710	0.344	1.130
**k__Bacteria;p__Firmicutes;c__Clostridia;o__Clostridiales;f__[Tissierellaceae];g__1-68;s__**	207	234	5.391 E-01	1.081	0.950	1.227
**k__Bacteria;p__Proteobacteria;c__Alphaproteobacteria;o__Rhizobiales;f__Phyllobacteriaceae;g__Mesorhizobium;s__**	133	235	5.391 E-01	1.116	0.930	1.333
**k__Bacteria;p__Proteobacteria;c__Betaproteobacteria;o__Burkholderiales;f__Comamonadaceae;g__Variovorax;s__**	465	236	5.391 E-01	1.944	0.673	7.018
**k__Bacteria;p__Bacteroidetes;c__Flavobacteriia;o__Flavobacteriales;f__Flavobacteriaceae;g__Flavobacterium;s__succinicans**	550	237	5.391 E-01	1.360	0.813	2.274
**k__Bacteria;p__Proteobacteria;c__Alphaproteobacteria;o__Rhizobiales;f__Phyllobacteriaceae;g__;s__**	87	238	5.394 E-01	1.108	0.935	1.309
**k__Bacteria;p__Proteobacteria;c__Gammaproteobacteria;o__Pseudomonadales;f__Pseudomonadaceae;g__Pseudomonas;s__**	306	239	5.411 E-01	0.937	0.840	1.044
**k__Bacteria;p__Firmicutes;c__Bacilli;o__Lactobacillales;f__Streptococcaceae;g__Lactococcus;s__**	320	240	5.411 E-01	0.907	0.767	1.059
**k__Bacteria;p__Proteobacteria;c__Alphaproteobacteria;o__Caulobacterales;f__Caulobacteraceae;g__;s__**	78	241	5.440 E-01	0.941	0.851	1.041
**k__Bacteria;p__Proteobacteria;c__Deltaproteobacteria;o__;f__;g__;s__**	474	242	5.440 E-01	1.242	0.864	1.854
**k__Bacteria;p__Bacteroidetes;c__Bacteroidia;o__Bacteroidales;f__Bacteroidaceae;g__Bacteroides;s__ovatus**	335	243	5.440 E-01	1.164	0.899	1.507
**k__Bacteria;p__Actinobacteria;c__Actinobacteria;o__Actinomycetales;f__Pseudonocardiaceae;g__Pseudonocardia;s__**	209	244	5.449 E-01	1.215	0.872	1.700
**k__Bacteria;p__Actinobacteria;c__Thermoleophilia;o__Solirubrobacterales;f__;g__;s__**	6	245	5.449 E-01	1.052	0.965	1.146
**k__Bacteria;p__Proteobacteria;c__Betaproteobacteria;o__Hydrogenophilales;f__Hydrogenophilaceae;g__Thiobacillus;s__**	467	246	5.541 E-01	0.762	0.436	1.144

© 2021 Hsiao CJ et al. *JAMA Network Open.*

k__Bacteria;p__Actinobacteria;c__Actinobacteria;o__Actinomycetales;f__Microbacteriaceae;g__CandidatusAquiluna;s__rubra	65	247	5.645 E-01	0.940	0.840	1.040
k__Bacteria;p__Firmicutes;c__Bacilli;o__Bacillales;f__Listeriaceae;g__Brochothrix;s__	403	248	5.645 E-01	0.742	0.382	1.147
k__Bacteria;p__Proteobacteria;c__Alphaproteobacteria;o__Rhizobiales;f__Bradyrhizobiaceae;g__Balneimonas;s__	449	249	5.658 E-01	0.556	0.126	1.224
k__Bacteria;p__Firmicutes;c__Clostridia;o__Clostridiales;f__Lachnospiraceae;g__;s__	25	250	5.706 E-01	1.061	0.956	1.177
k__Bacteria;p__Actinobacteria;c__Actinobacteria;o__Actinomycetales;f__Nocardiaceae;g__Rhodococcus;s__	102	251	5.706 E-01	1.083	0.940	1.248
k__Bacteria;p__Actinobacteria;c__Actinobacteria;o__Actinomycetales;f__Micrococcaceae;g__Rothia;s__dentocariosa	105	252	5.706 E-01	1.055	0.960	1.158
k__Bacteria;p__Actinobacteria;c__Actinobacteria;o__Actinomycetales;f__;g__;s__	5	253	5.706 E-01	1.044	0.968	1.124
k__Bacteria;p__Proteobacteria;c__Gammaproteobacteria;o__Pasteurellales;f__Pasteurellaceae;g__Haemophilus;s__influenzae	529	254	5.706 E-01	0.531	0.072	1.067
k__Bacteria;p__Proteobacteria;c__Epsilonproteobacteria;o__Campylobacterales;f__Campylobacteraceae;g__Arcobacter;s__	122	255	5.742 E-01	0.866	0.652	1.094
k__Bacteria;p__Proteobacteria;c__Deltaproteobacteria;o__Bdellovibrionales;f__Bacteriovoracaceae;g__;s__	475	256	5.742 E-01	0.683	0.243	1.141
k__Bacteria;p__Proteobacteria;c__Alphaproteobacteria;o__Caulobacterales;f__Caulobacteraceae;g__Brevundimonas;s__diminuta	211	257	5.742 E-01	1.095	0.930	1.287
k__Bacteria;p__Acidobacteria;c__Acidobacteria-6;o__iii1-15;f__;g__;s__	267	258	5.742 E-01	1.145	0.896	1.460
k__Bacteria;p__Firmicutes;c__Bacilli;o__Lactobacillales;f__Carnobacteriaceae;g__;s__	283	259	5.755 E-01	1.103	0.924	1.313
k__Bacteria;p__Fusobacteria;c__Fusobacteriia;o__Fusobacteriales;f__Leptotrichiaceae;g__;s__	140	260	5.815 E-01	0.751	0.394	1.168
k__Bacteria;p__Proteobacteria;c__Alphaproteobacteria;o__Rhizobiales;f__Methylobacteriaceae;g__;s__	109	261	5.858 E-01	1.074	0.943	1.221
k__Bacteria;p__Proteobacteria;c__Betaproteobacteria;o__Burkholderiales;f__Oxalobacteraceae;g__Polynucleobacter;s__cosmopolitanus	524	262	5.873 E-01	0.910	0.759	1.070
k__Bacteria;p__Firmicutes;c__Clostridia;o__Clostridiales;f__Lachnospiraceae;g__Lachnospira;s__	203	263	5.873 E-01	0.753	0.390	1.168
k__Bacteria;p__Proteobacteria;c__Alphaproteobacteria;o__Rhizobiales;f__Hyphomicrobiaceae;g__Rhodoplanes;s__	45	264	5.970 E-01	1.101	0.920	1.312
k__Bacteria;p__Proteobacteria;c__Betaproteobacteria;o__Burkholderiales;f__Burkholderiaceae;g__Lautropia;s__	149	265	6.031 E-01	0.919	0.781	1.069
k__Bacteria;p__Proteobacteria;c__Alphaproteobacteria;o__Rhodospirillales;f__Acetobacteraceae;g__Roseomonas;s__mucosa	99	266	6.122 E-01	1.140	0.886	1.464
k__Bacteria;p__Bacteroidetes;c__Flavobacteriia;o__Flavobacteriales;f__Flavobacteriaceae;g__Flavobacterium;s__	69	267	6.122 E-01	1.058	0.951	1.175
k__Bacteria;p__TM7;c__TM7-3;o__I025;f__;g__;s__	512	268	6.166 E-01	0.735	0.294	1.155
k__Bacteria;p__Firmicutes;c__Clostridia;o__Clostridiales;f__[Tissierellaceae];g__Peptoniphilus;s__	186	269	6.263 E-01	0.962	0.894	1.036
k__Bacteria;p__Firmicutes;c__Bacilli;o__Lactobacillales;f__Streptococcaceae;g__Streptococcus;s__	34	270	6.272 E-01	1.057	0.951	1.179
k__Bacteria;p__Bacteroidetes;c__[Rhodothermi];o__[Rhodothermales];f__Rhodothermaceae;g__Rubricoccus;s__	364	271	6.297 E-01	1.099	0.911	1.320
k__Bacteria;p__Proteobacteria;c__Gammaproteobacteria;o__Alteromonadales;f__[Chromatiaceae];g__;s__	490	272	6.311 E-01	0.442	0.044	1.474
k__Bacteria;p__Firmicutes;c__Bacilli;o__Lactobacillales;f__Aerococcaceae;g__Alloiococcus;s__otitis	536	273	6.325 E-01	0.629	0.164	1.286
k__Bacteria;p__Bacteroidetes;c__Bacteroidia;o__Bacteroidales;f__Bacteroidaceae;g__Bacteroides;s__	328	274	6.325 E-01	0.929	0.801	1.069
k__Bacteria;p__Proteobacteria;c__Gammaproteobacteria;o__Methylococcales;f__Methylococcaceae;g__Methylocaldum;s__	504	275	6.325 E-01	0.922	0.778	1.073
k__Bacteria;p__Proteobacteria;c__Alphaproteobacteria;o__Rhodospirillales;f__Rhodospirillaceae;g__Azospirillum;s__	457	276	6.325 E-01	0.811	0.510	1.179
k__Bacteria;p__Proteobacteria;c__Gammaproteobacteria;o__Aeromonadales;f__Aeromonadaceae;g__;s__	72	277	6.325 E-01	0.924	0.786	1.073

© 2021 Hsiao CJ et al. *JAMA Network Open.*

k__Bacteria;p__Tenericutes;c__Mollicutes;o__Mycoplasmatales;f__Mycoplasmataceae;g__Mycoplasma;s__	254	278	6.366 E-01	1.131	0.880	1.451
k__Bacteria;p__Actinobacteria;c__Actinobacteria;o__Actinomycetales;f__ACK-M1;g__;s__	344	279	6.366 E-01	0.917	0.763	1.080
k__Bacteria;p__Cyanobacteria;c__Synechococcophycideae;o__Synechococcales;f__Synechococcaceae;g__Synechococcus;s__	21	280	6.383 E-01	0.946	0.842	1.051
k__Bacteria;p__Bacteroidetes;c__Cytophagia;o__Cytophagales;f__Cytophagaceae;g__Hymenobacter;s__	39	281	6.385 E-01	1.049	0.953	1.152
k__Bacteria;p__Firmicutes;c__Clostridia;o__Clostridiales;f__[Tissierellaceae];g__WAL_1855D;s__	272	282	6.482 E-01	0.934	0.807	1.065
k__Bacteria;p__Bacteroidetes;c__Flavobacteriia;o__Flavobacteriales;f__Flavobacteriaceae;g__;s__	375	283	6.482 E-01	0.917	0.762	1.084
k__Bacteria;p__Proteobacteria;c__Alphaproteobacteria;o__Rhodobacterales;f__Rhodobacteraceae;g__;s__	13	284	6.539 E-01	0.947	0.847	1.056
k__Bacteria;p__Bacteroidetes;c__Cytophagia;o__Cytophagales;f__Cytophagaceae;g__Rudanella;s__	373	285	6.539 E-01	1.099	0.900	1.335
k__Bacteria;p__TM6;c__F38;o__;f__;g__;s__	97	286	6.618 E-01	0.917	0.759	1.089
k__Bacteria;p__Actinobacteria;c__Actinobacteria;o__Actinomycetales;f__Nocardioidaceae;g__Nocardioides;s__plantarum	127	287	6.623 E-01	0.915	0.754	1.094
k__Bacteria;p__Chlamydiae;c__Chlamydiia;o__Chlamydiales;f__Parachlamydiaceae;g__CandidatusProtochlamydia;s__	379	288	6.623 E-01	0.821	0.454	1.149
k__Bacteria;p__Firmicutes;c__Bacilli;o__Lactobacillales;f__Streptococcaceae;g__Streptococcus;s__minor	143	289	6.646 E-01	0.878	0.654	1.137
k__Bacteria;p__Firmicutes;c__Bacilli;o__Lactobacillales;f__Aerococcaceae;g__Alloiococcus;s__	300	290	6.651 E-01	1.026	0.972	1.083
k__Bacteria;p__TM7;c__;o__;f__;g__;s__	75	291	6.651 E-01	0.924	0.772	1.083
k__Bacteria;p__Firmicutes;c__Bacilli;o__Bacillales;f__Staphylococcaceae;g__Staphylococcus;s__pettenkoferi	538	292	6.651 E-01	0.506	0.064	1.578
k__Bacteria;p__Actinobacteria;c__Actinobacteria;o__Actinomycetales;f__Sporichthyaceae;g__;s__	146	293	6.651 E-01	1.122	0.874	1.429
k__Bacteria;p__Proteobacteria;c__Betaproteobacteria;o__Neisseriales;f__Neisseriaceae;g__Kingella;s__	226	294	6.651 E-01	0.895	0.690	1.116
k__Bacteria;p__Actinobacteria;c__Actinobacteria;o__Actinomycetales;f__Corynebacteriaceae;g__Corynebacterium;s__bovis	521	295	6.651 E-01	1.832	0.669	22.122
k__Bacteria;p__Firmicutes;c__Bacilli;o__Bacillales;f__Staphylococcaceae;g__Salinicoccus;s__	406	296	6.651 E-01	1.799	0.684	21.010
k__Bacteria;p__Actinobacteria;c__Actinobacteria;o__Actinomycetales;f__Nocardioidaceae;g__;s__	46	297	6.651 E-01	0.947	0.841	1.063
k__Bacteria;p__Proteobacteria;c__Betaproteobacteria;o__SC-I-84;f__;g__;s__	473	298	6.651 E-01	1.100	0.892	1.346
k__Bacteria;p__Proteobacteria;c__Gammaproteobacteria;o__Vibrionales;f__Vibrionaceae;g__Vibrio;s__	299	299	6.651 E-01	1.170	0.827	1.642
k__Bacteria;p__[Thermi];c__Deinococci;o__Deinococcales;f__Deinococcaceae;g__Deinococcus;s__aquatilis	112	300	6.651 E-01	0.927	0.779	1.084
k__Bacteria;p__Bacteroidetes;c__Flavobacteriia;o__Flavobacteriales;f__Flavobacteriaceae;g__Capnocytophaga;s__	117	301	6.694 E-01	0.944	0.830	1.066
k__Bacteria;p__Actinobacteria;c__Actinobacteria;o__Actinomycetales;f__Geodermatophilaceae;g__Geodermatophilus;s__	346	302	6.802 E-01	0.590	0.113	1.515
k__Bacteria;p__Gemmatimonadetes;c__Gemm-3;o__;f__;g__;s__	431	303	6.802 E-01	1.465	0.662	5.137
k__Bacteria;p__Actinobacteria;c__Actinobacteria;o__Actinomycetales;f__Micromonosporaceae;g__;s__	252	304	6.816 E-01	0.871	0.627	1.164
k__Bacteria;p__Proteobacteria;c__Gammaproteobacteria;o__Pseudomonadales;f__Moraxellaceae;g__Alkanindiges;s__	290	305	6.816 E-01	1.151	0.834	1.574
k__Bacteria;p__Proteobacteria;c__Alphaproteobacteria;o__Rhizobiales;f__Aurantimonadaceae;g__;s__	162	306	6.816 E-01	1.072	0.916	1.250
k__Bacteria;p__Proteobacteria;c__Alphaproteobacteria;o__Rhodobacterales;f__Rhodobacteraceae;g__Paracoccus;s__marcusii	38	307	6.876 E-01	0.946	0.833	1.069
k__Bacteria;p__Proteobacteria;c__Alphaproteobacteria;o__Sphingomonadales;f__Sphingomonadaceae;g__Sphingobium;s__	256	308	6.977 E-01	0.934	0.795	1.087

© 2021 Hsiao CJ et al. *JAMA Network Open.*

**k__Bacteria;p__Bacteroidetes;c__[Saprospirae];o__[Saprospirales];f__Chitinophagaceae;g__Segetibacter;s__**	365	309	6.977 E-01	0.793	0.385	1.244
**k__Bacteria;p__Proteobacteria;c__Alphaproteobacteria;o__Rhizobiales;f__Phyllobacteriaceae;g__Aminobacter;s__**	273	310	6.977 E-01	0.924	0.766	1.102
**k__Bacteria;p__Actinobacteria;c__Actinobacteria;o__Actinomycetales;f__Micrococcaceae;g__Arthrobacter;s__**	163	311	6.996 E-01	1.069	0.914	1.246
**k__Bacteria;p__Proteobacteria;c__Alphaproteobacteria;o__Rhodobacterales;f__Rhodobacteraceae;g__Rhodobacter;s__**	455	312	6.996 E-01	0.915	0.738	1.115
**k__Bacteria;p__Proteobacteria;c__Gammaproteobacteria;o__Vibrionales;f__Vibrionaceae;g__Photobacterium;s__**	507	313	6.996 E-01	0.805	0.398	1.228
**k__Bacteria;p__Firmicutes;c__Bacilli;o__Lactobacillales;f__Enterococcaceae;g__Enterococcus;s__**	193	314	7.030 E-01	0.847	0.553	1.213
**k__Bacteria;p__Firmicutes;c__Clostridia;o__Clostridiales;f__Veillonellaceae;g__;s__**	424	315	7.152 E-01	0.837	0.503	1.223
**k__Bacteria;p__Proteobacteria;c__Alphaproteobacteria;o__Rhizobiales;f__Methylocystaceae;g__Methylosinus;s__**	41	316	7.188 E-01	0.941	0.809	1.084
**k__Bacteria;p__Actinobacteria;c__KIST-JJY010;o__;f__;g__;s__**	357	317	7.291 E-01	0.926	0.759	1.107
**k__Bacteria;p__Firmicutes;c__Bacilli;o__Bacillales;f__Paenibacillaceae;g__Aneurinibacillus;s__**	404	318	7.370 E-01	0.756	0.299	1.365
**k__Bacteria;p__Firmicutes;c__Bacilli;o__Lactobacillales;f__Enterococcaceae;g__;s__**	130	319	7.473 E-01	0.924	0.749	1.117
**k__Bacteria;p__Actinobacteria;c__Acidimicrobiia;o__Acidimicrobiales;f__Microthrixaceae;g__CandidatusMicrothrix;s__parvicella**	288	320	7.479 E-01	0.845	0.513	1.242
**k__Bacteria;p__Actinobacteria;c__Thermoleophilia;o__Solirubrobacterales;f__Conexibacteraceae;g__;s__**	123	321	7.479 E-01	1.092	0.871	1.357
**k__Bacteria;p__Proteobacteria;c__Alphaproteobacteria;o__Rhodobacterales;f__Rhodobacteraceae;g__Rubellimicrobium;s__**	120	322	7.491 E-01	0.949	0.827	1.079
**k__Bacteria;p__Cyanobacteria;c__Chloroplast;o__Chlorophyta;f__Chlamydomonadaceae;g__;s__**	396	323	7.501 E-01	0.925	0.751	1.117
**k__Bacteria;p__Acidobacteria;c__[Chloracidobacteria];o__RB41;f__Ellin6075;g__;s__**	26	324	7.501 E-01	1.048	0.927	1.181
**k__Bacteria;p__Actinobacteria;c__Actinobacteria;o__Actinomycetales;f__Propionibacteriaceae;g__Luteococcus;s__**	352	325	7.501 E-01	1.293	0.664	3.152
**k__Bacteria;p__Firmicutes;c__Clostridia;o__Clostridiales;f__Clostridiaceae;g__;s__**	12	326	7.501 E-01	0.954	0.841	1.074
**k__Bacteria;p__Proteobacteria;c__Alphaproteobacteria;o__Rhizobiales;f__Methylocystaceae;g__;s__**	453	327	7.501 E-01	0.754	0.275	1.389
**k__Bacteria;p__Actinobacteria;c__Acidimicrobiia;o__Acidimicrobiales;f__EB1017;g__;s__**	44	328	7.501 E-01	0.936	0.787	1.105
**k__Bacteria;p__Cyanobacteria;c__Chloroplast;o__Chlorophyta;f__;g__;s__**	86	329	7.501 E-01	0.958	0.853	1.065
**k__Bacteria;p__Firmicutes;c__Clostridia;o__Clostridiales;f__Peptostreptococcaceae;g__Peptostreptococcus;s__anaerobius**	517	330	7.502 E-01	0.684	0.163	1.517
**k__Bacteria;p__Proteobacteria;c__Gammaproteobacteria;o__Xanthomonadales;f__Xanthomonadaceae;g__Lysobacter;s__brunescens**	522	331	7.520 E-01	0.781	0.280	1.305
**k__Bacteria;p__Actinobacteria;c__Actinobacteria;o__Actinomycetales;f__Corynebacteriaceae;g__Corynebacterium;s__lubricantis**	255	332	7.528 E-01	0.924	0.744	1.128
**k__Bacteria;p__Bacteroidetes;c__Flavobacteriia;o__Flavobacteriales;f__Flavobacteriaceae;g__Flavobacterium;s__frigidarium**	528	333	7.528 E-01	0.617	0.120	1.875
**k__Bacteria;p__Firmicutes;c__Clostridia;o__Clostridiales;f__Ruminococcaceae;g__Ruminococcus;s__**	80	334	7.528 E-01	0.935	0.778	1.110
**k__Bacteria;p__Firmicutes;c__Bacilli;o__Turicibacterales;f__Turicibacteraceae;g__Turicibacter;s__**	95	335	7.559 E-01	1.061	0.905	1.238
**k__Bacteria;p__Proteobacteria;c__Alphaproteobacteria;o__Rhodobacterales;f__Hyphomonadaceae;g__;s__**	106	336	7.638 E-01	1.093	0.855	1.384
**k__Bacteria;p__Bacteroidetes;c__Flavobacteriia;o__Flavobacteriales;f__[Weeksellaceae];g__Cloacibacterium;s__**	332	337	7.638 E-01	1.057	0.909	1.224
**k__Bacteria;p__Proteobacteria;c__Alphaproteobacteria;o__Rhizobiales;f__Hyphomicrobiaceae;g__Hyphomicrobium;s__**	37	338	7.642 E-01	0.954	0.835	1.080
**k__Bacteria;p__Spirochaetes;c__Spirochaetes;o__Spirochaetales;f__Spirochaetaceae;g__Treponema;s__**	200	339	7.752 E-01	1.118	0.810	1.526

© 2021 Hsiao CJ et al. *JAMA Network Open.*

**k__Bacteria;p__Proteobacteria;c__Alphaproteobacteria;o__Rhizobiales;f__Rhizobiaceae;g__Rhizobium;s__**	291	340	7.752 E-01	1.120	0.808	1.532
**k__Bacteria;p__Actinobacteria;c__Actinobacteria;o__Actinomycetales;f__Streptomycetaceae;g__Streptomyces;s__**	354	341	7.752 E-01	1.243	0.656	2.371
**k__Bacteria;p__Proteobacteria;c__Betaproteobacteria;o__Procabacteriales;f__Procabacteriaceae;g__;s__**	470	342	7.926 E-01	0.899	0.630	1.188
**k__Bacteria;p__Firmicutes;c__Bacilli;o__Bacillales;f__Bacillaceae;g__Bacillus;s__selenatarsenatis**	547	343	7.969 E-01	0.786	0.316	1.417
**k__Bacteria;p__Proteobacteria;c__Betaproteobacteria;o__Burkholderiales;f__Oxalobacteraceae;g__Ralstonia;s__**	136	344	7.978 E-01	0.962	0.860	1.075
**k__Bacteria;p__Proteobacteria;c__Deltaproteobacteria;o__Bdellovibrionales;f__Bdellovibrionaceae;g__Bdellovibrio;s__**	124	345	8.194 E-01	1.068	0.873	1.296
**k__Bacteria;p__Chloroflexi;c__Anaerolineae;o__Caldilineales;f__Caldilineaceae;g__Caldilinea;s__**	387	346	8.194 E-01	1.066	0.874	1.289
**k__Bacteria;p__Firmicutes;c__Clostridia;o__Clostridiales;f__Lachnospiraceae;g__Moryella;s__**	73	347	8.194 E-01	0.954	0.822	1.093
**k__Bacteria;p__Firmicutes;c__Clostridia;o__Clostridiales;f__Lachnospiraceae;g__Blautia;s__**	301	348	8.194 E-01	0.956	0.833	1.092
**k__Bacteria;p__Proteobacteria;c__Gammaproteobacteria;o__Alteromonadales;f__[Chromatiaceae];g__Rheinheimera;s__**	326	349	8.263 E-01	0.865	0.527	1.312
**k__Bacteria;p__Bacteroidetes;c__Cytophagia;o__Cytophagales;f__Cytophagaceae;g__Spirosoma;s__**	245	350	8.291 E-01	1.058	0.882	1.258
**k__Bacteria;p__Chloroflexi;c__TK17;o__mle1-48;f__;g__;s__**	393	351	8.427 E-01	1.134	0.742	1.716
**k__Bacteria;p__Firmicutes;c__Bacilli;o__Lactobacillales;f__Enterococcaceae;g__Vagococcus;s__**	411	352	8.457 E-01	0.613	0.075	2.406
**k__Bacteria;p__Proteobacteria;c__Gammaproteobacteria;o__Pseudomonadales;f__Moraxellaceae;g__Acinetobacter;s__johnsonii**	232	353	8.473 E-01	1.048	0.902	1.216
**k__Bacteria;p__Proteobacteria;c__Gammaproteobacteria;o__Legionellales;f__Legionellaceae;g__;s__**	17	354	8.473 E-01	1.039	0.916	1.175
**k__Bacteria;p__Chlamydiae;c__Chlamydiia;o__Chlamydiales;f__;g__;s__**	67	355	8.532 E-01	0.964	0.850	1.083
**k__Bacteria;p__Proteobacteria;c__Betaproteobacteria;o__Rhodocyclales;f__Rhodocyclaceae;g__Hydrogenophilus;s__**	472	356	8.532 E-01	0.872	0.507	1.312
**k__Bacteria;p__[Thermi];c__Deinococci;o__Deinococcales;f__Deinococcaceae;g__CM44;s__**	336	357	8.720 E-01	1.081	0.818	1.408
**k__Bacteria;p__Firmicutes;c__Bacilli;o__Bacillales;f__Bacillaceae;g__Bacillus;s__**	126	358	8.720 E-01	1.040	0.908	1.189
**k__Bacteria;p__Planctomycetes;c__Planctomycetia;o__B97;f__;g__;s__**	445	359	8.720 E-01	1.095	0.794	1.485
**k__Bacteria;p__Actinobacteria;c__Actinobacteria;o__Actinomycetales;f__Actinomycetaceae;g__Mobiluncus;s__**	345	360	8.737 E-01	1.090	0.797	1.468
**k__Bacteria;p__Actinobacteria;c__Actinobacteria;o__Actinomycetales;f__Pseudonocardiaceae;g__Saccharopolyspora;s__**	353	361	8.761 E-01	0.855	0.415	1.386
**k__Bacteria;p__Firmicutes;c__Clostridia;o__Clostridiales;f__Clostridiaceae;g__Clostridium;s__**	182	362	8.761 E-01	1.045	0.893	1.219
**k__Bacteria;p__Actinobacteria;c__Actinobacteria;o__Actinomycetales;f__Nocardiopsaceae;g__;s__**	351	363	8.761 E-01	0.797	0.293	1.628
**k__Bacteria;p__Firmicutes;c__Clostridia;o__Clostridiales;f__Peptostreptococcaceae;g__Peptostreptococcus;s__**	31	364	8.770 E-01	1.037	0.909	1.177
**k__Bacteria;p__Proteobacteria;c__Deltaproteobacteria;o__Myxococcales;f__;g__;s__**	233	365	8.954 E-01	1.044	0.888	1.220
**k__Bacteria;p__Firmicutes;c__Bacilli;o__Lactobacillales;f__Aerococcaceae;g__;s__**	333	366	8.954 E-01	1.049	0.875	1.250
**k__Bacteria;p__Proteobacteria;c__Alphaproteobacteria;o__Sphingomonadales;f__Sphingomonadaceae;g__Sphingopyxis;s__alaskensis**	325	367	8.954 E-01	0.834	0.391	1.574
**k__Bacteria;p__Proteobacteria;c__Alphaproteobacteria;o__Rhodobacterales;f__Rhodobacteraceae;g__Paracoccus;s__aminovorans**	98	368	8.954 E-01	1.059	0.855	1.314
**k__Bacteria;p__Actinobacteria;c__Actinobacteria;o__Actinomycetales;f__Cellulomonadaceae;g__Cellulomonas;s__**	298	369	8.954 E-01	0.949	0.774	1.146
**k__Bacteria;p__Cyanobacteria;c__Chloroplast;o__Stramenopiles;f__;g__;s__**	77	370	8.954 E-01	1.028	0.925	1.137

© 2021 Hsiao CJ et al. *JAMA Network Open.*

k__Bacteria;p__Bacteroidetes;c__Bacteroidia;o__Bacteroidales;f__Bacteroidaceae;g__Bacteroides;s__uniformis	250	371	8.954 E-01	1.049	0.873	1.251
k__Bacteria;p__Proteobacteria;c__Gammaproteobacteria;o__Legionellales;f__Coxiellaceae;g__Aquicella;s__	500	372	9.003 E-01	0.954	0.786	1.134
k__Bacteria;p__Actinobacteria;c__Actinobacteria;o__Actinomycetales;f__Micrococcaceae;g__Arthrobacter;s__psychrolactophilus	540	373	9.003 E-01	1.115	0.714	1.694
k__Bacteria;p__Bacteroidetes;c__Cytophagia;o__Cytophagales;f__Cytophagaceae;g__Dyadobacter;s__	331	374	9.003 E-01	0.949	0.769	1.153
k__Bacteria;p__Actinobacteria;c__Acidimicrobiia;o__Acidimicrobiales;f__C111;g__;s__	47	375	9.053 E-01	0.971	0.864	1.084
k__Bacteria;p__Bacteroidetes;c__[Saprospirae];o__[Saprospirales];f__Chitinophagaceae;g__Flavisolibacter;s__	164	376	9.056 E-01	0.937	0.700	1.189
k__Bacteria;p__Bacteroidetes;c__[Saprospirae];o__[Saprospirales];f__Chitinophagaceae;g__;s__	141	377	9.066 E-01	0.962	0.821	1.117
k__Bacteria;p__Firmicutes;c__Clostridia;o__Clostridiales;f__;g__;s__	270	378	9.072 E-01	0.953	0.781	1.149
k__Bacteria;p__Actinobacteria;c__Actinobacteria;o__Actinomycetales;f__Williamsiaceae;g__Williamsia;s__	355	379	9.072 E-01	1.104	0.721	1.648
k__Bacteria;p__Proteobacteria;c__Alphaproteobacteria;o__Rhodobacterales;f__Rhodobacteraceae;g__Jannaschia;s__	454	380	9.072 E-01	1.105	0.725	1.639
k__Bacteria;p__Proteobacteria;c__Gammaproteobacteria;o__Alteromonadales;f__Psychromonadaceae;g__Psychromonas;s__	317	381	9.072 E-01	0.901	0.554	1.327
k__Bacteria;p__Proteobacteria;c__Betaproteobacteria;o__SBla14;f__;g__;s__	161	382	9.118 E-01	1.103	0.723	1.640
k__Bacteria;p__Proteobacteria;c__Gammaproteobacteria;o__Oceanospirillales;f__Halomonadaceae;g__Halomonas;s__	246	383	9.275 E-01	0.963	0.822	1.130
k__Bacteria;p__Actinobacteria;c__Actinobacteria;o__Actinomycetales;f__Gordoniaceae;g__Gordonia;s__	280	384	9.305 E-01	1.074	0.774	1.462
k__Bacteria;p__Bacteroidetes;c__Sphingobacteriia;o__Sphingobacteriales;f__;g__;s__	377	385	9.315 E-01	0.902	0.497	1.346
k__Bacteria;p__Actinobacteria;c__Actinobacteria;o__Actinomycetales;f__Nakamurellaceae;g__;s__	248	386	9.391 E-01	0.948	0.739	1.190
k__Bacteria;p__Actinobacteria;c__Acidimicrobiia;o__Acidimicrobiales;f__;g__;s__	10	387	9.448 E-01	1.023	0.922	1.132
k__Bacteria;p__Proteobacteria;c__Alphaproteobacteria;o__Rhodospirillales;f__Acetobacteraceae;g__Roseococcus;s__	456	388	9.503 E-01	1.062	0.797	1.389
k__Bacteria;p__Nitrospirae;c__Nitrospira;o__Nitrospirales;f__Nitrospiraceae;g__Nitrospira;s__	437	389	9.618 E-01	1.059	0.791	1.389
k__Bacteria;p__TM7;c__SC3;o__;f__;g__;s__	28	390	9.618 E-01	0.977	0.872	1.088
k__Bacteria;p__Firmicutes;c__Clostridia;o__Clostridiales;f__Veillonellaceae;g__Veillonella;s__parvula	185	391	9.621 E-01	1.046	0.837	1.293
k__Bacteria;p__Proteobacteria;c__Alphaproteobacteria;o__BD7-3;f__;g__;s__	447	392	9.703 E-01	1.084	0.701	1.628
k__Bacteria;p__Bacteroidetes;c__Bacteroidia;o__Bacteroidales;f__Porphyromonadaceae;g__Paludibacter;s__	229	393	9.841 E-01	0.947	0.700	1.233
k__Bacteria;p__Proteobacteria;c__Betaproteobacteria;o__Burkholderiales;f__Oxalobacteraceae;g__Janthinobacterium;s__	91	394	9.905 E-01	0.971	0.827	1.128
k__Bacteria;p__Bacteroidetes;c__Bacteroidia;o__Bacteroidales;f__Bacteroidaceae;g__Bacteroides;s__caccae	329	395	9.905 E-01	0.923	0.566	1.375
k__Bacteria;p__Proteobacteria;c__Gammaproteobacteria;o__Xanthomonadales;f__Xanthomonadaceae;g__Pseudoxanthomonas;s__mexicana	84	396	9.905 E-01	1.059	0.763	1.435
k__Bacteria;p__Actinobacteria;c__Actinobacteria;o__Actinomycetales;f__Mycobacteriaceae;g__Mycobacterium;s__	1	397	9.905 E-01	0.985	0.904	1.068
k__Bacteria;p__Actinobacteria;c__Actinobacteria;o__Actinomycetales;f__Intrasporangiaceae;g__;s__	68	398	9.905 E-01	0.978	0.864	1.100
k__Bacteria;p__Firmicutes;c__Clostridia;o__Thermoanaerobacterales;f__Caldicellulosiruptoraceae;g__Caldicellulosiruptor;s__saccharolyticus	213	399	9.905 E-01	1.042	0.818	1.311
k__Bacteria;p__Firmicutes;c__Clostridia;o__Clostridiales;f__Veillonellaceae;g__Veillonella;s__dispar	48	400	9.905 E-01	1.013	0.939	1.094

© 2021 Hsiao CJ et al. *JAMA Network Open.*

**k__Bacteria;p__Gemmatimonadetes;c__Gemmatimonadetes;o__Gemmatimonadales;f__;g__;s__**	433	401	9.905 E-01	1.047	0.788	1.361
**k__Bacteria;p__Bacteroidetes;c__Flavobacteriia;o__Flavobacteriales;f__[Weeksellaceae];g__Chryseobacterium;s__**	237	402	9.905 E-01	0.981	0.875	1.097
**k__Bacteria;p__Firmicutes;c__Clostridia;o__Clostridiales;f__Veillonellaceae;g__Megasphaera;s__**	212	403	9.905 E-01	1.023	0.889	1.171
**k__Bacteria;p__Firmicutes;c__Clostridia;o__Clostridiales;f__Lachnospiraceae;g__Oribacterium;s__**	79	404	9.905 E-01	1.022	0.893	1.162
**k__Bacteria;p__Firmicutes;c__Erysipelotrichi;o__Erysipelotrichales;f__Erysipelotrichaceae;g__;s__**	115	405	9.905 E-01	0.959	0.734	1.230
**k__Bacteria;p__Bacteroidetes;c__Bacteroidia;o__Bacteroidales;f__Porphyromonadaceae;g__Porphyromonas;s__**	155	406	9.905 E-01	0.985	0.897	1.081
**k__Bacteria;p__Bacteroidetes;c__Sphingobacteriia;o__Sphingobacteriales;f__Sphingobacteriaceae;g__Sphingobacterium;s__multivorum**	535	407	9.905 E-01	1.054	0.745	1.453
**k__Bacteria;p__Proteobacteria;c__Gammaproteobacteria;o__Legionellales;f__Coxiellaceae;g__;s__**	499	408	9.905 E-01	1.034	0.827	1.276
**k__Bacteria;p__Actinobacteria;c__Acidimicrobiia;o__Acidimicrobiales;f__Microthrixaceae;g__;s__**	53	409	9.905 E-01	0.976	0.829	1.138
**k__Bacteria;p__Proteobacteria;c__Gammaproteobacteria;o__Xanthomonadales;f__Xanthomonadaceae;g__Luteimonas;s__**	508	410	9.905 E-01	0.861	0.197	2.106
**k__Bacteria;p__Firmicutes;c__Bacilli;o__Lactobacillales;f__Carnobacteriaceae;g__Granulicatella;s__**	27	411	9.905 E-01	0.988	0.909	1.071
**k__Bacteria;p__Firmicutes;c__Bacilli;o__Bacillales;f__Planococcaceae;g__Planomicrobium;s__**	178	412	9.905 E-01	1.017	0.909	1.133
**k__Bacteria;p__Proteobacteria;c__Betaproteobacteria;o__Burkholderiales;f__Alcaligenaceae;g__;s__**	43	413	9.905 E-01	0.986	0.893	1.083
**k__Bacteria;p__Firmicutes;c__Bacilli;o__Gemellales;f__Gemellaceae;g__Gemella;s__**	116	414	9.905 E-01	1.030	0.844	1.256
**k__Bacteria;p__Proteobacteria;c__Gammaproteobacteria;o__Enterobacteriales;f__Enterobacteriaceae;g__Proteus;s__**	222	415	9.905 E-01	1.020	0.886	1.165
**k__Bacteria;p__Proteobacteria;c__Gammaproteobacteria;o__Alteromonadales;f__Shewanellaceae;g__Shewanella;s__algae**	251	416	9.905 E-01	0.978	0.840	1.141
**k__Bacteria;p__Proteobacteria;c__Alphaproteobacteria;o__Sphingomonadales;f__;g__;s__**	32	417	9.905 E-01	0.979	0.844	1.128
**k__Bacteria;p__Firmicutes;c__Bacilli;o__Lactobacillales;f__Streptococcaceae;g__;s__**	416	418	9.905 E-01	1.059	0.675	1.592
**k__Bacteria;p__TM7;c__TM7-3;o__;f__;g__;s__**	14	419	9.905 E-01	1.013	0.924	1.109
**k__Bacteria;p__Proteobacteria;c__Betaproteobacteria;o__Burkholderiales;f__Comamonadaceae;g__Hylemonella;s__**	234	420	9.905 E-01	1.097	0.536	2.124
**k__Bacteria;p__Bacteroidetes;c__Sphingobacteriia;o__Sphingobacteriales;f__Sphingobacteriaceae;g__Pedobacter;s__**	89	421	9.905 E-01	1.013	0.922	1.111
**k__Bacteria;p__Cyanobacteria;c__Nostocophycideae;o__Stigonematales;f__Rivulariaceae;g__Calothrix;s__**	284	422	9.905 E-01	0.967	0.743	1.221
**k__Bacteria;p__Proteobacteria;c__Betaproteobacteria;o__Rhodocyclales;f__Rhodocyclaceae;g__Dechloromonas;s__**	187	423	9.905 E-01	1.026	0.840	1.241
**k__Bacteria;p__Proteobacteria;c__Alphaproteobacteria;o__Sphingomonadales;f__Sphingomonadaceae;g__Sphingomonas;s__yabuuchiae**	553	424	9.905 E-01	0.941	0.529	1.467
**k__Bacteria;p__Actinobacteria;c__Actinobacteria;o__Actinomycetales;f__Actinomycetaceae;g__Actinomyces;s__**	30	425	9.905 E-01	0.989	0.909	1.076
**k__Bacteria;p__Proteobacteria;c__Betaproteobacteria;o__Burkholderiales;f__Comamonadaceae;g__;s__**	8	426	9.905 E-01	0.988	0.898	1.087
**k__Bacteria;p__TM6;c__SJA-4;o__;f__;g__;s__**	74	427	9.905 E-01	1.019	0.873	1.179
**k__Bacteria;p__Firmicutes;c__Bacilli;o__Gemellales;f__Gemellaceae;g__;s__**	88	428	9.905 E-01	0.988	0.897	1.086
**k__Bacteria;p__Proteobacteria;c__Betaproteobacteria;o__Neisseriales;f__Neisseriaceae;g__;s__**	266	429	9.905 E-01	1.009	0.939	1.083
**k__Bacteria;p__Actinobacteria;c__Actinobacteria;o__Bifidobacteriales;f__Bifidobacteriaceae;g__Bifidobacterium;s__adolescentis**	152	430	9.905 E-01	0.978	0.807	1.172

**k__Bacteria;p__Actinobacteria;c__Actinobacteria;o__Actinomycetales;f__Micrococcaceae;g__;s__**	118	431	9.905 E-01	1.011	0.922	1.108
**k__Bacteria;p__Proteobacteria;c__Betaproteobacteria;o__Burkholderiales;f__Comamonadaceae;g__Polaromonas;s__**	206	432	9.905 E-01	0.974	0.772	1.218
**k__Bacteria;p__Proteobacteria;c__Betaproteobacteria;o__Methylophilales;f__Methylophilaceae;g__;s__**	24	433	9.905 E-01	1.016	0.885	1.159
**k__Bacteria;p__Proteobacteria;c__Gammaproteobacteria;o__Xanthomonadales;f__Sinobacteraceae;g__;s__**	268	434	9.905 E-01	0.987	0.882	1.104
**k__Bacteria;p__Acidobacteria;c__[Chloracidobacteria];o__RB41;f__;g__;s__**	338	435	9.905 E-01	1.036	0.747	1.402
**k__Bacteria;p__Fusobacteria;c__Fusobacteriia;o__Fusobacteriales;f__Leptotrichiaceae;g__Sneathia;s__**	201	436	9.905 E-01	0.971	0.733	1.248
**k__Bacteria;p__Actinobacteria;c__Actinobacteria;o__Actinomycetales;f__Corynebacteriaceae;g__Corynebacterium;s__variabile**	551	437	9.905 E-01	0.965	0.680	1.317
**k__Bacteria;p__Proteobacteria;c__Betaproteobacteria;o__Rhodocyclales;f__Rhodocyclaceae;g__C39;s__**	471	438	9.905 E-01	1.045	0.672	1.565
**k__Bacteria;p__Actinobacteria;c__Actinobacteria;o__Actinomycetales;f__Actinomycetaceae;g__Varibaculum;s__**	278	439	9.905 E-01	0.974	0.740	1.248
**k__Bacteria;p__Firmicutes;c__Bacilli;o__Bacillales;f__Planococcaceae;g__Paenisporosarcina;s__**	60	440	9.905 E-01	0.991	0.901	1.084
**k__Bacteria;p__TM7;c__TM7-1;o__;f__;g__;s__**	4	441	9.905 E-01	0.990	0.899	1.087
**k__Bacteria;p__Fusobacteria;c__Fusobacteriia;o__Fusobacteriales;f__Fusobacteriaceae;g__Fusobacterium;s__**	66	442	9.905 E-01	1.009	0.923	1.102
**k__Bacteria;p__Proteobacteria;c__Alphaproteobacteria;o__Rhizobiales;f__Bradyrhizobiaceae;g__;s__**	49	443	9.905 E-01	1.011	0.903	1.134
**k__Bacteria;p__Proteobacteria;c__Deltaproteobacteria;o__Myxococcales;f__0319-6G20;g__;s__**	104	444	9.905 E-01	0.969	0.668	1.326
**k__Bacteria;p__Actinobacteria;c__Coriobacteriia;o__Coriobacteriales;f__Coriobacteriaceae;g__Eggerthella;s__lenta**	258	445	9.905 E-01	1.038	0.660	1.573
**k__Bacteria;p__[Thermi];c__Deinococci;o__Deinococcales;f__Trueperaceae;g__Truepera;s__**	92	446	9.905 E-01	1.015	0.842	1.210
**k__Bacteria;p__Firmicutes;c__Bacilli;o__Lactobacillales;f__Aerococcaceae;g__Abiotrophia;s__**	195	447	9.905 E-01	0.989	0.864	1.124
**k__Bacteria;p__Cyanobacteria;c__Oscillatoriophycideae;o__Chroococcales;f__Xenococcaceae;g__Chroococcidiopsis;s__**	304	448	9.905 E-01	1.023	0.765	1.340
**k__Bacteria;p__Firmicutes;c__Clostridia;o__Clostridiales;f__[Tissierellaceae];g__ph2;s__**	419	449	9.905 E-01	0.971	0.650	1.372
**k__Bacteria;p__Proteobacteria;c__Gammaproteobacteria;o__Pseudomonadales;f__Moraxellaceae;g__Enhydrobacter;s__**	103	450	9.905 E-01	1.010	0.896	1.137
**k__Bacteria;p__Proteobacteria;c__Alphaproteobacteria;o__Rhizobiales;f__Brucellaceae;g__;s__**	219	451	9.905 E-01	1.015	0.837	1.222
**k__Bacteria;p__Actinobacteria;c__Actinobacteria;o__Actinomycetales;f__Intrasporangiaceae;g__Arsenicicoccus;s__**	347	452	9.905 E-01	0.938	0.265	2.121
**k__Bacteria;p__Actinobacteria;c__Thermoleophilia;o__Gaiellales;f__;g__;s__**	359	453	9.905 E-01	1.009	0.876	1.155
**k__Bacteria;p__Proteobacteria;c__Alphaproteobacteria;o__Rhizobiales;f__Hyphomicrobiaceae;g__Pedomicrobium;s__**	452	454	9.905 E-01	1.025	0.693	1.469
**k__Bacteria;p__Proteobacteria;c__TA18;o__PHOS-HD29;f__;g__;s__**	70	455	9.905 E-01	0.988	0.822	1.178
**k__Bacteria;p__Proteobacteria;c__Alphaproteobacteria;o__Rhodospirillales;f__Acetobacteraceae;g__;s__**	59	456	9.905 E-01	1.008	0.883	1.147
**k__Bacteria;p__Actinobacteria;c__Actinobacteria;o__Bifidobacteriales;f__Bifidobacteriaceae;g__;s__**	312	457	9.905 E-01	1.010	0.860	1.177
**k__Bacteria;p__Proteobacteria;c__Betaproteobacteria;o__Neisseriales;f__Neisseriaceae;g__Eikenella;s__**	225	458	9.905 E-01	0.982	0.715	1.308
**k__Bacteria;p__Bacteroidetes;c__Bacteroidia;o__Bacteroidales;f__Porphyromonadaceae;g__Tannerella;s__**	369	459	9.905 E-01	0.956	0.338	1.986
**k__Bacteria;p__Proteobacteria;c__Deltaproteobacteria;o__NKB15;f__;g__;s__**	482	460	9.905 E-01	0.984	0.737	1.287
**k__Bacteria;p__Actinobacteria;c__Actinobacteria;o__Actinomycetales;f__Micrococcaceae;g__Micrococcus;s__**	50	461	9.905 E-01	1.012	0.827	1.239

© 2021 Hsiao CJ et al. *JAMA Network Open.*

**k__Bacteria;p__Firmicutes;c__Clostridia;o__Clostridiales;f__Lachnospiraceae;g__Shuttleworthia;s__**	423	462	9.905 E-01	0.978	0.626	1.434
**k__Bacteria;p__Acidobacteria;c__DA052;o__Ellin6513;f__;g__;s__**	341	463	9.905 E-01	1.028	0.531	1.747
**k__Bacteria;p__Proteobacteria;c__Alphaproteobacteria;o__Rhizobiales;f__Methylobacteriaceae;g__Methylobacterium;s__adhaesivum**	227	464	9.905 E-01	0.993	0.860	1.139
**k__Bacteria;p__WS6;c__SC72;o__WCHB1-15;f__;g__;s__**	515	465	9.905 E-01	0.986	0.731	1.303
**k__Bacteria;p__Actinobacteria;c__Actinobacteria;o__Actinomycetales;f__Nocardioidaceae;g__Aeromicrobium;s__**	94	466	9.905 E-01	1.008	0.855	1.177
**k__Bacteria;p__Firmicutes;c__Bacilli;o__Lactobacillales;f__Lactobacillaceae;g__;s__**	412	467	9.905 E-01	1.021	0.582	1.627
**k__Bacteria;p__Proteobacteria;c__Alphaproteobacteria;o__Caulobacterales;f__Caulobacteraceae;g__Mycoplana;s__**	230	468	9.905 E-01	1.007	0.853	1.181
**k__Bacteria;p__Fusobacteria;c__Fusobacteriia;o__Fusobacteriales;f__Leptotrichiaceae;g__Leptotrichia;s__**	85	469	9.905 E-01	0.997	0.911	1.089
**k__Bacteria;p__Proteobacteria;c__Alphaproteobacteria;o__Rhizobiales;f__Bradyrhizobiaceae;g__Bradyrhizobium;s__**	173	470	9.905 E-01	0.994	0.806	1.226
**k__Bacteria;p__Firmicutes;c__Clostridia;o__Clostridiales;f__Veillonellaceae;g__Selenomonas;s__**	172	471	9.905 E-01	0.997	0.891	1.111
**k__Bacteria;p__Bacteroidetes;c__Flavobacteriia;o__Flavobacteriales;f__[Weeksellaceae];g__Wautersiella;s__**	292	472	9.905 E-01	0.993	0.616	1.512
**k__Bacteria;p__Firmicutes;c__Bacilli;o__Lactobacillales;f__Streptococcaceae;g__Streptococcus;s__sobrinus**	548	473	9.905 E-01	0.000	NA	220880.319
**k__Bacteria;p__Chloroflexi;c__Anaerolineae;o__H39;f__;g__;s__**	388	474	9.905 E-01	2719.537	0.000	NA
**k__Bacteria;p__Firmicutes;c__Clostridia;o__Clostridiales;f__Christensenellaceae;g__;s__**	420	475	9.905 E-01	18.386	0.000	NA
**k__Bacteria;p__Synergistetes;c__Synergistia;o__Synergistales;f__Dethiosulfovibrionaceae;g__Pyramidobacter;s__piscolens**	539	476	9.905 E-01	18.766	0.003	NA
**k__Bacteria;p__Firmicutes;c__Bacilli;o__Lactobacillales;f__Lactobacillaceae;g__Pediococcus;s__**	413	477	9.905 E-01	9.553	0.000	NA
**k__Bacteria;p__GN02;c__GKS2-174;o__;f__;g__;s__**	435	478	9.905 E-01	69.417	0.000	NA
**k__Bacteria;p__Firmicutes;c__Bacilli;o__Bacillales;f__Staphylococcaceae;g__Jeotgalicoccus;s__psychrophilus**	541	479	9.905 E-01	0.000	NA	NA
**k__Bacteria;p__Actinobacteria;c__Actinobacteria;o__Actinomycetales;f__Dietziaceae;g__Dietzia;s__**	318	480	9.905 E-01	0.000	NA	120603.867
**k__Bacteria;p__Spirochaetes;c__Spirochaetes;o__Sphaerochaetales;f__Sphaerochaetaceae;g__Sphaerochaeta;s__**	509	481	9.905 E-01	27.369	0.005	NA
**k__Bacteria;p__Proteobacteria;c__Gammaproteobacteria;o__Aeromonadales;f__Aeromonadaceae;g__Tolumonas;s__**	489	482	9.905 E-01	0.021	NA	5711.099
**k__Bacteria;p__GN02;c__BD1-5;o__;f__;g__;s__**	434	483	9.905 E-01	0.001	NA	NA
**k__Bacteria;p__Gemmatimonadetes;c__Gemmatimonadetes;o__;f__;g__;s__**	432	484	9.905 E-01	0.033	NA	NA
**k__Bacteria;p__Bacteroidetes;c__Bacteroidia;o__Bacteroidales;f__S24-7;g__;s__**	370	485	9.905 E-01	0.043	NA	NA
**k__Bacteria;p__Proteobacteria;c__Alphaproteobacteria;o__Rhizobiales;f__Hyphomicrobiaceae;g__Parvibaculum;s__**	451	486	9.905 E-01	0.088	NA	27507934.820
**k__Bacteria;p__Synergistetes;c__Synergistia;o__Synergistales;f__Dethiosulfovibrionaceae;g__Jonquetella;s__anthropi**	518	487	9.905 E-01	0.084	NA	36656530.930
**k__Bacteria;p__Chloroflexi;c__Anaerolineae;o__Anaerolineales;f__Anaerolinaceae;g__Anaerolinea;s__**	385	488	9.905 E-01	0.046	NA	NA
**k__Bacteria;p__Proteobacteria;c__Gammaproteobacteria;o__Oceanospirillales;f__Halomonadaceae;g__;s__**	505	489	9.905 E-01	0.075	NA	NA
**k__Bacteria;p__Firmicutes;c__Bacilli;o__Lactobacillales;f__Leuconostocaceae;g__Weissella;s__**	415	490	9.905 E-01	0.000	NA	NA
**k__Bacteria;p__Proteobacteria;c__Deltaproteobacteria;o__Syntrophobacterales;f__Syntrophaceae;g__Desulfomonile;s__**	484	491	9.905 E-01	0.001	0.000	2851.329

© 2021 Hsiao CJ et al. *JAMA Network Open.*

**k__Bacteria;p__Firmicutes;c__Clostridia;o__Clostridiales;f__Veillonellaceae;g__Schwartzia;s__**	426	492	9.905 E-01	78.634	0.000	NA
**k__Bacteria;p__Proteobacteria;c__Alphaproteobacteria;o__Sphingomonadales;f__Sphingomonadaceae;g__Sphingomonas;s__wittichii**	552	493	9.905 E-01	0.000	NA	NA
**k__Bacteria;p__Aquificae;c__Aquificae;o__Aquificales;f__Aquificaceae;g__;s__**	362	494	9.905 E-01	0.000	NA	NA
**k__Bacteria;p__Actinobacteria;c__Actinobacteria;o__Actinomycetales;f__Pseudonocardiaceae;g__Actinomycetospora;s__**	210	495	9.905 E-01	0.000	0.000	199982.076
**k__Bacteria;p__Chloroflexi;c__Chloroflexi;o__[Roseiflexales];f__[Kouleothrixaceae];g__Kouleothrix;s__**	390	496	9.905 E-01	35.324	0.000	NA
**k__Bacteria;p__WS3;c__PRR-12;o__Sediment-1;f__PRR-10;g__;s__**	514	497	9.905 E-01	77.372	0.000	NA
**k__Bacteria;p__Proteobacteria;c__Gammaproteobacteria;o__Alteromonadales;f__OM60;g__;s__**	491	498	9.905 E-01	86.221	0.000	NA
**k__Bacteria;p__Proteobacteria;c__Deltaproteobacteria;o__NB1-j;f__JTB38;g__;s__**	481	499	9.905 E-01	47.267	0.000	NA
**k__Bacteria;p__Proteobacteria;c__Deltaproteobacteria;o__FAC87;f__;g__;s__**	477	500	9.905 E-01	46.124	0.000	NA
**k__Bacteria;p__Gemmatimonadetes;c__Gemm-1;o__;f__;g__;s__**	430	501	9.905 E-01	41.335	0.000	NA
**k__Bacteria;p__Planctomycetes;c__C6;o__d113;f__;g__;s__**	442	502	9.905 E-01	51.185	0.000	NA
**k__Bacteria;p__Chloroflexi;c__Chloroflexi;o__AKIW781;f__;g__;s__**	391	503	9.905 E-01	0.038	NA	58531943.310
**k__Bacteria;p__Chlamydiae;c__Chlamydiia;o__Chlamydiales;f__Parachlamydiaceae;g__Parachlamydia;s__**	380	504	9.905 E-01	0.007	NA	NA
**k__Bacteria;p__Proteobacteria;c__Deltaproteobacteria;o__Syntrophobacterales;f__Syntrophorhabdaceae;g__;s__**	485	505	9.905 E-01	0.007	NA	NA
**k__Bacteria;p__Cyanobacteria;c__Oscillatoriophycideae;o__Chroococcales;f__Cyanobacteriaceae;g__Cyanobacterium;s__**	398	506	9.905 E-01	0.008	NA	96840309.240
**k__Bacteria;p__Proteobacteria;c__Gammaproteobacteria;o__Legionellales;f__Legionellaceae;g__Tatlockia;s__**	503	507	9.905 E-01	0.011	NA	21006759.360
**k__Bacteria;p__Chlamydiae;c__Chlamydiia;o__Chlamydiales;f__Rhabdochlamydiaceae;g__CandidatusRhabdochlamydia;s__**	381	508	9.905 E-01	0.034	NA	329869.646
**k__Bacteria;p__Fusobacteria;c__Fusobacteriia;o__Fusobacteriales;f__;g__;s__**	428	509	9.905 E-01	0.004	NA	NA
**k__Bacteria;p__Actinobacteria;c__Actinobacteria;o__Actinomycetales;f__Kineosporiaceae;g__Kineococcus;s__**	348	510	9.905 E-01	0.000	NA	NA
**k__Bacteria;p__Armatimonadetes;c__[Fimbriimonadia];o__[Fimbriimonadales];f__[Fimbriimonadaceae];g__Fimbriimonas;s__**	363	511	9.905 E-01	0.000	NA	NA
**k__Bacteria;p__OD1;c__SM2F11;o__;f__;g__;s__**	440	512	9.905 E-01	0.070	NA	662524.372
**k__Bacteria;p__OD1;c__ABY1;o__;f__;g__;s__**	439	513	9.905 E-01	0.027	NA	80027541.210
**k__Bacteria;p__Bacteroidetes;c__Bacteroidia;o__Bacteroidales;f__BA008;g__;s__**	366	514	9.905 E-01	0.036	NA	20653084.220
**k__Bacteria;p__Chlorobi;c__Ignavibacteria;o__Ignavibacteriales;f__Ignavibacteriaceae;g__;s__**	382	515	9.905 E-01	0.042	NA	9289530.494
**k__Bacteria;p__Cyanobacteria;c__4C0d-2;o__SM1D11;f__;g__;s__**	394	516	9.905 E-01	0.042	NA	9289530.494
**k__Bacteria;p__Proteobacteria;c__Gammaproteobacteria;o__Chromatiales;f__Chromatiaceae;g__Allochromatium;s__**	492	517	9.905 E-01	0.057	NA	1985986.742
**k__Bacteria;p__Firmicutes;c__Clostridia;o__Clostridiales;f__Veillonellaceae;g__vadinHB04;s__**	427	518	9.905 E-01	0.023	NA	NA
**k__Bacteria;p__Proteobacteria;c__Deltaproteobacteria;o__MIZ46;f__;g__;s__**	478	519	9.905 E-01	0.020	NA	NA

© 2021 Hsiao CJ et al. *JAMA Network Open.*

**k__Bacteria;p__Proteobacteria;c__Gammaproteobacteria;o__Legionellales;f__Francisellaceae;g__;s__**	501	520	9.905 E-01	0.015	NA	NA
**k__Bacteria;p__Proteobacteria;c__Deltaproteobacteria;o__Bdellovibrionales;f__Bdellovibrionaceae;g__Bdellovibrio;s__bacteriovorus**	520	521	9.905 E-01	0.044	NA	6746394.794
**k__Bacteria;p__Proteobacteria;c__Betaproteobacteria;o__;f__;g__;s__**	461	522	9.905 E-01	0.027	NA	89335300.220
**k__Bacteria;p__Proteobacteria;c__Gammaproteobacteria;o__Chromatiales;f__Halothiobacillaceae;g__Thiovirga;s__**	493	523	9.905 E-01	0.058	NA	1684114.103
**k__Bacteria;p__Proteobacteria;c__Deltaproteobacteria;o__Myxococcales;f__Polyangiaceae;g__;s__**	480	524	9.905 E-01	NA	0.000	NA
**k__Bacteria;p__Firmicutes;c__Bacilli;o__Lactobacillales;f__Aerococcaceae;g__Marinilactibacillus;s__psychrotolerans**	542	525	9.905 E-01	0.004	NA	NA
**k__Bacteria;p__Proteobacteria;c__Alphaproteobacteria;o__Caulobacterales;f__Caulobacteraceae;g__Arthrospira;s__**	448	526	9.905 E-01	0.000	NA	NA
**k__Bacteria;p__Acidobacteria;c__Holophagae;o__Holophagales;f__Holophagaceae;g__Geothrix;s__**	342	527	9.905 E-01	0.003	NA	48283753.330
**k__Bacteria;p__Bacteroidetes;c__Bacteroidia;o__Bacteroidales;f__Porphyromonadaceae;g__;s__**	367	528	9.905 E-01	0.000	NA	NA
**k__Bacteria;p__Firmicutes;c__;o__;f__;g__;s__**	401	529	9.905 E-01	0.000	NA	NA
**k__Bacteria;p__Proteobacteria;c__Betaproteobacteria;o__Neisseriales;f__Neisseriaceae;g__Microvirgula;s__**	468	530	9.905 E-01	0.000	NA	NA
**k__Bacteria;p__Proteobacteria;c__Gammaproteobacteria;o__;f__;g__;s__**	488	531	9.905 E-01	0.000	NA	NA
**k__Bacteria;p__OP11;c__WCHB1-64;o__d153;f__;g__;s__**	441	532	9.905 E-01	0.009	NA	NA
**k__Bacteria;p__Firmicutes;c__Clostridia;o__Clostridiales;f__[Tissierellaceae];g__;s__**	417	533	9.905 E-01	0.015	NA	NA
**k__Bacteria;p__Proteobacteria;c__Gammaproteobacteria;o__Pseudomonadales;f__Moraxellaceae;g__Acinetobacter;s__schindleri**	545	534	9.905 E-01	0.000	NA	NA
**k__Bacteria;p__Proteobacteria;c__Gammaproteobacteria;o__Enterobacteriales;f__Enterobacteriaceae;g__Yersinia;s__**	496	535	9.905 E-01	0.000	NA	NA
**k__Bacteria;p__Proteobacteria;c__Gammaproteobacteria;o__HOC36;f__;g__;s__**	497	536	9.905 E-01	0.009	NA	18524743.660
**k__Bacteria;p__Cyanobacteria;c__Oscillatoriophycideae;o__Oscillatoriales;f__Phormidiaceae;g__Phormidium;s__**	399	537	9.905 E-01	0.001	NA	NA
**k__Bacteria;p__Firmicutes;c__Bacilli;o__Lactobacillales;f__Carnobacteriaceae;g__Trichococcus;s__**	410	538	9.905 E-01	14.193	0.000	NA
**k__Bacteria;p__Proteobacteria;c__Epsilonproteobacteria;o__Campylobacterales;f__Campylobacteraceae;g__Sulfurospirillum;s__**	486	539	9.905 E-01	0.000	NA	NA
**k__Bacteria;p__Firmicutes;c__Clostridia;o__Clostridiales;f__Veillonellaceae;g__Anaeromusa;s__**	425	540	9.905 E-01	0.000	NA	NA
**k__Bacteria;p__Planctomycetes;c__Phycisphaerae;o__Phycisphaerales;f__;g__;s__**	443	541	9.905 E-01	0.102	NA	NA
**k__Bacteria;p__Proteobacteria;c__Deltaproteobacteria;o__Myxococcales;f__OM27;g__;s__**	479	542	9.905 E-01	0.000	NA	NA
**k__Bacteria;p__Planctomycetes;c__Phycisphaerae;o__WD2101;f__;g__;s__**	444	543	9.905 E-01	0.000	NA	NA
**k__Bacteria;p__Firmicutes;c__Bacilli;o__Lactobacillales;f__Aerococcaceae;g__Alkalibacterium;s__**	409	544	9.905 E-01	0.000	NA	NA
**k__Bacteria;p__Bacteroidetes;c__Flavobacteriia;o__Flavobacteriales;f__Flavobacteriaceae;g__Myroides;s__**	376	545	9.905 E-01	0.000	NA	NA
**k__Bacteria;p__Bacteroidetes;c__Cytophagia;o__Cytophagales;f__Cytophagaceae;g__Rhodocytophaga;s__**	372	546	9.905 E-01	0.092	NA	NA
**k__Bacteria;p__Cyanobacteria;c__Synechococcophycideae;o__Pseudanabaenales;f__;g__;s__**	400	547	9.905 E-01	0.057	NA	NA
**k__Bacteria;p__Firmicutes;c__Bacilli;o__Lactobacillales;f__Lactobacillaceae;g__Lactobacillus;s__iners**	150	548	9.905 E-01	0.999	0.861	1.154

© 2021 Hsiao CJ et al. *JAMA Network Open.*

**k__Bacteria;p__Verrucomicrobia;c__[Spartobacteria];o__[Chthoniobacterales];f__[Chthoniobacteraceae];g__DA101;s__**	513	549	9.905 E-01	0.071	NA	NA
**k__Bacteria;p__Proteobacteria;c__Gammaproteobacteria;o__Enterobacteriales;f__Enterobacteriaceae;g__Buchnera;s__**	494	550	9.905 E-01	0.040	NA	NA
**k__Bacteria;p__Bacteroidetes;c__Flavobacteriia;o__Flavobacteriales;f__Cryomorphaceae;g__Fluviicola;s__**	374	551	9.905 E-01	0.052	NA	NA
**k__Bacteria;p__Proteobacteria;c__Alphaproteobacteria;o__Rhodobacterales;f__Rhodobacteraceae;g__Amaricoccus;s__**	289	552	9.916 E-01	1.003	0.633	1.503
**k__Bacteria;p__Proteobacteria;c__Betaproteobacteria;o__Burkholderiales;f__Comamonadaceae;g__Acidovorax;s__delafieldii**	167	553	9.984 E-01	1.000	0.784	1.249

© 2021 Hsiao CJ et al. *JAMA Network Open.*

eTable 3. Association of Individual Taxa and Cluster Class With Composite Infectious Outcome

Variables	Unadjusted Odds Ratio	P value	Adjusted Odds Ratio	P value
Moraxella spp.	1.17 (1.05,1.29)	0.003	1.16 (1.00,1.34)	0.04
Propionibacterium granulosum	0.86 (0.79,0.94)	0.001	0.91 (0.79,1.06)	0.25
Novosphingobium spp.	1.16 (1.10,1.24)	<0.001	1.13 (1.05,1.23)	0.001
Anaerococcus spp.	0.71 (0.59,0.86)	<0.001	0.43 (0.31,0.57)	<0.001
Propionibacterium spp.	1.19 (1.06,1.33)	0.002	0.98 (0.83,1.15)	0.87
Atopobium spp.	0.70 (0.58,0.85)	<0.001	0.69 (0.53,0.89)	0.005
Parvimonas spp.	0.86 (0.79,0.94)	<0.001	0.94 (0.82,1.07)	0.36
Cluster 2 probability	6.18 (3.30,11.7)	<0.001	6.71 (2.91,15.84)	<0.001

© 2021 Hsiao CJ et al. *JAMA Network Open.*

eFigure 1. Taxonomic Classification of 16S rRNA Gene Sequences Identified From Preoperative Nasal Swabs

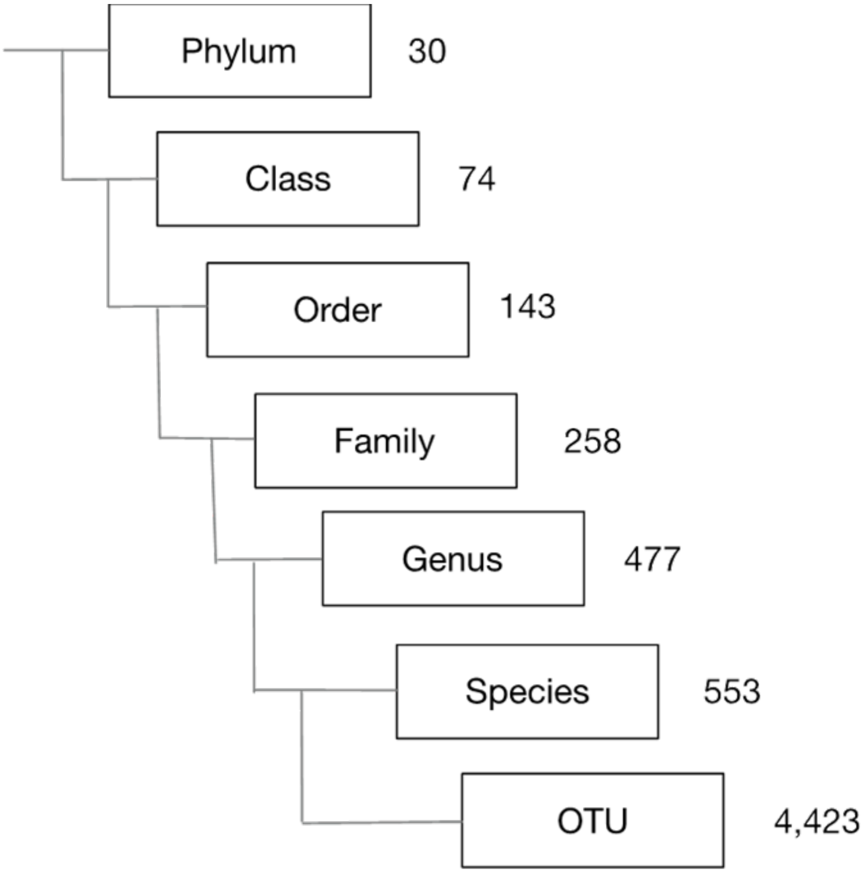

Numbers of uniquely annotated taxa against the Greengenes database within each clade identified from 4,423 operational taxonomic units (OTUs).

© 2021 Hsiao CJ et al. *JAMA Network Open.*

eFigure 2. Violin Plots of Variance Associated With Microbiome Cluster

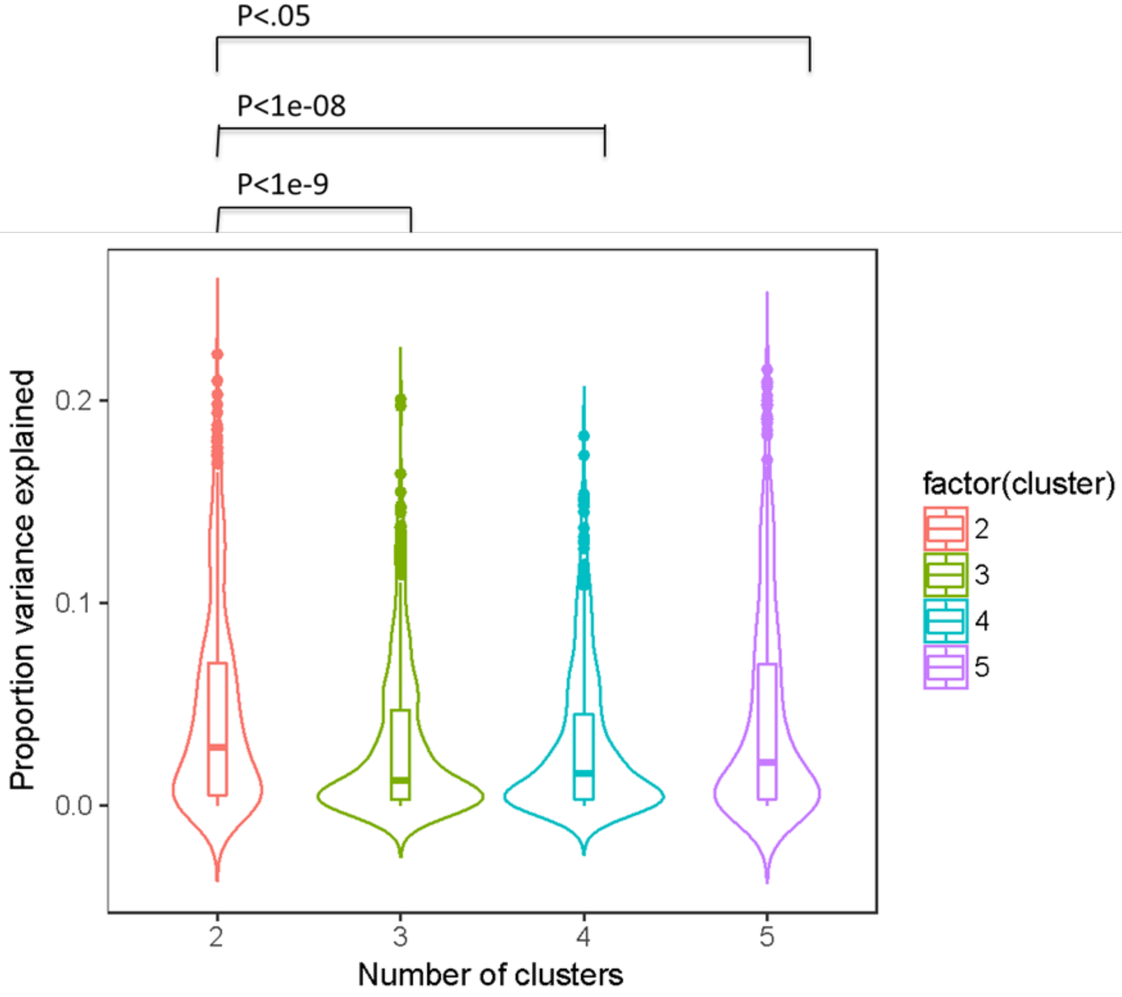

Along the abscissa are the number of clusters defined from K = 2 to K = 5. The ordinate represents the variance explained by cluster (within to between sum of squares). Cluster of K = 2 has the greatest (median) variability explained in comparison to other cluster representations and is the most parsimonious.

© 2021 Hsiao CJ et al. *JAMA Network Open.*

eFigure 3. Diversity Characterization of Nasal Microbiota by Cluster Class

eFigure 3A. Microbiome diversity metrics by cluster class: Alpha (Shannon) diversity

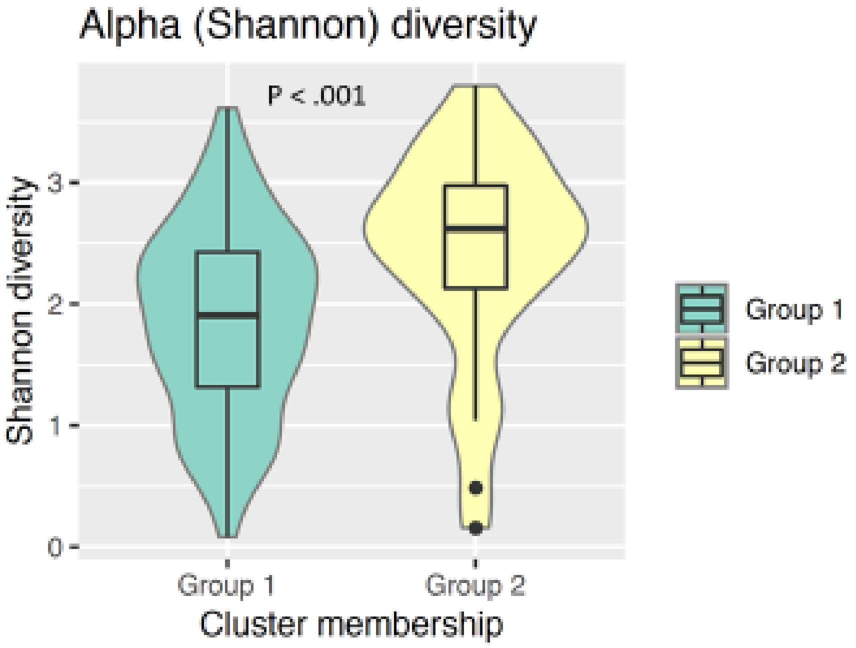

eFigure 3A shows violin plots describing sample Shannon diversity in each of the microbiome clusters. There is significantly less diversity within cluster1 (Group 1) compared to cluster2 (Group 2).

© 2021 Hsiao CJ et al. *JAMA Network Open.*

eFigure 3B. Microbiome diversity metrics by cluster class: Beta diversity

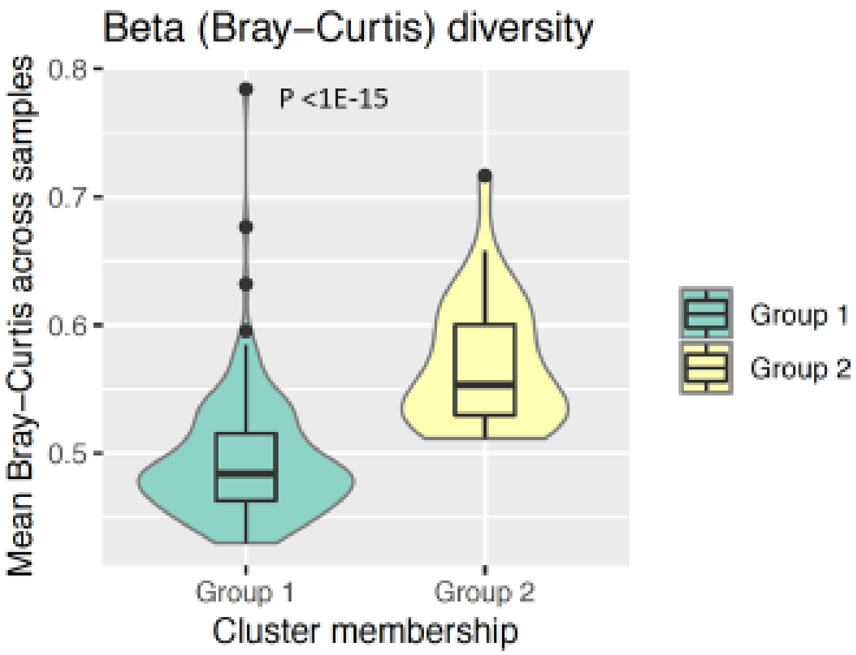

eFigure 3B shows violin plots describing the average Bray-Curtis distance (beta diversity) for each sample within by microbiome cluster. Cluster1 (Group 1) is a significantly more homogeneous (less diverse) than cluster2.

© 2021 Hsiao CJ et al. *JAMA Network Open.*

eFigure 3C. Microbiome diversity metrics by cluster class: Principal Components Analysis

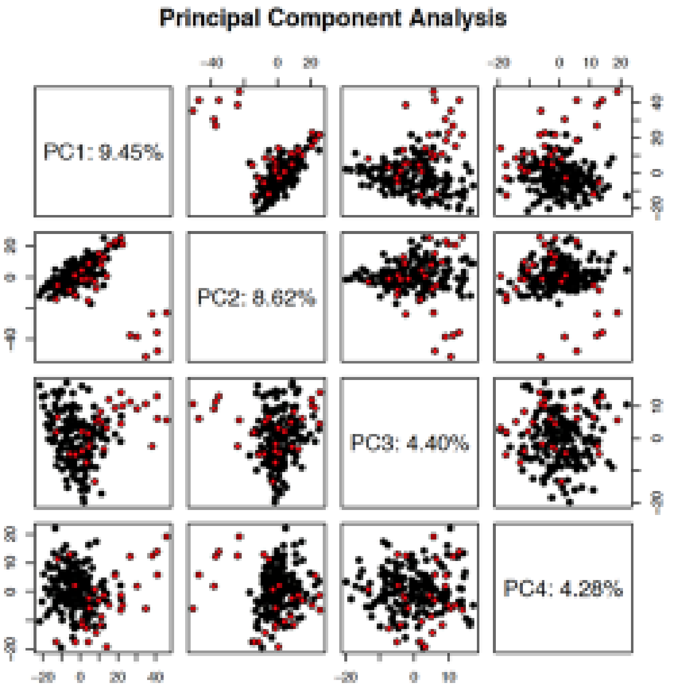

eFigure 3C is a pairwise scatterplot of the first through fourth principal components. Each point represents an individual patient sample, black= cluster1 (Group 1) and red= cluster2 (Group 2); proportion of variance explained by each principal component (PC) shown (%).

© 2021 Hsiao CJ et al. *JAMA Network Open.*
